# Two-factor synaptic consolidation reconciles robustness with pruning and homeostatic scaling

**DOI:** 10.1073/pnas.2422602122

**Published:** 2025-10-31

**Authors:** Georgios Iatropoulos, Wulfram Gerstner, Johanni Brea

**Affiliations:** ^a^School of Life Sciences and School of Computer and Communication Sciences, École Polytechnique Fédérale de Lausanne, Lausanne 1015, Switzerland

**Keywords:** memory consolidation, structural synaptic plasticity, attractor neural networks, sleep, synaptic volatility

## Abstract

Memory consolidation refers to a process of engram reorganization and stabilization that is thought to occur primarily during sleep through a combination of neural replay, homeostatic plasticity, synaptic maturation, and pruning. From a computational perspective, however, this process remains puzzling, as it is unclear how to incorporate the underlying mechanisms into a common mathematical model of learning and memory. Here, we propose a solution by deriving a self-supervised consolidation model that uses replay and two-factor synapses to encode memories in neural networks in a way that maximizes the robustness of cued recall with respect to intrinsic synaptic noise. We show that the dynamics of this optimization make the connectivity sparse and offer a unified account of several experimentally observed signs of consolidation, such as multiplicative homeostatic scaling, task-driven synaptic pruning, increased neural stimulus selectivity, and preferential strengthening of weak memories. The model also reproduces developmental trends in connectivity and stimulus selectivity better than previous models. Finally, it predicts that intrinsic synaptic noise fluctuations should scale sublinearly with synaptic strength; we find support for this in a meta-analysis of published synaptic imaging datasets.

## Significance

While most experiences are forgotten after only a few days, some memories can last an entire lifetime. The neurophysiological mechanisms that enable such memory preservation are poorly understood but are believed to be active during sleep, when neurons replay past events, prune their synapses, and regulate their firing. We provide a unified mathematical explanation for these processes in the form of an algorithm that stores memories in neural networks with maximal noise robustness. By representing each synapse as a product of two factors, our model automatically removes and tunes appropriate connections, while homeostatically scaling each neuron’s input. Our model reproduces experimental signs of activity-dependent rewiring and long-term memory formation in synaptic, cortical, and psychological data, and offers testable predictions.

The ability to store and retrieve remote memory is thought to rely on a distributed network of neurons located primarily in the cortical areas of the brain (1–4). This view is supported by anatomical studies, showing that cortical circuits are highly recurrent and, thus, particularly conducive to information storage (5–7). In an effort to unify these findings, models of long-term memory are today often based on the concept of attractor networks (8). The basic idea of this approach is to represent local cortical circuits with a recurrent neural network, in which each memory corresponds to a distinct pattern of activity that acts as an attractor of the network’s dynamics (9, 10).

In this context, memory encoding is modeled by configuring the connections of the network to imprint activity patterns as stable attractors. When this is done optimally, memory storage is saturated and the network reaches critical capacity (11, 12). This state is particularly significant. In a series of recent studies, attractor networks operating close to critical capacity have been shown to mimic several dynamical and structural motifs observed in cortical circuits, thereby suggesting that optimal storage is an organizing principle of cortical connectivity (13–16). However, it is unclear how such optimality can emerge in biology, and the precise role of synaptic plasticity in this process remains unknown.

In the experimental literature, the process whereby memories are stabilized and reshaped for long-term storage is generally referred to as consolidation. This takes place mainly during sleep (17) and is believed to be effected by a combination of neurophysiological mechanisms: Shortly after an initial episode of learning, cortical circuits undergo early tagging (18) and an immature engram is formed (19). This is accompanied by a rapid growth of new dendritic spines (20, 21). During sleep, the cortical engram is stabilized by replaying past neural activity (22–24) while task-irrelevant connections are pruned (21, 25, 26). At the same time, surviving synaptic connections are collectively scaled down (27–29) in order to maintain firing rate homeostasis (30). Notably, this regulation is multiplicative and, thus, preserves the relative differences between synapses (31).

Many of these aspects are neglected in standard attractor network models. Although phenomenological models have demonstrated that isolated aspects of consolidation, such as replay (32, 33), pruning (34), and homeostasis (35, 36), are beneficial for memory and learning, a principled account of the consolidation process within a common theoretical framework is lacking.

Here, we derive a normative synaptic plasticity model that reconciles the various biological mechanisms of consolidation with the notion of critical capacity in attractor networks. Our derivation is fundamentally based on a reformulation of the problem of critical capacity in two ways: First, instead of considering optimality to be a maximization of storage capacity (13–16), we define it as a maximization of memory robustness. Second, we assume that synapses are products of multiple subsynaptic components which form the expression sites for synaptic plasticity (36–39). The result is a self-supervised plasticity model that uses a combination of replay, homeostatic scaling, and Hebbian plasticity to prune connections and shape the network to perform noise-tolerant memory recall. The model offers a simple explanation for a wide range of putative consolidation effects observed in synaptic, neural, and behavioral data.

## Results

We construct our consolidation model in three steps: First, we introduce a specific parameterization of synaptic strength. Then, we define memory robustness. Finally, we combine these two parts to derive a learning rule that dictates how synapses should be modified to maximize memory robustness, that is, consolidate memory.

### The Circuit and Synapse Model.

**Fig. 1.**
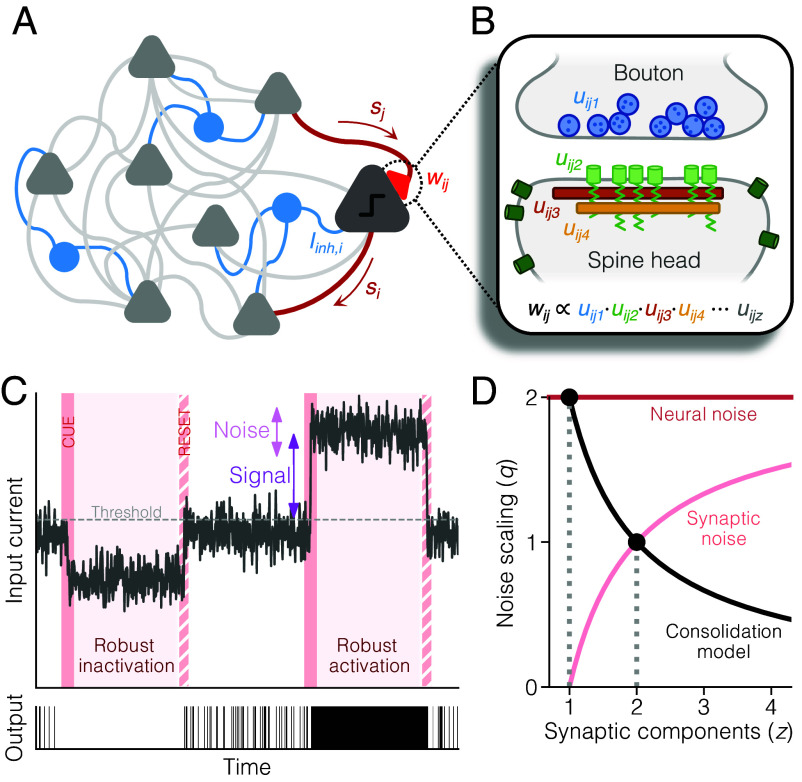
General model schematics. (*A*) Diagram of the circuit model. We consider a recurrent network of binary excitatory neurons with plastic nonnegative connection weights (gray). Note that inhibitory neurons are not explicitly modeled. Instead, inhibition is represented by a neuron-specific scalar input current $I_{\text{inh},i}$ (blue) that also undergoes plasticity. (*B*) Diagram of the synapse model. The total connection weight $w_{\mathrm{ij}}$ is a product of *z* factors $u_{ij1},\ldots ,u_{\mathrm{ijz}}$ that represent the efficacy of subsynaptic components, e.g., release probability (blue), receptor density (green), and scaffolding protein content (brown, orange). (*C*) Illustration of input current dynamics during idleness (white background) and recall (pink background) in a single neuron. The SNR during recall of a pattern is determined by the deflection of the mean input current from threshold, relative to the fluctuations caused by noisy afferent neurons or synapses. (*D*) The noise scaling exponent *q* as a function of *z* for neural and synaptic noise. Consolidation with *z* components maximizes robustness with respect to noise of type q=2/z, which is equivalent to neural noise when z=1, and synaptic noise when z=2.

We model a local circuit of cortical pyramidal cells using a recurrent network of *N* excitatory binary neurons (Fig. 1*A*). At every discrete time step *t*, each neuron i=1,…,N is characterized by an output state $s_{i}\left(t\right)$, which represents a brief period of elevated ($s_{i}=1$) or suppressed firing ($s_{i}=0$), similar to up and down states (40). The elevated state ($s_{i}=1$) occurs only if the neuron’s total input current $I_{i}\left(t\right)$ exceeds zero. The input current evolves in time according to

[1] $$\begin{array}{c} I_{i}\left(t+1\right)=\sum_{j=1}^{N}w_{\mathrm{ij}}s_{j}\left(t\right)-I_{\text{inh},i}\left(t\right), \end{array}$$

where the first term corresponds to the excitatory synaptic input from all neighboring neurons and $w_{\mathrm{ij}}\geq 0$ denotes the connection strength from neuron *j* to *i* (*SI Appendix*, *Circuit Model*). The second term is a scalar that represents the summed effect of all inhibitory inputs, as inhibitory neurons are not explicitly included in the model of the circuit.

In our mathematical analysis of the storage properties of the network, we focus on the connection strengths $w_{\mathrm{ij}}$. We begin by noting that the functional strength of a biological synapse (measured, for instance, as the amplitude of the postsynaptic potential) is an aggregate quantity that is determined by the interaction of several protein complexes that combine to form the internal synaptic structure (41). When long-term plasticity is induced, structural and chemical changes cascade throughout this molecular interaction network, causing the concentration and configuration of each component to be altered over the course of seconds to hours (42). This ultimately increases or decreases the synapse’s functional strength.

We model this internal synaptic structure by expressing each weight $w_{\mathrm{ij}}$ as the product of *z* subsynaptic components (factors) $u_{\mathrm{ijk}}$, where k=1,…,z, so that

[2] $$\begin{array}{c} w_{\mathrm{ij}}=\prod_{k=1}^{z}u_{\mathrm{ijk}}. \end{array}$$

Each variable $u_{\mathrm{ijk}}$ can be seen as the relative concentration of one or more subcellular building-blocks that are necessary to form a functional connection, for instance, the average concentration of released neurotransmitters or the density of postsynaptic receptors and scaffold proteins (Fig. 1*B*; see also *SI Appendix*, *Synapse Model* and ref. 36). We specifically consider one of the synaptic components ($u_{ij1}$) to be a flexible plasticity tag that is more volatile and sensitive to noise, while the remaining z−1 components represent more stable molecular processes that are active only during consolidation, consistent with tagging-and-capture dynamics (43, 44) (*SI Appendix*, *Extended Synaptic Noise Analysis*).

### Consolidation with Homeostatic Scaling, Synaptic Pruning, and Replay.

We define consolidation as the process of optimally storing a set of *M* memories, where each memory corresponds to a pattern of stationary network activity in which a specific group of neurons is active, while the rest is silent. The desired output of neuron *i* in pattern μ=1,…,M is defined by $\xi_{i}^{\mu }$, which is one with probability f≤0.5 and zero otherwise. We parameterize the storage load using the ratio α=M/N (where $\alpha_{c}$ denotes the highest possible load).

Prior to consolidation, the network is assumed to have undergone an initial episode of learning that has imprinted all patterns as stable attractors, albeit with suboptimal robustness. At this stage, patterns can only be recalled if the network operates with very low levels of noise. The purpose of consolidation is now to tune all connections so as to maximize robustness and allow patterns to be successfully recalled under much noisier conditions.

We define robustness as the largest amount of noise that can be tolerated by the neural population before an error occurs during recall (Fig. 1*C*). This is determined by the signal-to-noise ratio (SNR) of the weakest pattern. We can maximize this by having each neuron independently maximize a neuron-specific SNR where the signal is the amplitude of the input current deflection at the time the weakest pattern is recalled, i.e. $\min_{\mu }|I_{i}^{\mu }|\;=\;\min_{\mu }\;\left|\;\sum_{j}^{N}w_{\mathrm{ij}}\xi_{j}^{\mu }-I_{\text{inh},i}\right|$ (*SI Appendix*, *Memory Robustness*). Through iterative weight changes, the weakest pattern of neuron *i* will become more robust, and another pattern will then become the weakest and used to maximize the SNR. This process continues, until the lowest SNR across all patterns converges to a maximum.

The noise of the SNR is determined by the magnitude of random fluctuations in the input current. This varies depending on the source of noise that causes the fluctuations. Here, we expand on a previous analysis (45) and distinguish between two types of noise: neural noise and synaptic noise. Neural noise refers to perturbations of the neural states (the *s*-variables) caused either by the encounter of distorted stimuli or by faulty neural output activity (i.e., firing below the threshold or failing to fire above the threshold). Synaptic noise, on the other hand, refers to perturbations in the connectivity that, for example, are produced by spontaneous chemical reactions, conformational changes, or protein degradation and turnover (46, 47). We model these perturbations as white noise added to the volatile component $u_{ij1}$ in each connection (*SI Appendix*, *Noise Scaling*).

Input fluctuations caused by neural noise are proportional to $\sqrt{\sum_{j}w_{\mathrm{ij}}^{2}}$ and are therefore dependent on synaptic weight but not on the synaptic structural parameter *z*. The magnitude of synaptic noise, however, depends both on synaptic weight and structure, by scaling as $\sqrt{\sum_{j}w_{\mathrm{ij}}^{2-2/z}}$ (*SI Appendix*, *Noise Scaling*). We can therefore write a general expression for the SNR as

**Fig. 2.**
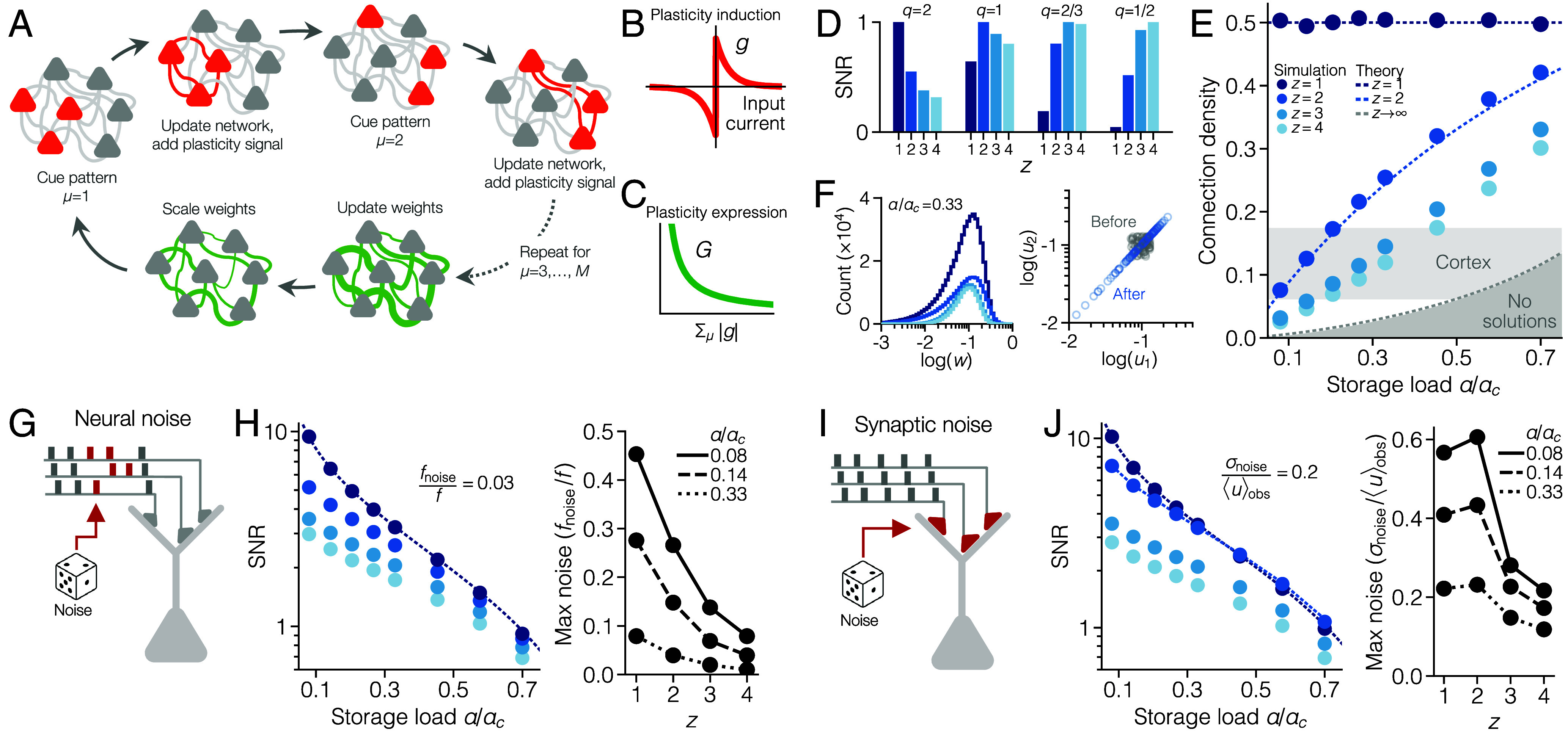
Simulated consolidation in networks with multifactor synapses. (*A*) Diagram of one replay cycle of the consolidation model, implemented in discrete time. (*B*) The gating function $g_{i}$. This determines the amplitude and sign of plasticity induction after replay of a single pattern (*SI Appendix*, *Consolidation Algorithm*). (*C*) The learning rate $G_{i}$. This determines the amount of plasticity expression after a full replay cycle and depends on the accumulated signal $\sum_{\mu }\left|g_{i}\right|$. (*D*) SNR (mean over $10^{3}$ neurons) for different combinations of noise scaling *q* and components *z*, at $\alpha /\alpha_{c}=0.08$. Weights are normalized to $\sum_{j}w_{\mathrm{ij}}^{q}=1$, and the maximal SNR, for a given *q*, is scaled to one. (*E*) Connection density. Circles represent simulations (mean over $10^{3}$ neurons) while dashed lines represent theoretical solutions (*SI Appendix*, *Theoretical Solutions*). The light gray area marks the connection probability (mean ± SEM) among cortical pyramidal cells in a meta-analysis of 124 experimental datasets from mice, rats, cats, and ferrets (16) (*SI Appendix*, *Data Analysis*). (*F*) *Left*: distribution of nonpruned weights [mean normalized to 0.1, colors as in (*E*)]. For distributions of all weights, see *SI Appendix*, Fig. S1. *Right*: the second synaptic components ($u_{ij2}$) plotted as a function of the first ($u_{ij1}$) in a simulated neuron with z=2, at $\alpha /\alpha_{c}=0.33$. (*G*) Illustration of neural noise. Each row of boxes represents binary input patterns at discrete time steps (gray = noise-free; red = distorted). (*H*) *Left*: SNR with respect to neural noise (q=2; the noise level is parameterized by $f_{\text{noise}}$; *SI Appendix*, *Noise Scaling*). *Right*: highest level of tolerated neural noise in tests of pattern recall (*SI Appendix*, *Numerical Optimization and Evaluation*). (*I*) Illustration of synaptic noise, which directly perturbs synaptic strengths. (*J*) *Left*: SNR with respect to synaptic noise (q=2−2/z; the noise level is parameterized by $\sigma_{\text{noise}}$). *Right*: highest level of tolerated synaptic noise in tests of pattern recall. All results in this figure are produced with f=0.5, but there is no qualitative change with low-activity patterns (*SI Appendix*, Fig. S2).

[3] $$\begin{array}{c} \text{SNR}\propto \frac{\min_{\mu }\left|I_{i}^{\mu }\right|}{\sqrt{\sum_{j}w_{\mathrm{ij}}^{q}}}, \end{array}$$

where the scaling exponent *q* is 2 for neural noise and 2−2/z for synaptic noise (Fig. 1*D*). The SNR can, in principle, be optimized (up to an arbitrary scaling factor) by any consolidation process that a) maximizes the signal and b) maintains a constant synaptic mass $\sum_{j}w_{\mathrm{ij}}^{q}$. The second property, however, requires a homeostatic weight regulation that is inhomogeneous across weights and, as such, directly at odds with the multiplicative homeostatic plasticity that has been observed experimentally (31) (*SI Appendix*, *Geometric Interpretation*). We resolve this issue by optimizing the SNR in terms of each neuron’s subsynaptic components $u_{\mathrm{ijk}}$, instead of directly treating the whole weight $w_{\mathrm{ij}}$. This results in the following three-step process (Fig. 2*A*; see *Materials and Methods* and *SI Appendix*, *Consolidation Algorithm* for more details):

1. Plasticity induction: All patterns are replayed. For each pattern *μ*, the network receives a cue and is updated (Eq. **1**) so that recall occurs. This triggers a plasticity signal $\delta u_{\mathrm{ijk}}^{\mu }=g_{i}\left(I_{i}^{\mu }\right)s_{j}\frac{w_{\mathrm{ij}}}{u_{\mathrm{ijk}}}$, which is accumulated in each neuron *i*. The function $g_{i}\left(I_{i}^{\mu }\right)=\pm e^{-\beta_{i}\left|I_{i}^{\mu }\right|}$ (where $\beta_{i}>0$ is a sharpness parameter) is a neuron-specific, input-dependent plasticity gate that determines the sign and amplitude of induced plasticity (Fig. 2*B*).
2. Plasticity expression: After the replay cycle, the accumulated plasticity signal is expressed by updating each component $u_{\mathrm{ijk}}$ with the increment $\Delta u_{\mathrm{ijk}}=G_{i}\sum_{\mu }\delta u_{\mathrm{ijk}}^{\mu }$, where $G_{i}$ is a neuron-specific learning rate that is regulated so that the total amount of expression is the same in each cycle (Fig. 2*C*). The plasticity signal is also used to adjust the inhibitory input $I_{\text{inh},i}$.
3. Homeostatic scaling: All $u_{\mathrm{ijk}}$ are scaled by a normalization factor, and the process starts over.

This consolidation model possesses a number of noteworthy mathematical properties: First, it is self-supervised, and requires no explicit error or target signal, as the target is provided by the response of the neurons themselves. Second, it maximizes the SNR for noise of type q=2/z (Figs. 1*D* and 2*D*). In other words, our consolidation model with *z*-component weights leads to the same solution as conventional single-component weights optimized with $L_{2/z}$ regularization (*SI Appendix*, *Consolidation Algorithm*). Our model, however, finds the solution with multiplicative homeostatic scaling, whereas a conventional implementation of $L_{2/z}$ would require inhomogeneous homeostatic changes (*SI Appendix*, *Geometric Interpretation*). As a result, networks that undergo consolidation with more subsynaptic components (higher *z*) end up with a larger fraction of zero-valued weights, also referred to as pruned weights (Fig. 2 *E* and *F* and *SI Appendix*, Fig. S1). Crucially, only networks with more than one component (z≥2) reach a connection probability low enough to be comparable to that in cortex, while the lowest connectivity produced using single-component weights is 50% (Fig. 2*E*; see also *SI Appendix*, *Theoretical Solutions*). Finally, the consolidation process also ensures that components within the same synapse align with each other, so that $u_{ij1}=u_{ij2}=\ldots =u_{\mathrm{ijz}}$ (Fig. 2*F*). This means that all components end up highly correlated with the total connection strength $w_{\mathrm{ij}}$, consistent with experimental findings (48, 49).

Networks with two-factor synapses (z=2) constitute a particularly important case. While consolidation with z=1 maximizes memory robustness with respect to neural noise (Fig. 2 *G* and *H*), consolidation with z=2 maximizes robustness with respect to synaptic noise (Fig. 2 *I* and *J*). In practice, this means that two-factor consolidation generates networks that are highly pruned yet, at the same time, at least as robust to synaptic noise as the densest networks (Fig. 2*J*). For z=2, the dynamics of the weights close to convergence can be described with the differential equation

[4] $$\begin{array}{c} \frac{\text{d}w_{\mathrm{ij}}}{\text{d}t}\propto [\;\underset{\text{homeostatic scaling}}{\underbrace{h(\sum_{j}w_{\mathrm{ij}})}}+\underset{\text{replay-induced LTP/LTD}}{\underbrace{G_{i}\left(t\right)\sum_{\mu }g_{i}(I_{i}^{\mu })\xi_{j}^{\mu }}}]\cdot w_{\mathrm{ij}}, \end{array}$$

where h(x) is a general homeostatic function that is negative when *x* exceeds a baseline, and positive otherwise. Importantly, all weight changes are now multiplicative, i.e., proportional to the momentary value of $w_{\mathrm{ij}}$. The homeostatic part, more specifically, performs a multiplicative $L_{1}$-regularization that both prunes a large fraction of the connections and scales the remaining ones to maintain a constant average strength. This, by extension, keeps the average input current constant as well (assuming a stable level of output activity in the network). The formulation in Eq. **4** is directly compatible with, and generalizes, previously proposed models of homeostatic plasticity (35, 36) (*SI Appendix*, *The Homeostatic Function*).

Note that our consolidation model is entirely derived from normative assumptions. This is equally true for the synapse model in Eq. **2**, which originates from a parameterization technique that implicitly biases an optimizer to find sparse solutions (50, 51). Ablating either the subsynaptic structure or the homeostatic scaling causes the model to fail (*SI Appendix*, Fig. S4).

### Consolidation Signs in Synaptic, Neural, and Behavioral Data.

In order to demonstrate how the consolidation algorithm can be incorporated into a single, self-supervised model of memory formation and stabilization, we simulate a network with two-factor synapses that optimally stores patterns across two phases of learning.

**Fig. 3.**
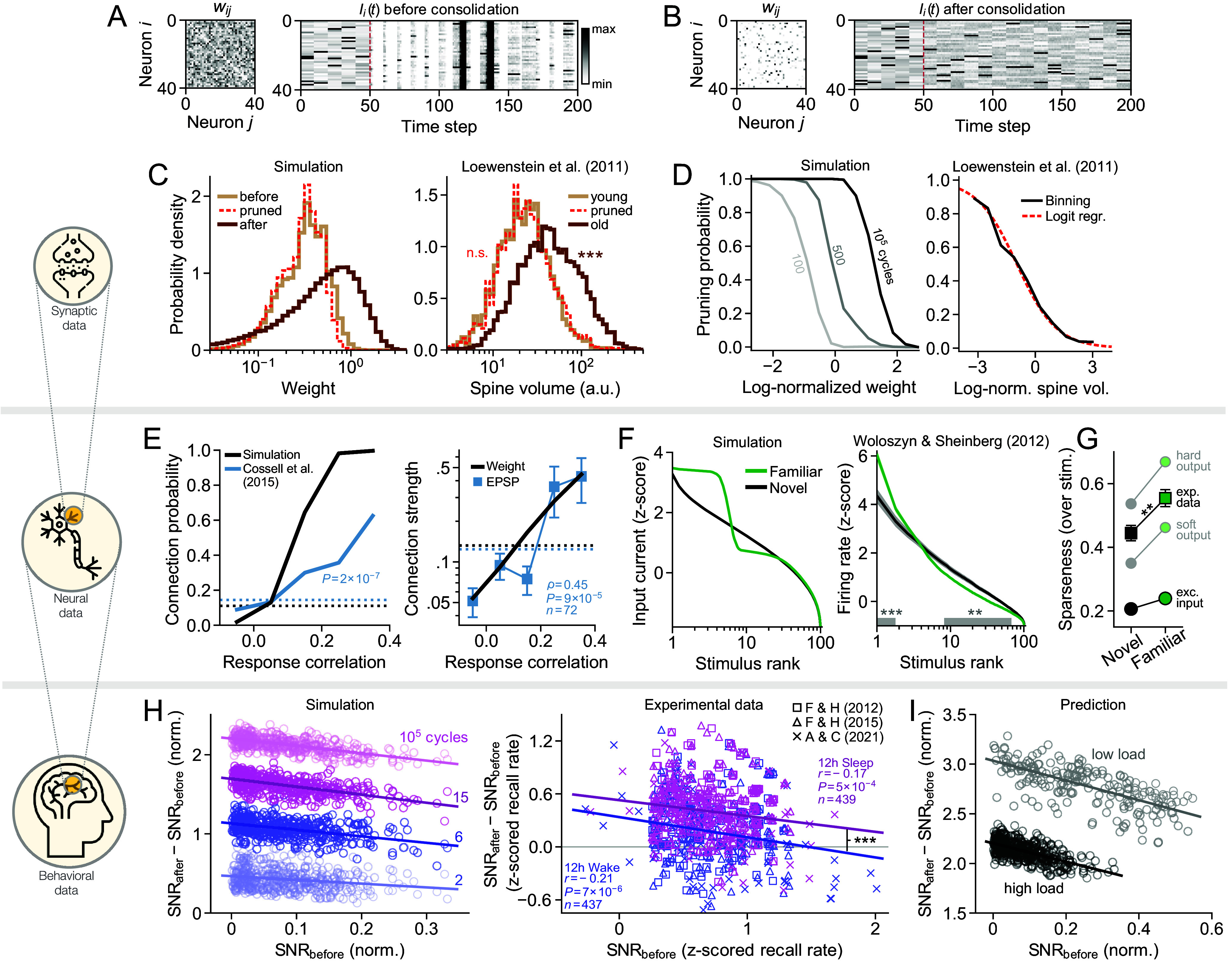
Signs of consolidation across three spatial scales. (*A*) Weight matrix (*Left*) and input current (*Right*) of 40 neurons during pattern recall, before consolidation (f=0.05, α=0.44). The network receives a cue every 10 steps and is then simulated for 10 steps. Synaptic noise starts after 50 steps (red line; $\sigma_{\text{noise}}/{\left\langle u\right\rangle }_{\text{obs}}=0.3$). (*B*) Same as (*A*), but after consolidation. (*C*) Distribution of weights (*Left*) and dendritic spine sizes on pyramidal cells in rodent cortex (52) (*Right*). (*D*) Pruning probability as a function of weight in simulated data (*Left*) and as a function of spine size in experimental data (*Right*). (*E*) Connection probability (*Left*) and connection strength (*Right*) as a function of binned response correlation among simulated neurons (black) and pyramidal cells in rodent visual cortex (53) (blue; error bars represent mean ± SEM). Dashed curves are grand averages. Connection strengths are normalized to have a maximum of one. (*F*) Tuning curves with respect to familiar and novel (previously unseen) stimuli, for simulated neurons (*Left*; mean over $10^{3}$ neurons) and for pyramidal cells in macaque inferior temporal cortex (54) (*Right*; mean ± SEM). (*G*) Tuning sparseness in simulations (circles; mean over $10^{3}$ neurons) and experimental data (squares; mean ± SEM). The hard and soft output is obtained by using sigmoidal activation functions with varying smoothness. (*H*) *Left* panel shows change in pattern SNR after simulated consolidation (circles are patterns) while *Right* panel shows change in human memory trace SNR after sleep (pink markers) and after wake (blue markers) (55–57). Behavioral data have been slightly jittered for clarity. (*I*) Change in pattern SNR after simulated consolidation with different loads. Stars indicate significance levels **P < 0.01 and ***P < 0.001.

In the first phase, representing wakefulness, the network starts fully connected and sequentially encounters external stimulus patterns that are imprinted as attractors using few-shot learning (*SI Appendix*, *Simulating Wakefulness and Sleep*). This leaves the network densely connected and sensitive to noise (Fig. 3*A*). In the second phase, the network undergoes consolidation, rendering the connectivity sparse and robust (Fig. 3*B*; see *SI Appendix*, Fig. S5 for details). This process represents the cumulative effect of multiple sleep sessions taking place over an extended period of time.

The simulation qualitatively reproduces a wide range of experimental observations linked to long-term plasticity (Fig. 3*C*–*I*; note, however, that simulated effects generally are more amplified, as we model a long stretch of biological time with a single bout of optimal consolidation). On the synaptic level, simulated wakefulness produces relatively small weight perturbations, while sleep entails more extensive rewiring in which a majority of existing weights are pruned. The distribution of presleep weights therefore closely overlaps with the distribution of pruned weights (Fig. 3*C*, *Left*), while surviving weights generally are stronger. We find analogous results in experimental data (52) (Fig. 3*C*, *Right*). The distribution of dendritic spine volume for young spines (age ≤4 d) is statistically indistinguishable from that of pruned spines, while old spines (age >4 d) are significantly larger (Kolmogorov–Smirnov tests, $P_{\text{pruned}}=0.61$, $P_{\text{old}}=8.6\times 10^{-190}$, $n_{\text{young}}=2,268$, $n_{\text{pruned}}=2,300$, $n_{\text{old}}=5,011$). Simulated networks with single-component weights, however, do not undergo enough pruning to replicate this effect (*SI Appendix*, Fig. S7*A*).

An analysis of individual weight trajectories reveals that the probability of pruning decreases as a function of strength, meaning that connections that are potentiated prior to consolidation have higher chances of surviving (Fig. 3*D*, *Left*). This trend is, again, present and highly significant in the experimental data (52) (logistic regression with two-tailed *t*-test, $P=1.5\times 10^{-195}$, n=7,311; Fig. 3*D*, *Right*).

Next, we analyze how weights are configured depending on neural response similarities. Using the total excitatory input current $\sum_{j}w_{\mathrm{ij}}s_{j}$ as an indicator of graded output activity, we find that neurons are more likely to stay connected after consolidation if their responses during recall are correlated (Fig. 3*E*, *Left*). Similar synaptic selectivity is seen in experimental measurements of visual cortical neurons in mice during static image presentations (53) (two-sided Cochran–Armitage trend test, $P=1.7\times 10^{-7}$, n=520). The average connection strength also increases with response correlation, both in simulated and experimental data (Spearman’s ρ=0.45, $P=8.7\times 10^{-5}$, n=72; Fig. 3*E*, *Right*). Networks with single-factor synapses, however, fail to match experimental statistics (*SI Appendix*, Fig. S7*B*).

Another direct consequence of our consolidation model is an increased neural stimulus selectivity. Each neuron’s response to the stored patterns is enhanced by moving the input current further away from the threshold. This sharpens the tuning curve for familiar (consolidated) patterns relative to novel ones (Fig. 3*F*, *Left*; *SI Appendix*, *Stimulus Tuning*). The same phenomenon can be observed in the activity of inferotemporal cortical pyramidal cells of Macaques, measured during the presentation of familiar and novel images (54) (Welch’s *t*-test, ∗∗*P* < 0.01, ∗∗∗*P* = 1.5 × 10^−5^, *n* = 73; Fig. 3*F*, *Right*). The sharpness of the tuning curve is quantified by the sparseness, a metric that is near zero when all stimulus responses are similar, and near one when responses are selective to very few stimuli (*SI Appendix*, *Stimulus Tuning*). The sparseness increases significantly during stimulus familiarization (Welch’s *t*-test, $P=2.9\times 10^{-3}$, n=73; Fig. 3*G*).

On the behavioral level, sleep has been shown to enhance the ability to recall recently formed declarative memory (58), in a way that suggests larger improvements for items with weaker initial encoding (59). Our model demonstrates this effect when the change in SNR for each pattern is evaluated over the course of simulated consolidation (Fig. 3*H*, *Left*). Patterns that start off weak consistently benefit more than those starting strong (negative correlation), while a longer period of replay produces a stronger average encoding (curve shifts upward). The slope is caused by a ceiling effect: As the SNR of each pattern is pushed to a maximum, weak patterns inevitably exhibit a larger improvement than strong ones.

For a qualitative comparison with experimental evidence, we pool and reanalyze three large, published datasets on sleep-based consolidation of declarative memory (55–57). In each study, humans memorize 40 word pairs and recall is tested before and after 12 h of wakefulness or sleep. We estimate the memory SNR in each subject as the z-scored recall rate, and then compute the change between the two test sessions. The result (Fig. 3*H*, *Right*) confirms that gains in SNR are higher for subjects with weaker initial encoding, both after wakefulness (Pearson’s r=−0.21, $P=6.7\times 10^{-6}$, n=437) and sleep (r=−0.17, $P=4.6\times 10^{-4}$, n=439). There is no significant difference in the slopes (*t*-test, P=0.49, n=876), but sleep-gains are systematically higher across all initial performance levels (*t*-test, $P=3.9\times 10^{-4}$, n=876). Our model suggests a conceptual explanation to these findings, where the slope is caused by a ceiling in memory strength while the vertical shift results from a difference in consolidation duration or efficacy between sleep and wake. The model predicts that a similar systematic shift also should be observed when changing the length of the word list (Fig. 3*I*).

### Implications for Lifelong Learning.

To model the effects of consolidation over developmental timescales (typically months or years), we start from the assumption that memory load increases with age as a response to lifelong learning (see also refs. 14 and 15). Accordingly, we liken cortical circuits at different stages in life to networks that have consolidated varying amounts of memory. We also use this model to represent cortical development under conditions of low or high environmental richness.

**Fig. 4.**
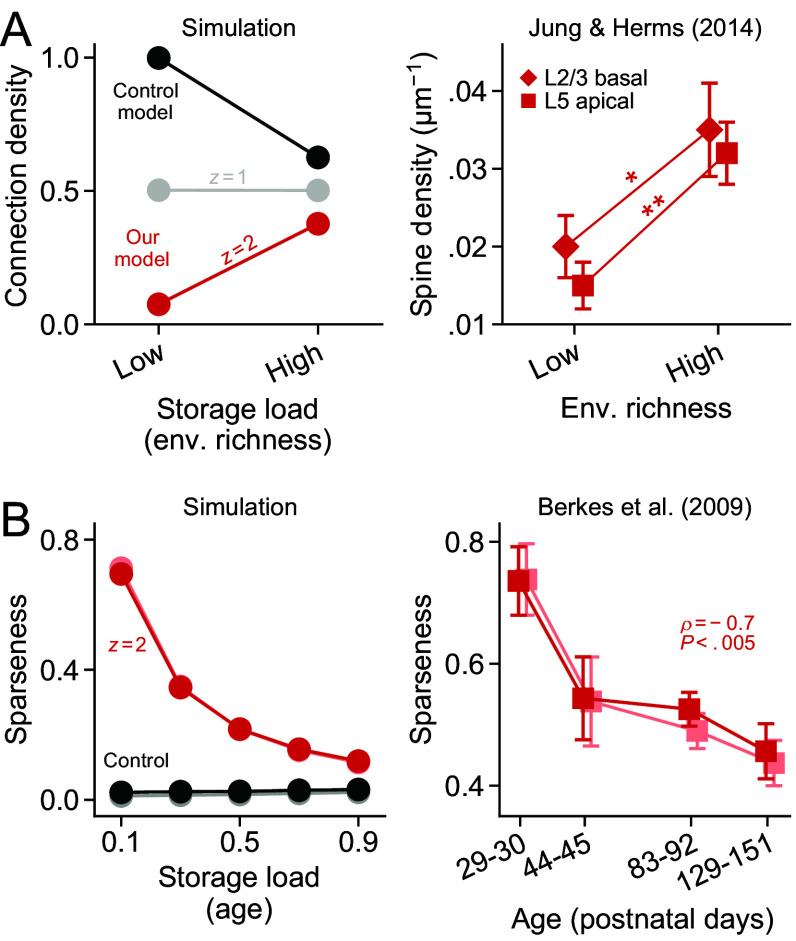
Signs of consolidation across development. (*A*) *Left*: Connection density as a function of storage load (indirect indicator of environmental richness) after consolidation with our model (z=1,2; same as Fig. 2) and with a control model that maximizes storage load instead of SNR (*SI Appendix*, *Control Model*). *Right*: Density of stable dendritic spines (age > 3 wk) in somatosensory cortex of rodents kept in environments of low and high stimulus richness since infancy (60). Stars indicate significance levels ^∗^*P* < 0.05 and ^∗∗^*P* < 0.01. (*B*) *Left*: Sparseness across stimuli (red, black) and across neurons (pink, gray; *SI Appendix*, *Stimulus Tuning*) as a function of storage load (indirect indicator of age) after consolidating low-activity patterns (f=0.05) with our model (z=2) and the control model. *Right*: Sparseness across time (red) and across neurons (pink) for neurons in visual cortex of ferrets at different stages of development (61). Circles represent mean over $10^{3}$ simulated neurons while squares represent experimental data (mean ± SEM).

According to our model, circuits that optimally store larger number of memories require a higher density of connections (Fig. 4*A*, *Left*). This is a direct consequence of maximizing SNR under sparseness constraints (see Fig. 2*E*, z≥2). This is consistent with the elevation in dendritic spine density that has been observed in animals raised in stimulus-enriched environments (60) (Student’s *t*-test, $P=5.7\times 10^{-3}$, $n_{\text{low}}=5$, $n_{\text{high}}=6$ for layer 5; P=0.035, $n_{\text{low}}=4$, $n_{\text{high}}=4$ for layer 2/3; Fig. 4*A*, *Right*). The experimental finding cannot be reproduced if we alter the consolidation model to maximize storage capacity instead of SNR, as has been suggested in past theoretical work (14–16, 62) (Fig. 4*A*, black; *SI Appendix*, *Control Model*). The effect is also occluded when using single-factor synapses (Fig. 4*A*, gray).

Networks that optimally store more memories also exhibit flatter tuning profiles and, thus, decreased sparseness (Fig. 4*B*, *Left*). This is a fundamental property of our consolidation algorithm, caused by the decrease in the maximum attainable SNR with load (Fig. 2 *H* and *J*). The effect is analogous to the decline in sparseness that has been measured in visual cortical neurons of ferrets at different stages of development, from eye-opening to adulthood (61) (Spearman’s ρ=−0.69, $P=2.9\times 10^{-3}$, n=16 for sparseness across time; ρ=−0.67, $P=4.5\times 10^{-3}$, n=16 for sparseness across neurons; Fig. 4*B*, *Right*). This trend cannot be reproduced with a network that maximizes storage capacity instead of SNR (Fig. 4*B*, black).

### Scaling of Intrinsic Synaptic Noise.

Our consolidation model crucially relies on the parameterization of each synaptic weight $w_{\mathrm{ij}}$ as a product of multiple components $u_{\mathrm{ijk}}$. Is it possible to detect signatures of such synaptic ultrastructure in available experimental data? To answer this, we first note that a key prediction of our model can be found in the synaptic noise scaling. When the volatile component $u_{ij1}$ is subjected to random perturbations, the weight of the synapse, as a whole, fluctuates with an amplitude $\Delta w\propto w^{1-1/z}$. For two-factor synapses, this reduces to

[5] $$\begin{array}{c} \Delta w\propto \sqrt{w}. \end{array}$$

Stated more generally, our model predicts that synapses with more than one component display intrinsic noise that scales sublinearly with weight, both for potentiation and depression. Sublinear scaling also holds true when all components $u_{\mathrm{ijk}}$ are subject to noise perturbations (*SI Appendix*, *Extended Synaptic Noise Analysis*). It is only in the limit of infinitely many components (z→∞) that the noise magnitude becomes proportional to the weight. Conversely, only single-component synapses produce noise that is additive and uncorrelated to weight.

To validate this prediction with an artificial synaptic dataset, we model the internal structure of a synapse as a stochastic dynamical system, and use this to simulate the evolution of 1,000 independent synapses through time (*SI Appendix*, *Simulating Synaptic Intrinsic Noise*).

**Fig. 5.**
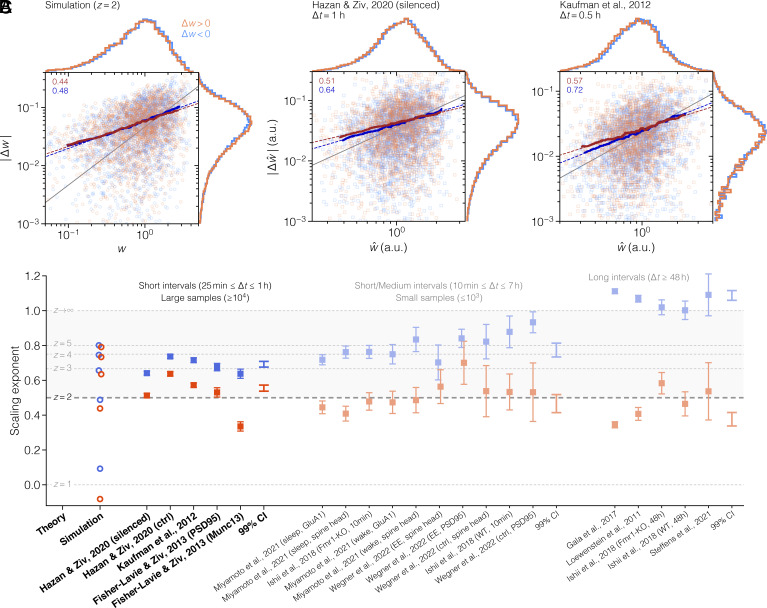
Scaling of synaptic fluctuations. (*A*) Absolute weight change as a function of initial weight in simulated data with z=2, for potentiation (orange) and depression (blue); see also *SI Appendix*, Figs. S8*A* and S9*A*. Solid lines are the results of moving averages, and dashed lines are linear fits to the solid lines (slope value shown in *Upper Left* corner). The identity line (gray) has slope 1 and is included for comparison. (*B*) The same type of plot as in (*A*) but for experimentally measured dendritic spine sizes in rodent cortical neurons (63, 64) (*SI Appendix*, Figs. S8*B* and S9*B*). (*C*) The scaling exponent of synaptic fluctuations in simulated (circles) and experimental data (squares; mean ± SE). This is the slope of the average fluctuation size in logarithmic space, obtained with bootstrapped linear regression (see *SI Appendix*, Fig. S10 for additional regression results). Labels on the abscissa contain a publication reference and a brief methodological descriptor; complete details are provided in *SI Appendix*, Tables S5–S7.

The data are analyzed by plotting the absolute weight change |Δw(t)|=|w(t+Δt)−w(t)| as a function of the initial weight w(t) and then applying a moving average to detect underlying trends in the scattered data (Fig. 5*A*; see *SI Appendix*, Fig. S8*A* for more examples). Consistent with our theory, the average noise amplitude ⟨|Δw|⟩ increases linearly in a log–log plot, both for depression (Δw<0) and potentiation (Δw>0). This indicates a power-law relation in the original data, i.e. $\left\langle |\Delta w|\right\rangle \propto w^{x}$, where the exponent *x* is equivalent to the slope of the line in logarithmic space. We estimate this slope by applying bootstrapped linear regression to the trend line (*SI Appendix*, *Synaptic Noise Scaling*) and find that it agrees with theoretical predictions (Fig. 5*C*). Applying linear regression to the root mean square deviation or directly on the raw data yields virtually the same results (*SI Appendix*, Figs. S9*A* and S10).

To test our prediction on experimental data, we compile 20 published synaptic datasets from 9 separate studies (29, 52, 63–69). These publications span more than a decade of research and employ fluorescence microscopy and superresolution nanoscopy in both cultured neurons and live animals, under various environmental conditions (see *SI Appendix*, Tables S5–S7 for details). Common to all studies, however, is that they measure an indirect indicator of synaptic strength (denoted $\hat{w}$) in a large population of synapses that have been individually tracked over extended periods of time (ranging from 24 h to almost 30 d).

We reanalyze each dataset according to the procedure described above. In the two largest datasets, shown as examples in Fig. 5*B*, the average noise magnitude exhibits a clear linear dependence on the synaptic strength in logarithmic space, again indicating an underlying power-law like that found in simulations (similar results are reported in refs. 67 and 70; see *SI Appendix*, Figs. S8*B* and S9*B* for more examples). The estimated noise scaling exponent for each dataset is presented in Fig. 5*C* and *SI Appendix*, Fig. S10.

For large datasets with high sampling frequencies (i.e., short sampling intervals Δt≤1h; Fig. 5*C*, first group of data), synaptic fluctuations consistently have a sublinear scaling, with an exponent of 0.56±0.02 for potentiation and 0.69±0.02 for depression (99% weighted CI). These estimates are remarkably reliable and close to the range predicted by our synaptic noise model with z=2 and 3. Note, however, that our model only describes intrinsic noise, which is best measured in conditions when activity-dependent synaptic plasticity is either negligible or entirely blocked. Theoretical predictions are therefore only approximately applicable to the experiments, which, in almost all cases, contain extrinsic synaptic noise. The data by Hazan and Ziv (64) is a notable exception, as this was acquired while glutamatergic transmission was pharmacologically blocked. In this case, the noise scaling almost exactly matches the theoretical lines for z=2 and 3, as we obtain 0.51±0.01 for potentiation and 0.64±0.01 for depression (mean ± SE, bootstrap of 100 samples; Fig. 5*B*, *Left*).

In datasets with smaller sample sizes (Fig. 5*C*, second group) and longer sampling intervals (Fig. 5*C*, third group), the scaling exponent generally increases for depression and decreases for potentiation (Fig. 5*C*, second and third CIs; *SI Appendix*, *Extended Synaptic Noise Analysis*).

### Signs of Homeostatic Scaling in Synaptic Noise.

**Fig. 6.**
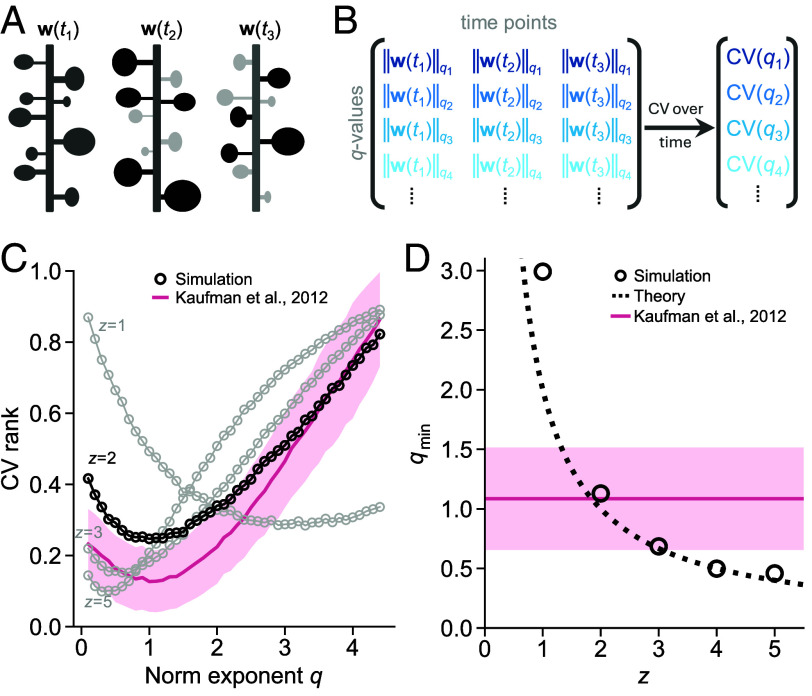
Signs of homeostatic scaling in synaptic noise. (*A*) Diagram of dendritic spines observed at three time points $t_{1}$, $t_{2}$, and $t_{3}$. The spine sizes (denoted with vector w) fluctuate as some spines grow (black) and others shrink (light gray). (*B*) The norm of spine sizes (${\left||w|\right|}_{q}$) is calculated at each time point (column) using different *q*-values (rows). The fluctuation of a norm is then quantified by the CV over all time points. (*C*) The CV of norms, ranked from zero to one, as a function of *q*, using simulated data (black, gray) and dendritic spine sizes (pink) from pyramidal cells in rodent cortex (63) (mean ± SE, bootstrap of 1,000 samples; see also *SI Appendix*, Fig. S12). (*D*) The *q*-value at which the CV is minimized (denoted $q_{\text{min}}$).

Our plasticity model does not only govern the trajectory of individual synapses, but it also shapes the distribution of synaptic populations. Recall that our model includes homeostatic scaling that, close to optimal storage, maintains a constant synaptic mass $\sum w^{2/z}$. The implication is that the weight distribution, in the absence of activity-dependent plasticity, exhibits a constant $\frac{2}{z}$-th moment. To confirm this numerically, we return to the simulated synaptic data and estimate the stability of different moments of the weight distribution by calculating the coefficient of variation (CV) of the norm ${\left(\sum w^{q}\right)}^{1/q}$ across time (Fig. 6 *A* and *B*). Consistent with theory, we find that the weight norm that varies least over time (i.e., has lowest CV rank) roughly follows the relation $q_{\text{min}}=2/z$ (Fig. 6 *C* and *D*).

We test this prediction using the experimental data by Kaufman et al. (63), composed of 1,087 dendritic spines measured every 30 min over a total of 24 h. At each measurement, we calculate the norm of spine sizes, followed by the CV of the norm across time. The result, plotted as a function of *q*, displays a U-shaped curve that is best matched by the two-factor model ($R^{2}=0.67$ for z=2, compared to second best $R^{2}=0.64$ for z=3; Fig. 6*C*, pink curve). The CV is lowest at $q_{\text{min}}=1.09\pm 0.43$, close to the theoretical prediction for z=2 (Fig. 6*D*, pink line; see also *SI Appendix*, Fig. S12). The same analysis of Hazan and Ziv’s data (64) yields noisier results, but the z=2 model is, again, the closest match (*SI Appendix*, Fig. S13).

## Discussion

We have derived a general mathematical model of synaptic consolidation that optimizes for noise-robust recall of attractor memories in recurrent neural networks with factorized multicomponent synapses. The contribution of our work is two-fold: First, it demonstrates that the various mechanisms underlying consolidation can be derived from first principles, within a single model of optimal memory storage. Second, by linking optimality to synaptic plasticity and the concept of critical capacity, it offers an explanation of how the structured connectivity of optimal attractor networks (14–16) might emerge in cortical circuits.

In the special case of two-factor synapses, our plasticity model has a particularly simple form, in which all updates are multiplicative, both in terms of subsynaptic factors *u* and the whole synaptic weight *w*. Despite this, a large fraction of connections are pruned while the average strength of surviving synapses is homeostatically regulated. This resolves a contradiction in past synaptic plasticity studies: Sparse connectivity, like that measured in neocortex (6), has been difficult to reconcile with multiplicative homeostatic scaling (31), given that Hebbian plasticity with multiplicative constraints tends to produce dense solutions (71). Sparse solutions for single-component weights typically require constraints that are either additive (16, 34, 71, 72) or that impose hard thresholds (34). This generally requires hyperparameter-tuning prior to learning (*SI Appendix*, *Geometric Interpretation*). The introduction of multicomponent synapses, however, reconciles the need for sparsification with multiplicative homeostatic plasticity. Two-factor synapses, in particular, also maximize the ability of a network to recall patterns under intrinsic synaptic noise while exhibiting noise scaling characteristics that match experimental data better than the conventional unitary synapse model. These findings are compatible with the wider neuroscientific literature, where average synaptic strength often is computed as a product of two or three factors, such as the vesicle release probability, number of release sites, and the quantized vesicle size (49).

Our results suggest that synaptic structural complexity serves a computational and metabolic purpose by implicitly biasing connectivity to be sparse, thereby lowering energy consumption and freeing unneeded synaptic resources for future learning. As such, our work is complementary to recent studies analyzing the effects of the synaptic ultrastructure on memory stability (73, 74), consolidation (43, 75), and energy consumption (76) (see also ref. 44).

We interpret our consolidation model as a general theory of sleep by situating it in the following scenario: During wakefulness, the network undergoes intense sensory-driven stimulation which imprints neural activity patterns as attractors. These are initially labile and, thus, represent immature engrams that are difficult to recall and are easily erased by spurious plasticity. During sleep, external inputs are silenced and patterns can be replayed. The process of consolidation now serves to tune connectivity in a way that enlarges all basins of attraction and pushes the network to critical capacity. This stabilizes the engrams and makes them resilient to structural and sensory perturbations.

Our model relies on a self-supervised replay mechanism that first reinstates memories sequentially, and thereafter modifies the synapses. This implies that memories must be recallable prior to sleep and that replay must be significantly faster than plasticity expression. Both requirements are supported by experimental observations (59, 77).

Our account of sleep-based consolidation offers an alternative to an earlier theory of sleep (78, 79), where replay is used to unlearn spurious attractors with anti-Hebbian plasticity in order to indirectly increase the robustness of desired memories. By contrast, our model is Hebbian and accomplishes the same goal by replaying only information that already is familiar, without having to identify spurious patterns.

The plasticity rule that forms the core of our consolidation model can be tested in synaptic, neural, and behavioral data. On the synaptic level, the model predicts that the internal structure of a synapse manifests itself as a sublinear scaling of intrinsic noise fluctuations. For two-factor synapses, this specifically means that noise scales as $O(\sqrt{w})$. We emphasize, however, that an accurate analysis of synaptic noise requires a high sampling frequency and silencing of neural activity. Estimates of noise scaling are uninformative if the time between measurements is too long, as this only provides a temporal average that obscures the dynamics of instantaneous fluctuations, which are nonlinear and state-dependent. Similarly, the analysis of homeostatic scaling effects in synaptic norms should ideally be performed on measurements from synapses that are affected by the same homeostatic mechanism and are under blocked signaling.

On the neural level, our plasticity model requires a gating function that predicts that patterns linked to novel, immature, or otherwise weak memories induce higher levels of plasticity, compared to patterns representing highly familiar memories.

Finally, on the behavioral level, we predict that memories that are weakly encoded prior to sleep generally display a larger improvement in SNR (and in the rate of recall) after sleep. While we partly confirm this with three large, published datasets, these cover only a part of the range of initial encoding. Moreover, our model predicts that the average recall performance should shift downward when subjects are required to memorize more information, and vice versa.

We anticipate that our normative account of synaptic consolidation will contribute to a better understanding of long-term memory by inspiring neurobiologists to test the model in future experiments.

## Materials and Methods

We consider *N* binary neurons indexed i=1,…,N with output $s_{i}\in \left\{0,1\right\}$ and update dynamics $s_{i}\left(t+1\right)=\;\Theta \left(\sum_{j=1}^{N}w_{\mathrm{ij}}s_{j}\left(t\right)-I_{\text{inh},i}\right)$, where Θ is the Heaviside function, $w_{\mathrm{ij}}\geq 0$ are synaptic strengths, and $I_{\text{inh},i}$ is a neuron-specific inhibitory input current. During wakefulness, *M* activity patterns $\xi_{i}^{\mu }\in \left\{0,1\right\}$ are stored such that $\xi_{i}^{\mu }=\;\Theta \left(\sum_{j=1}^{N}w_{\mathrm{ij}}\xi_{j}^{\mu }-I_{\text{inh},i}\right)$ holds for all μ=1,…,M. For sufficiently small load M/N, this can be achieved with a simple autoassociative “Hopfield-like” rule (10) with nonnegative weights (*SI Appendix*, *Simulating Wakefulness and Sleep*). Although all patterns are stable fixed points, they are not particularly noise robust, in the sense that small perturbations of the weights or neural activities may prevent recovery of the stored patterns. We assume that the goal of consolidation is to make the attractors more noise robust.

Naive robustification can be achieved by maximizing the margin $\frac{\min_{\mu }\left(2\xi_{i}^{\mu }-1\right)\left(w_{i}\cdot \xi^{\mu }-I_{\text{inh},i}\right)}{\|w_{i}{\|}_{2}}$, i.e. the $L_{2}$-distance between the threshold and the closest pattern (support vector), where we use vector notation $w_{i}=\left(w_{i1},\ldots ,w_{\mathrm{iN}}\right)$, $\xi^{\mu }=\left(\xi_{i}^{\mu },\ldots ,\xi_{N}^{\mu }\right)$, and $\|w_{i}{\|}_{2}=\sqrt{\sum_{j}w_{\mathrm{ij}}^{2}}$ is the L2-norm. This is equivalent to maximizing $\text{SNR}\left(q\;=\;2\right)\propto \frac{\min_{\mu }\left|w_{i}\cdot \xi^{\mu }-I_{\text{inh},i}\right|}{\|w_{i}{\|}_{2}}$, i.e. the signal-to-noise ratio of the weakest pattern. The batch-perceptron algorithm (80) can be used to solve this optimization problem iteratively, by determining in each iteration the support vector, incrementally changing the weights to make it more robust, and rescaling the weights to maintain constant $L_{2}$ weight norm. The method has, however, two features that are difficult to defend from a biological perspective: i) the resulting connectivity is dense, and ii) the weakest pattern needs to be tagged in each iteration.

The first issue can be addressed by modifying the batch-perceptron algorithm to maximize the $L_{1}$-margin, i.e. maximize SNR(q = 1). Naive weight regularization to maintain constant $L_{1}$-norm, however, results in a homeostatic process that is incompatible with multiplicative scaling (*SI Appendix*, *Geometric Interpretation*). Based on insights from the optimization literature (50), we reparameterize each weight as a product of two factors, i.e. $w_{\mathrm{ij}}=u_{ij1}u_{ij2}$, and apply the standard batch-perceptron algorithm to the individual factors. This enables the maximization of SNR(q=1) with multiplicative homeostatic scaling.

The second issue is addressed by approximating the artificial tagging of the weakest pattern in each iteration of the batch-perceptron algorithm with an online version. This is achieved with the gating function $g_{i}$ and the learning rate $G_{i}$. When combined, these act as a soft argmin function since $G_{i}\cdot g_{i}\left(I_{i}^{\mu }\right)\propto \frac{\exp(-\beta_{i}|I_{i}^{\mu }\|)}{\sum_{\mu }\exp(-\beta_{i}|I_{i}^{\mu }\left|\right)}$, where $I_{i}^{\mu }=w_{i}\cdot \xi^{\mu }-I_{\text{inh},i}$ (*SI Appendix*, *The Gating Function*).

Although our consolidation model is presented as a method for robustly storing attractors in recurrent neural networks, all learning procedures and reasoning steps generalize to feedforward architectures. In this case, any binary input–output relation $y_{i}^{\mu }=\;\Theta \left(\sum_{j=1}^{N}w_{\mathrm{ij}}\xi_{j}^{\mu }-I_{\text{inh},i}\right)$ that is initially stored with low noise tolerance can, through the same weight consolidation, be made more robust. In *SI Appendix*, Fig. S6, we show an example where a sequence of patterns is learned in this way.

## Supplementary Material

Appendix 01 (PDF)

## Data Availability

Code data have been deposited in GitHub (https://github.com/geoiat/2f-syn-con). All other data are included in the manuscript and/or *SI Appendix*. Previously published data were used for this work (16, 29, 52–57, 60, 61, 63–69).
