# Supplementary material for: Two-factor synaptic consolidation reconciles robustness with pruning and homeostatic scaling
Source: Proc Natl Acad Sci U S A. 2025 Oct 31;122(44):e2422602122. doi: 10.1073/pnas.2422602122 (PMC12595459; doi:10.1073/pnas.2422602122)
Supplement: Supplementary file 1 — Appendix 01 (PDF) [file pnas.2422602122.sapp.pdf]

Supporting Information for

Two-factor synaptic consolidation reconciles robustness with pruning and homeostatic scaling

Georgios Iatropoulos, Wulfram Gerstner, and Johanni Brea

Corresponding Author: Georgios Iatropoulos.
E-mail: georgios.iatropoulos@gmail.com

This PDF file includes:

Supporting text
Figs. S1 to S17
Tables S1 to S7
SI References

Supporting Information Text

Contents

S.1 Model details . . . 2
- S.1.1 Circuit model . . . 2
- S.1.2 Synapse model . . . 3
- S.1.3 Memory patterns . . . 3
- S.1.4 Memory robustness . . . 3
- S.1.5 Noise scaling . . . 4
- S.1.6 Consolidation algorithm . . . 6
- S.1.7 Numerical optimization and evaluation . . . 8
- S.1.8 Theoretical solutions . . . 8
- S.1.9 Simulating wakefulness and sleep . . . 8
- S.1.10 Simulating synaptic intrinsic noise . . . 9
- S.1.11 Control model . . . 9

S.2 Data analysis . . . 9
- S.2.1 Cortical connectivity . . . 9
- S.2.2 Pruning probability . . . 10
- S.2.3 Connection selectivity . . . 10
- S.2.4 Stimulus tuning . . . 10
- S.2.5 Associative memory tests in humans . . . 11
- S.2.6 Environmental enrichment . . . 11
- S.2.7 Sparseness throughout development . . . 11
- S.2.8 Synaptic noise scaling . . . 11
- S.2.9 CV of synapse norms . . . 12

S.3 Extended model analysis . . . 12
- S.3.1 Robustness maximization . . . 13
- S.3.2 Storage maximization . . . 13
- S.3.3 Geometric interpretation . . . 13
- S.3.4 The gating function . . . 15
- S.3.5 The homeostatic function . . . 16
- S.3.6 Related algorithms . . . 17

S.4 Theoretical solutions . . . 18
- S.4.1 Maximal neural noise robustness . . . 18
- S.4.2 Maximal synaptic noise robustness . . . 18
- S.4.3 Maximal pruning . . . 19

S.5 Derivation of sparseness . . . 20

S.6 Extended synaptic noise analysis . . . 20
- S.6.1 Effect of longer sampling intervals . . . 20
- S.6.2 Alternative synaptic noise models . . . 20

S.7 Simulation parameters . . . 22

S.8 Metadata for synaptic imaging . . . 23

S.9 Supplementary figures . . . 24

S.1. Model details

S.1.1. Circuit model. We model a local cortical circuit of pyramidal cells as a recurrent network of N binary neurons. At time t, the output state $s_i(t)$ of each neuron $i = 1, \ldots, N$ is given by

$$s_i(t) = \Theta\big(I_i(t)\big) \tag{S1}$$

where Θ is the Heaviside function and I_i is the total input current, which is the sum of two non-negative current contributions, according to

$$I_i(t) = I_{\text{exc},i}(t) - I_{\text{inh},i}(t)\ . \tag{S2}$$

The first term is the excitatory input, which is determined by the recurrent connectivity and the previous state of the network, as in

$$I_{\text{exc},i}(t) = \sum_{j=1}^{N} w_{ij} s_j(t-1) \tag{S3}$$

where $w_{ij} \geq 0$ denotes the connection strength from neuron j to i. Self-connections are not allowed (i.e., $w_{ii} = 0$).

The second current term, $I_{\text{inh},i}$, is an inhibitory current which is neuron-specific and changes slowly, on a time-scale comparable to that of the excitatory weights (see plasticity rules below).

S.1.2. Synapse model. We consider each synapse to be comprised of $z \in \mathbb{N}$ sub-synaptic components U_{ijk} (also referred to as factors), such that the strength of the connection as a whole can be written as the product

$$w_{ij} = \prod_{k=1}^{z} U_{ijk} \; . \tag{S4}$$

Each component can, for instance, be the area of a post-synaptic scaffold protein, a concentration of membrane receptors, a relative receptor efficacy, or a neurotransmitter release probability. It is therefore possible for each U_{ijk} to represent a separate type of physical quantity, with its own unit of measurement. In order to measure the strength of all components on a common scale, we rewrite each one as

$$U_{ijk} = \bar{U}_k u_{ijk} \tag{S5}$$

where $\bar{U}_k$ is a constant that carries the unit and sets the measurement scale, whereas u_{ijk} is a unit-free measure that represents a relative strength on the same scale for all k (the measurement scale is implicitly defined by the constraint in Eq. S34). The weight can now be written as

$$w_{ij} = \bar{U} \cdot \prod_{k=1}^{z} u_{ijk} \tag{S6}$$

where the proportionality constant $\bar{U} = \prod_k \bar{U}_k$ is the same across all weights and neurons. This constant only changes the length of all weight vectors, and can therefore be set to $\bar{U} = 1$ without any loss of generality.

S.1.3. Memory patterns. Each memory pattern consists of a random binary vector ξ_i^μ, where $i = 1, \ldots, N$ is the neuron index, while $\mu = 1, \ldots, M$ is the index of the pattern. Each element ξ_i^μ is independently assigned one with probability $0 < f < 0.5$ and zero with probability $1 - f$. The parameter f is the average fraction of active neurons in each pattern, and is therefore referred to as the level of pattern activity.

We deviate slightly from this model when simulating wakefulness and sleep. In this case, each pattern contains *exactly* fN ones and $(1-f)N$ zeros, to facilitate the few-shot learning procedure in wakefulness.

S.1.4. Memory robustness. The robustness of a single pattern μ with respect to neuron i is quantified with the signal-to-noise ratio of the input current at the moment of recall. We generally write this as

$$\mathrm{SNR}_i^\mu = \frac{\mathrm{Signal}_i^\mu}{\mathrm{Noise}_i^\mu} \tag{S7}$$

where both the signal and noise are pattern- and neuron-specific. As an approximation, we replace the noise with the strictly neuron-specific variant, by averaging across all patterns and obtaining

$$\mathrm{Noise}_i^\mu \approx \mathbb{E}_\mu[\mathrm{Noise}_i^\mu] =: \mathrm{Noise}_i \; . \tag{S8}$$

Expressions for this quantity can be found in the next section. The signal is calculated as the signed input current deflection during noise-free recall, that is

$$\begin{aligned} \mathrm{Signal}_i^\mu &= (\textstyle\sum_j^N w_{ij}\xi_j^\mu - I_{\mathrm{inh},i})(2\xi_i^\mu - 1) \\ &\overset{!}{=} |\textstyle\sum_j^N w_{ij}\xi_j^\mu - I_{\mathrm{inh},i}| \\ &= |I_i^\mu| \end{aligned} \tag{S9}$$

where the highlighted equality holds under the assumption that all pattern have been encoded error-free. This gives us the approximation

$$\mathrm{SNR}_i^\mu \approx \frac{|I_i^\mu|}{\mathrm{Noise}_i} \; . \tag{S10}$$

We now define the robustness of pattern μ as a whole as the smallest SNR_i^μ over all neurons, meaning

$$\mathrm{SNR}^\mu := \min_i \; \mathrm{SNR}_i^\mu \; . \tag{S11}$$

The robustness for multiple patterns, however, is ill-defined, as the optimization of SNR for one pattern can be incompatible with the storage of another. Therefore, in order to guarantee that no pattern is destabilized and forgotten, we define the total robustness of multiple patterns as the SNR of the weakest pattern, so that

$$\mathrm{SNR} := \min_\mu \min_i \; \mathrm{SNR}_i^\mu \tag{S12}$$

The order of the two minimizations can be switched. This enables us to maximize the total SNR by letting each neuron independently maximize its neuron-specific robustness

$$\mathrm{SNR}_i := \min_\mu \; \mathrm{SNR}_i^\mu \; . \tag{S13}$$

The optimal set of weights and inhibitions are defined as

$$\underset{\substack{w_{i1},\ldots,w_{iN} \\ I_{\mathrm{inh},i}}}{\arg\max} \; \mathrm{SNR}_i \; . \tag{S14}$$

S.1.5. Noise scaling. One can distinguish between a total of three types of noise in the network: background noise, neural noise, and synaptic noise. We define the first two types in the same way as previous theoretical work (1), and then complement the analysis with the third type, which is new.

Background noise Background noise refers to noise that is caused either by biochemical processes inherent to the neurons themselves, or by external inputs that are unrelated to the neural circuit we are observing. As such, we model background noise as a weight-independent, random current contribution δI_i that is added to the total input current, according to

$$\hat{I}_i = \sum_j^N w_{ij} s_j - I_{\text{inh,i}} + \delta I_i \tag{S15}$$

where $\hat{I}_i$ denotes a noisy, stochastic variant of the deterministic input current I_i. Such noise can be made arbitrarily small in relation to the signal, irrespective of the tuning of individual weights, simply by scaling up the excitatory and inhibitory currents. We therefore omit background noise from further analysis.

Neural noise Neural noise corresponds to noise that directly alters the output state of neurons. This is, for example, caused by distorted external stimuli or by transmission failures in afferent connections, that trigger firing when inputs are below threshold, or block firing when inputs are above threshold. We assume that distorted stimuli are generated by the same statistical process as the original patterns, and that they therefore retain the same average level of activity. To create a distorted instance of pattern μ, we flip the original pattern state ξ_i^μ according to

$$0 \mapsto 1 \quad \text{with probability} \quad \tfrac{f_{\text{noise}}}{2(1-f)} \tag{S16}$$

$$1 \mapsto 0 \quad \text{with probability} \quad \tfrac{f_{\text{noise}}}{2f} \tag{S17}$$

and obtain a new pattern $\hat{\xi}_i^\mu$, which, on average, contains $N f_{\text{noise}}$ errors, where f_{noise} is referred to as the noise level. This can be shown by calculating the expected error rate

$$\begin{aligned} \mathbb{E}\left[|\hat{\xi} - \xi|\right] &= \mathbb{P}\left(\hat{\xi}{=}1 \mid \xi{=}0\right) \mathbb{P}\Big(\xi{=}0\Big) + \mathbb{P}\left(\hat{\xi}{=}0 \mid \xi{=}1\right) \mathbb{P}\Big(\xi{=}1\Big) \\ &= \frac{f_{\text{noise}}}{2(1-f)}(1-f) + \frac{f_{\text{noise}}}{2f} f \\ &= f_{\text{noise}} \, . \end{aligned} \tag{S18}$$

The activity level in the distorted pattern, however, remains unchanged, as shown by

$$\begin{aligned} \mathbb{E}\left[\hat{\xi}\right] &= \mathbb{P}\left(\hat{\xi}{=}1\right) \\ &= \mathbb{P}\left(\hat{\xi}{=}1 \mid \xi{=}0\right) \mathbb{P}\Big(\xi{=}0\Big) + \mathbb{P}\left(\hat{\xi}{=}1 \mid \xi{=}1\right) \mathbb{P}\Big(\xi{=}1\Big) \\ &= \frac{f_{\text{noise}}}{2(1-f)}(1-f) + (1 - \frac{f_{\text{noise}}}{2f}) f \\ &= f \, . \end{aligned} \tag{S19}$$

The distorted pattern $\hat{\xi}_i^\mu$ can be compactly described as a random variable

$$\hat{\xi}_i^\mu \sim \text{Bernoulli}\left(\frac{f_{\text{noise}}}{2(1-f)}(1-\xi_i^\mu) + (1 - \frac{f_{\text{noise}}}{2f})\xi_i^\mu \right) \, . \tag{S20}$$

During pattern recall, we initialize each neuron i in the state $\hat{\xi}_i^\mu$, and update the network synchronously. Each neuron receives an input current that, across multiple trials, fluctuates with variance

$$\begin{aligned} \mathbb{V}_{\hat{\xi}_j^\mu}\left[\hat{I}_i\right] &= \mathbb{V}_{\hat{\xi}_j^\mu}\left[\textstyle\sum_j^N w_{ij} \hat{\xi}_j^\mu\right] \\ &= \textstyle\sum_j^N w_{ij}^2 \, \mathbb{V}_{\hat{\xi}_j^\mu}\left[\hat{\xi}_j^\mu\right] \\ &= \textstyle\sum_j^N w_{ij}^2 \Big[\tfrac{f_{\text{noise}}}{2(1-f)} \left(1 - \tfrac{f_{\text{noise}}}{2(1-f)}\right)(1-\xi_j^\mu) \\ &\qquad + \tfrac{f_{\text{noise}}}{2f}\left(1 - \tfrac{f_{\text{noise}}}{2f}\right)\xi_j^\mu \Big] \, . \end{aligned} \tag{S21}$$

We average this quantity over all stored patterns and obtain

$$\mathbb{E}_\mu\left[\mathbb{V}_{\hat{\xi}_j^\mu}\left[\hat{I}_i\right]\right] = \textstyle\sum_j^N w_{ij}^2 \left[f_{\text{noise}} + \tfrac{f_{\text{noise}}^2}{4}\left(\tfrac{1-2f}{f(1-f)}\right)\right] \, . \tag{S22}$$

We finally estimate the noise fluctuation size as the averaged standard deviation

$$\text{Neural noise}_i = \sqrt{\textstyle\sum_j^N w_{ij}^2 \left[f_{\text{noise}} + \tfrac{f_{\text{noise}}^2}{4}\left(\tfrac{1-2f}{f(1-f)}\right)\right]} \, . \tag{S23}$$

When evaluating the empirical robustness to neural noise, we report the results in terms of the relative noise level $f_{\text{noise}}/f \leq 2$.

Synaptic noise Synaptic noise represents intrinsic fluctuations in the most volatile constituents of the synaptic anatomy. We model this noise by adding a small, i.i.d. random perturbation δu to one of the sub-synaptic components in all observable (non-pruned) connections. The perturbation is drawn from a normal distribution $\mathcal{N}(0, \sigma^2_{\text{noise}})$. For simplicity, we assume that all sub-synaptic components are equal, so that $u_{ij1} = \ldots = u_{ijz} = u_{ij}$ (we later show that this assumption is justified in consolidated networks).

First, we note that a perturbation δu in one of the sub-synaptic components causes the whole weight to be perturbed by a magnitude δw that, when averaged over noise samples, is

$$\begin{aligned} \mathbb{E}_{\delta u}[|\delta w|] = \mathbb{E}_{\delta u}[|\hat{w} - w|] &= \mathbb{E}_{\delta u}[|\hat{u} \cdot u^{z-1} - u^z|] \\ &= \mathbb{E}_{\delta u}[|(u + \delta u) \cdot u^{z-1} - u^z|] \\ &= \mathbb{E}_{\delta u}[|\delta u \cdot u^{z-1}|] \\ &= \mathbb{E}_{\delta u}[|\delta u|] \cdot u^{z-1} \\ &= \sigma_{\text{noise}} \sqrt{\tfrac{2}{\pi}} \cdot w^{1-1/z} \end{aligned} \tag{S24}$$

where we use the circumflex to, again, signify stochastically perturbed quantities. We test the impact of this noise on memory recall by first perturbing all connections in the network, then initializing each neuron i in a pattern ξ_i^μ, and finally updating the network synchronously. We separate the robustness analysis into two cases:

$\boxed{z = 1}$ After the first update, each neuron receives an input current that, across many trials, fluctuates with variance

$$\begin{aligned} \mathbb{V}_{\delta u}\left[\hat{I}_i\right] &= \mathbb{V}_{\delta u}\left[\textstyle\sum_j^N (u_{ij} + \delta u_{ij}) \xi_j^\mu\right] \\ &= \textstyle\sum_j^N {\xi_j^\mu}^2 \, \mathbb{V}_{\delta u}\left[\delta u_{ij}\right] \\ &= \textstyle\sum_{j: w_{ij} > 0}^N {\xi_j^\mu}^2 \sigma^2_{\text{noise}} \,. \end{aligned} \tag{S25}$$

We use $\mathbb{E}_\mu[{\xi_j^\mu}^2] = \mathbb{E}_\mu[\xi_j^\mu] = f$ to average the variance across all stored patterns and obtain

$$\begin{aligned} \mathbb{E}_\mu\left[\mathbb{V}_{\delta u}\left[\hat{I}_i\right]\right] &= \textstyle\sum_{j: w_{ij} > 0}^N f \sigma^2_{\text{noise}} \\ &= N f_{w_i} f \sigma^2_{\text{noise}} \end{aligned} \tag{S26}$$

where f_{w_i} denotes the fraction of weights that impinge on neuron i and have not been pruned, that is

$$f_{w_i} = \frac{1}{N} \sum_j^N \mathbb{1}_{\{w_{ij} > 0\}} \,. \tag{S27}$$

This yields the averaged standard deviation

$$\text{Synaptic noise}_i^{(z=1)} = \sigma_{\text{noise}} \sqrt{N f_{w_i} f} \,. \tag{S28}$$

$\boxed{z > 1}$ For multi-factor synapses, the variance of trial-to-trial input fluctuations is given by

$$\begin{aligned} \mathbb{V}_{\delta u}\left[\hat{I}_i\right] &= \mathbb{V}_{\delta u}\left[\textstyle\sum_j^N (u_{ij} + \delta u_{ij}) u_{ij}^{z-1} \xi_j^\mu\right] \\ &= \textstyle\sum_j^N u_{ij}^{2z-2} {\xi_j^\mu}^2 \, \mathbb{V}_{\delta u}\left[\delta u_{ij}\right] \\ &= \textstyle\sum_j^N w_{ij}^{2-2/z} {\xi_j^\mu}^2 \sigma^2_{\text{noise}} \end{aligned} \tag{S29}$$

where we insert $u_{ij} = w_{ij}^{1/z}$ to produce the last expression. The average variance across all stored patterns is now

$$\mathbb{E}_\mu\left[\mathbb{V}_{\delta u}\left[\hat{I}_i\right]\right] = \textstyle\sum_j^N w_{ij}^{2-2/z} f \sigma^2_{\text{noise}} \tag{S30}$$

which yields the averaged standard deviation

$$\text{Synaptic noise}_i^{(z>1)} = \sigma_{\text{noise}} \sqrt{\textstyle\sum_j^N f w_{ij}^{2-2/z}} \,. \tag{S31}$$

We compute the bias produced by synaptic noise as the difference between the average input current in the noisy and noise-free condition. This is zero for any z, as shown by

$$\begin{aligned} \text{Bias}_i &= \mathbb{E}_{\mu,\delta u}[\hat{I}_i] - \mathbb{E}_\mu\left[I_i\right] \\ &= \mathbb{E}_{\mu,\delta u}\left[\textstyle\sum_j^N (u_{ij} + \delta u_{ij}) u_{ij}^{z-1} \xi_j^\mu\right] - \mathbb{E}_\mu\left[\textstyle\sum_j^N w_{ij} \xi_j^\mu\right] \\ &= \mathbb{E}_{\mu,\delta u}\left[\textstyle\sum_j^N \delta u_{ij} u_{ij}^{z-1} \xi_j^\mu\right] \\ &= 0 \,. \end{aligned} \tag{S32}$$

In order to compare the robustness to synaptic noise empirically across different network models, we always scale the noise level σ_{noise} relative to the mean of all observable synaptic components $\langle u_i \rangle_{\text{obs}}$ in each neuron i, where

$$\langle u_i \rangle_{\text{obs}} = \frac{\sum_{j,k} u_{ijk}}{\sum_{j,k} \mathbb{1}_{\{u_{ijk}>0\}}} \,. \tag{S33}$$

In practice, this is done by scaling all afferent connections so that $\langle u_i \rangle_{\text{obs}} = 0.1$ prior to testing.

S.1.6. Consolidation algorithm. We define the process of consolidation as the maximization of the neuron-specific robustness SNR_i in each neuron i. We achieve this by maximizing the signal while keeping the noise fixed. The quantities N, M, f, f_{noise}, and σ_{noise} are intrinsic to the circuit, and therefore considered constant. Crucially, we formulate the maximization in terms of sub-synaptic components u, to ensure that the resulting algorithm employs multiplicative homeostatic scaling. Thus, we formally define consolidation as the optimization

$$\underset{\substack{u_{i11},\ldots,u_{iNz} \\ I_{\text{inh},i}}}{\arg\max} \; \min_{\mu} |I_i^\mu| \quad \text{s.t.} \quad \sum_{j,k} u_{ijk}^2 = \bar{u} \tag{S34}$$

where $\bar{u}$ is an arbitrary constant. To make the problem more tractable, we denote the index of the weakest pattern as $\mu_i^* = \arg\min_\mu |I_i^\mu|$ and rewrite the objective as

$$\min_\mu |I_i^\mu| = |I_i^{\mu_i^*}| = \sum_\mu \mathbb{1}_{\{\mu=\mu_i^*\}} \cdot |I_i^\mu| \,. \tag{S35}$$

We solve this numerically using projected gradient descent. The derivative of the objective with respect to a weight component u_{ijk} is

$$\frac{\partial}{\partial u_{ijk}} |I_i^{\mu_i^*}| = \sum_\mu \mathbb{1}_{\{\mu=\mu_i^*\}} \cdot \text{sgn}(I_i^\mu) \xi_j^\mu \prod_{k' \neq k} u_{ijk'} \,. \tag{S36}$$

To avoid having to determine μ_i^* in practice, we replace the indicator function with its soft approximation, defined as

$$\mathbb{1}_{\{\mu=\mu_i^*\}} \approx \text{Softmin}(|I_i^\mu|) = \frac{e^{-\beta_i |I_i^\mu|}}{\sum_\mu e^{-\beta_i |I_i^\mu|}} \tag{S37}$$

where β_i is a sharpness parameter (also referred to as an inverse temperature). This approximation becomes an exact equality in the limit $\beta_i \to \infty$. Under the assumption that none of the sub-synaptic components are exactly zero, we further simplify the notation by writing

$$\prod_{k' \neq k} u_{ijk'} = \frac{w_{ij}}{u_{ijk}} \,. \tag{S38}$$

We now define a neuron-specific plasticity gating function g_i as

$$g_i(I_i^\mu) := \text{sgn}(I_i^\mu) e^{-\beta_i |I_i^\mu|} \tag{S39}$$

where we use a neuron-specific sharpness parameter β_i, given by

$$\beta_i := \frac{\bar{\beta}}{\frac{1}{M} \sum_\mu |I_i^\mu|} \tag{S40}$$

which adjusts the width of the gating function according to the average input current ($\bar{\beta} > 0$ is a constant). See S.3.4 *The gating function* for more details. We also use a neuron-specific learning rate

$$G_i := \frac{\bar{g}}{\sum_\mu |g_i(I_i^\mu)|} \tag{S41}$$

that is computed at the end of each replay cycle to ensure that the total sum of expressed plasticity stays roughly at a constant level ($\bar{g}$ is a constant). We insert Eqs. S37–S41 in Eq. S36 and obtain the synaptic update rule

$$\begin{aligned} \Delta u_{ijk} &= \bar{g} \cdot \tfrac{\partial}{\partial u_{ijk}} |I_i^{\mu_i^*}| \\ &\approx \bar{g} \cdot \textstyle\sum_\mu \text{Softmin}(|I_i^\mu|) \, \text{sgn}(I_i^\mu) \xi_j^\mu \tfrac{w_{ij}}{u_{ijk}} \\ &= G_i \cdot \textstyle\sum_\mu g(I_i^\mu) \xi_j^\mu \tfrac{w_{ij}}{u_{ijk}} \,. \end{aligned} \tag{S42}$$

Analogously, the update for the inhibitory current becomes

$$\begin{aligned} \Delta I_{\text{inh},i} &= \bar{g} \cdot \tfrac{\partial}{\partial I_{\text{inh},i}} |I_i^{\mu_i^*}| \\ &\approx -\bar{g} \cdot \textstyle\sum_\mu \text{Softmin}(|I_i^\mu|) \, \text{sgn}(I_i^\mu) \\ &= -G_i \cdot \textstyle\sum_\mu g(I_i^\mu) \,. \end{aligned} \tag{S43}$$

We summarize the discrete-time consolidation process in Algorithm 1. When $z = 1$, and with specific choices of the g-function, this algorithm reduces to the well-known gradient ascent, normalized gradient ascent (2), and batch perceptron algorithm (3) (section S.3.6 *Related algorithms*).

At optimal weight configuration, all sub-synaptic components within the same weight adopt the same value, so that

$$u_{ij1} = u_{ij2} = \ldots = u_{ijz} =: u_{ij} \; . \qquad \text{[S44]}$$

This is, in fact, a requirement that the solution *must* satisfy (see section S.3). The homeostatic constraint in Eq. S34 is now reduced to

$$\sum_{j,k} u_{ijk}^2 = \sum_j z u_{ij}^2 = \sum_j z w_{ij}^{2/z} = \bar{u} \qquad \text{[S45]}$$

which means that the whole optimization problem is equivalent to an $L_{2/z}$-regularized maximization, according to

$$\underset{\substack{w_{i1},\ldots,w_{iN} \\ I_{\mathrm{inh},i}}}{\arg\max} \; \min_\mu |I_i^\mu| \quad \text{s.t.} \quad \textstyle\sum_j w_{ij}^{2/z} = \bar{w} \qquad \text{[S46]}$$

where $\bar{w} = \bar{u}/z$.

Algorithm 1 Self-supervised consolidation in an attractor network

---

Apply to all neurons $i = 1, 2, \ldots, N$ **in parallel**:
- Initialize: $\bar{g}$, $\bar{\beta}$, $\beta_i = \bar{\beta}/(\frac{1}{M}\sum_\mu |I_i^\mu|)$
- **for replay cycle** $t = 1, 2, \ldots$ **do** ▷ loop over replay cycles
  - *Part (i): Plasticity induction*
  - $g_i^{(\mathrm{sum})}$; $I_i^{(\mathrm{sum})}$; m; $\Delta I_{\mathrm{inh},i}$; $\Delta u_{i\ldots} \leftarrow 0$ ▷ reset integrators
  - **for pattern** $\mu = 1, 2, \ldots, M$ **do** ▷ replay patterns
    - $s_i \leftarrow \xi_i^\mu$ ▷ cue pattern
    - $I_i^\mu \leftarrow \sum_j^N w_{ij} s_j - I_{\mathrm{inh},i}$ ▷ update network
    - $\forall j, k : \Delta u_{ijk} \leftarrow \Delta u_{ijk} + g_i(I_i^\mu) s_j \frac{w_{ij}}{u_{ijk}}$ ▷ accumulate plasticity signals
    - $\Delta I_{\mathrm{inh},i} \leftarrow \Delta I_{\mathrm{inh},i} + g_i(I_i^\mu)$
    - $I_i^{(\mathrm{sum})} \leftarrow I_i^{(\mathrm{sum})} + |I_i^\mu|$ ▷ integrate current
    - $g_i^{(\mathrm{sum})} \leftarrow g_i^{(\mathrm{sum})} + |g_i(I_i^\mu)|$ ▷ integrate gating signal
    - $m \leftarrow m + 1$ ▷ integrate pattern counter
  - *Part (ii): Plasticity expression*
  - $G_i \leftarrow \bar{g}/g_i^{(\mathrm{sum})}$ ▷ adjust learning rate
  - $\beta_i \leftarrow \bar{\beta} m / I_i^{(\mathrm{sum})}$ ▷ adjust gating window
  - $u_{ijk} \leftarrow [u_{ijk} + G_i \Delta u_{ijk}]_+$ ▷ express plasticity signal
  - $u_{ijk} \leftarrow u_{ijk}\sqrt{\bar{u}/\sum_{j,k} u_{ijk}^2}$ ▷ homeostatic scaling
  - $w_{ij} \leftarrow \prod_k^z u_{ijk}$ ▷ compute weight
  - $I_{\mathrm{inh},i} \leftarrow I_{\mathrm{inh},i} - G_i \Delta I_{\mathrm{inh},i}$ ▷ update inhibition

---

Continuous time To study the dynamics of the weights in continuous time, we formulate the optimization in Eq. S34 as the penalized objective function

$$\mathcal{Q} = -H\big(\textstyle\sum_{j,k} u_{ijk}^2 \, ; \, \bar{u}\big) + |I_i^{\mu_i^*}| \qquad \text{[S47]}$$

where $H(x; \bar{x})$ is a homeostatic penalty function that is zero only when $x = \bar{x}$ and increases monotonically everywhere else. The gradient is

$$\begin{aligned} \frac{\partial \mathcal{Q}}{\partial u_{ijk}} &= -H'\big(\textstyle\sum_{j,k} u_{ijk}^2 \, ; \, \bar{u}\big) \cdot \frac{\partial}{\partial u_{ijk}} \sum_{j,k} u_{ijk}^2 \quad + \quad \frac{\partial}{\partial u_{ijk}} |I_i^{\mu_i^*}| \\ &= 2h\big(\textstyle\sum_{j,k} u_{ijk}^2 \, ; \, \bar{u}\big) u_{ijk} \quad + \quad \frac{\partial}{\partial u_{ijk}} |I_i^{\mu_i^*}| \end{aligned} \qquad \text{[S48]}$$

where $h := -H'$. We simplify the first term with the requirement in Eq. S45, and approximate the second term, as before, using Eqs. S37–S41. Applying gradient ascent in the limit of infinitesimal learning rate gives us the gradient flow

$$\frac{\mathrm{d}u_{ij}}{\mathrm{d}t} \propto \Big[h\big(\textstyle\sum_j z u_{ij}^2 \, ; \, \bar{u}\big) + G_i \sum_\mu g(I_i^\mu) \xi_j^\mu u_{ij}^{z-2}\Big] \cdot u_{ij} \qquad \text{[S49]}$$

where a change of variables back to w_{ij} yields

$$\frac{\mathrm{d}w_{ij}}{\mathrm{d}t} \propto \Big[h\big(\textstyle\sum_j w_{ij}^{2/z} \, ; \, \bar{w}\big) + G_i \sum_\mu g(I_i^\mu) \xi_j^\mu w_{ij}^{1-2/z}\Big] \cdot w_{ij} \; . \qquad \text{[S50]}$$

Note that both differential equations become multiplicative if, and only if, $z = 2$.

S.1.7. Numerical optimization and evaluation.

Initialization All sub-synaptic components are initialized by randomly sampling from the uniform distribution $\mathcal{U}(0.7u_0, 1.3u_0)$, where $u_0 > 0$ is an arbitrary constant. This ensures that the initial u-distribution is strictly positive and has a width that is 60% of the mean, regardless of scaling. Additional parameter values can be found in Supplementary Table S1.

In order to encode all patterns as attractors, prior to consolidation, the network is first trained using the batch perceptron algorithm (3) until all patterns can be recalled without error, where we define the recall error as the fraction of incorrect neurons after one synchronous state update. We compute this as

$$E = \frac{1}{NM} \sum_i^N \sum_\mu^M |s_i^\mu - \xi_i^\mu| \quad \text{[S51]}$$

where

$$s_i^\mu = \Theta\big(\sum_j^N w_{ij}\xi_j^\mu - I_{\mathrm{inh},i} \big) \ . \quad \text{[S52]}$$

Once $E = 0$ is reached, the network is consolidated according to Algorithm 1.

Convergence During the course of consolidation, we monitor the performance of the network using the average SNR_i, average weight density f_{w_i}, and error. The first two are calculated as

$$\langle \mathrm{SNR} \rangle_i = \frac{1}{N} \sum_i \mathrm{SNR}_i \quad \text{[S53]}$$

and

$$\langle f_w \rangle_i = \frac{1}{N} \sum_i f_{w_i} \quad \text{[S54]}$$

where the neuron-specific weight density f_{w_i} is estimated, in practice, using

$$f_{w_i} = \frac{1}{N} \sum_j \Theta(w_{ij} - w_\theta) \quad \text{[S55]}$$

where w_θ is the threshold at which a weight is considered pruned. Once this threshold is reached, the weight is fixed and remains pruned for the remainder of the simulation. In all simulations, we use $w_\theta = 10^{-10}$ (see Suppl. Fig. S1). Weights are also clipped at zero to ensure non-negativity. We consider the consolidation to have converged once $\langle \mathrm{SNR} \rangle_i$ and $\langle f_w \rangle_i$ change by less than 10^{-4} over 10^4 replay cycles, while the error still is at $E = 0$.

Empirical robustness After optimization, we empirically evaluate the robustness of the network by initializing it in each pattern μ together with either neural noise or synaptic noise. We then update the network 50 times and determine if the end state is close to the original pattern using the criterion that the error must satisfy $E < 0.2f$. We perform this test 20 times per pattern, with independent noise samples in each trial. We refer to the average fraction of patterns that can be recalled at each noise level as the recall ratio RR, and we define the empirical robustness as the noise level at which RR falls below 50% (Suppl. Fig. S2).

S.1.8. Theoretical solutions. The theoretical solutions in Figure 1 are adapted from previously published work, primarily references 4–7. For more details, see section S.4 *Theoretical solutions*.

S.1.9. Simulating wakefulness and sleep. We model fast, wakeful learning using a few-shot plasticity rule inspired by Tsodyks and Feigel'man (8), gated by a novelty signal. In each replay cycle, every pattern is presented in random order to the network. This means that the network is initialized in a pattern μ and thereafter updated once. If the subsequent state of the network displays an activity level that differs from f, an additional inhibitory current $I_{\mathrm{inh}}^{(\mathrm{glob})}$ is triggered to regain the desired activity. This indicates that the pattern does not yet form an error-free attractor. The result is registered by the novelty signal $\bar{g}_{\mathrm{new}}$ according to

$$\bar{g}_{\mathrm{new}} = |I_{\mathrm{inh}}^{(\mathrm{glob})}| \quad \text{[S56]}$$

and the network is initialized once again in pattern μ and updated according to

$$\begin{aligned} \Delta u_{ij1} &= \bar{g}_{\mathrm{new}} \bar{g}_{\mathrm{wake}} (s_i - f)(s_j - f) u_{ij2} \\ \Delta u_{ij2} &= 0 \end{aligned} \quad \text{[S57]}$$

without any homeostatic scaling. After each update, weights are clipped at zero to ensure non-negativity. Inhibition is then adjusted to balance excitatory input according to

$$I_{\mathrm{inh},i} = \mathbb{E}_{\mathrm{exc},i} + \sqrt{2fN\,\mathbb{V}_{\mathrm{exc},i}}\,\mathrm{erfc}^{-1}(2f) \quad \text{[S58]}$$

where $\mathbb{E}_{\mathrm{exc}}$ and $\mathbb{V}_{\mathrm{exc}}$ is shorthand for the mean and variance of the excitatory input current across pattern presentations, calculated as

$$\begin{aligned} \mathbb{E}_{\mathrm{exc}} &= \mathbb{E}_\mu \left[\sum_j w_{ij}\xi_j^\mu \right] = f \sum_j w_{ij} \\ \mathbb{V}_{\mathrm{exc}} &= \mathbb{V}_\mu \left[\sum_j w_{ij}\xi_j^\mu \right] = f(1-f) \sum_j w_{ij}^2 \ . \end{aligned} \quad \text{[S59]}$$

Wakeful learning is repeated until none of the patterns trigger the novelty signal. At this point, sleep commences, and both u_{ij1} and u_{ij2} are allowed to change. Consolidation is now modeled using Algorithm 1. Parameter values can be found in Supplementary Table S2.

S.1.10. Simulating synaptic intrinsic noise. To simulate synaptic noise, we assume that one sub-synaptic component is volatile and changes with a fast time constant (fixed to 1), while all remaining components are more stable and characterized by the time constant $\tau \gg 1$. Each weight is therefore parameterized as

$$w_j = \underbrace{u_{j1}}_{\text{fast}} \cdot \underbrace{u_{j2} \cdots u_{jz}}_{\text{slow}} \qquad \text{[S60]}$$

where $j = 1, \ldots, N$. The fast and slow components are governed by the stochastic dynamical system

$$\begin{cases} \dfrac{\mathrm{d}u_{j1}}{\mathrm{d}t} = \left(1 - \frac{1}{N}\sum_{j,k} u_{jk}^2\right) u_{j1} \; + \; u_0 + \sigma_{\text{noise}} \delta u_{j1} & \text{[S61]} \\ \tau \dfrac{\mathrm{d}u_{jk}}{\mathrm{d}t} = \left(1 - \frac{1}{N}\sum_{j,k} u_{jk}^2\right) u_{jk} \; + \; (u_{j1} - u_{jk}) \; , \quad k = 2, \ldots, z & \text{[S62]} \end{cases}$$

where $\delta u_{j1} \sim \mathcal{N}(0,1)$, u_0 is a bias, and σ_{noise} scales the amplitude of the noise fluctuations. All components are initialized at $u_{jk} = 1$ and simulated with step size $\mathrm{d}t = 0.005$ for a total time of $T_{\text{sim}} = 10^3$, with a sampling time of $T_{\text{sample}} = 1$. The analysis in Figures 5 and 6 is performed using the last 144 samples (which corresponds to approximately 24 h if the time unit is assumed to be in the order of 10 min). Additional parameter values can be found in Supplementary Table S4.

S.1.11. Control model. In Figure 4, we compare our model with a control model that has been used in past publications to train attractor networks to achieve optimal storage (7, 9–11). The latter approach is based on the assumption that cortical circuits store patterns with an SNR that is inherently fixed by the plasticity model. Early in development, the storage of new stimuli increases the load of the circuit (as long as $\alpha < \alpha_c$), until critical capacity is reached ($\alpha = \alpha_c$), at which point the circuit enters a steady state where additional storage of new patterns is counterbalanced by forgetting old ones (12). Stated mathematically, this type of consolidation maximizes the storage of the network at a fixed SNR, by solving

$$\underset{w_{i1},\ldots,w_{iN}}{\arg\max} \; M \qquad \text{s. t.} \quad \begin{matrix} \min_{\mu} |I_i^{\mu}| = I_0 \\ I_{\text{inh},i} = I_{\text{inh}} \end{matrix} \qquad \text{[S63]}$$

where $I_0, I_{\text{inh}} > 0$ are constants, and no further reparameterization of the weights is used. The first condition ensures that the signal is fixed, while the second condition imposes a constant inhibition. In ref. 9, it is shown that the solution to Eq. S63 satisfies

$$\textstyle\sum_j w_{ij} = I_{\text{inh}}/f + \mathcal{O}(1/\sqrt{N}) \qquad \text{[S64]}$$

which means that the sum of the weights is constant, up a to correction term that vanishes as $N \to \infty$. If both the signal and the summed weights are constant, then the SNR with respect to $q = 1$ is also constant, which we write as

$$\text{SNR}_i(q=1) = \frac{\text{Signal}_i}{\text{Noise}_i(q=1)} = \frac{I_0}{\sqrt{\sum_j w_{ij}}} = \text{const.} \qquad \text{[S65]}$$

We emphasize that this differs from our consolidation model, where we instead maximize the SNR for a fixed storage load M (see Eq. S34). Borrowing the notation from ref. 9, Eq. S65 is equivalent to a fixed robustness parameter

$$\rho_i = \frac{I_0}{\sum_j w_{ij}} \sqrt{\frac{N}{f(1-f)}} \; . \qquad \text{[S66]}$$

We train the control model using a variant of the perceptron algorithm, whereby we present each pattern μ to the network and compute $|I_i^{\mu}|$ for every neuron i. The weakest pattern in each cycle is tagged with index $\mu_i^* = \arg\min_{\mu} |I_i^{\mu}|$ and used to calculate the robustness ρ_i. The neuron's weights are now updated according to

$$\Delta w_{ij} = \begin{cases} \bar{g}(2\xi_i^{\mu_i^*} - 1)\xi_j^{\mu_i^*} & \text{if} \quad \rho_i < \rho_0 \\ 0 & \text{if} \quad \rho_i \geq \rho_0 \end{cases} \qquad \text{[S67]}$$

where ρ_0 is the robustness threshold. The process is repeated until at least 99% of all neurons satisfy $\rho_i \geq \rho_0$, at which point the optimization stops. Parameter values can be found in Supplementary Table S3.

S.2. Data analysis

S.2.1. Cortical connectivity. The experimental data on connection probability among cortical excitatory cells is part of a publicly available compilation of 124 datasets that were included in a meta-analysis published by Zhang et al. (7). We assign each dataset a weight β_i according to the number of evaluated potential connections n_{conn}, so that

$$\beta_i = \frac{n_{\text{conn}}^{(i)}}{\sum_i^{n_{\text{sets}}} n_{\text{conn}}^{(i)}} \; . \qquad \text{[S68]}$$

The weighted mean (wM) and weighted standard error (wSE) of the connection probability P_{conn} is estimated using

$$\text{wM} = \sum_i^{n_{\text{sets}}} \beta_i P_{\text{conn}}^{(i)} \tag{S69}$$

$$\text{wSE} = \sqrt{\sum_i^{n_{\text{sets}}} \beta_i \left(P_{\text{conn}}^{(i)} - \text{wM}\right)^2}\ . \tag{S70}$$

S.2.2. Pruning probability. To analyze the properties of synaptic pruning, we utilize the dataset published by Loewenstein et al. (13). This consists of dendritic spine volume measurements conducted across six sessions, separated by a sampling interval of $\Delta t = 4\,\text{days}$ (see Table S7 for details). We separate spines into three categories: (i) Spines that are first observed sometime between sessions 2 and 6 are defined as "young". Spines observed in session 1 have an unknown age, and are therefore left out. (ii) Spines that disappear at any time between sessions 1 and 6 are defined as "pruned". (iii) Spines that can be seen in at least two consecutive sessions are defined as "old".

To estimate the pruning probability, we first log-normalize the data by calculating the z-score in logarithmic space, according to

$$Z(\log x) = \frac{\log x - \mathbb{E}[\log x]}{\sqrt{\mathbb{V}[\log x]}}\ . \tag{S71}$$

We then bin all spine volumes in sessions $1to5$, and compute the ratio between the number of pruned spines and the total number of spines in each bin. Spines in session 6 are omitted, as it is unknown how many of these that are pruned.

We calculate the simulated pruning probability in the same way, by comparing connection weights that are pruned during sleep to all connection weights before sleep.

S.2.3. Connection selectivity. In order to evaluate how network connectivity depends on neural response properties, we use the excitatory input current during pattern recall as a proxy for graded neural activity, and denote this $r_i^\mu := \sum_j w_{ij}\xi_j^\mu$. We use this to calculate the neural response correlation between two neurons i and j as

$$C_{ij} = \frac{\mathbb{E}_\mu[r_i^\mu r_j^\mu] - \mathbb{E}_\mu[r_i^\mu]\,\mathbb{E}_\mu[r_j^\mu]}{\sqrt{\mathbb{V}_\mu[r_i^\mu]\,\mathbb{V}_\mu[r_j^\mu]}}\ . \tag{S72}$$

To estimate the connectivity and connection strength as a function of response correlation, we bin all neuron pairs according to C_{ij} and thereafter compute the connection probability and average weight in each bin.

We compare our simulations with the experimental data published by Cossell et al. (14). This study reports the connectivity among pyramidal cells in layer 2/3 of mouse visual cortex, together with their neural activity and pair-wise correlations during presentations of natural static images. The authors estimate the connectivity and synaptic strength (in terms of excitatory post-synaptic potentials, EPSPs) as a function of pairwise correlations by binning neuron pairs as described above. In order to compare artificial weights with biological synapses, we normalize all weights and all EPSPs with the largest value in each dataset.

S.2.4. Stimulus tuning. We compute the neural response to a familiar (i.e., consolidated) pattern μ using $r_i^\mu := \sum_j w_{ij}\xi_j^\mu$. Analogously, the response to a novel pattern is computed as $\hat{r}_i^\mu := \sum_j w_{ij}\hat{\xi}_j^\mu$, where $\hat{\xi}^\mu$ denotes a previously unseen pattern that is created by randomly shuffling all entries in pattern ξ^μ. In order to produce the tuning curve, we first z-score the response distribution of each neuron relative to its familiar responses, according to

$$Z(r_i^\mu) = \frac{r_i^\mu - \mathbb{E}_\mu[r_i^\mu]}{\sqrt{\mathbb{V}_\mu[r_i^\mu]}} \quad , \quad Z(\hat{r}_i^\mu) = \frac{\hat{r}_i^\mu - \mathbb{E}_\mu[r_i^\mu]}{\sqrt{\mathbb{V}_\mu[r_i^\mu]}}\ . \tag{S73}$$

and we then sort all Z-scored responses and plot them as a function of their rank, ranging from 1 (highest) to 100 (lowest).

The sharpness, or selectivity, of the tuning is quantified with the *sparseness* (15, 16), which is defined in general terms as

$$\text{Sparseness} := \frac{\mathbb{V}_x[r]}{\mathbb{E}_x[r^2]} \tag{S74}$$

where r is a general neural output activity (e.g., firing rate). This is computed either across stimuli ($x = \mu$) or across neurons ($x = i$); the former variant is typically called *lifetime sparseness*, and describes the selectivity of single neurons, whereas the latter is called *population sparseness*, and describes the response to a single stimulus in the entire population (see section S.5 *Derivation of sparseness* for more details).

We compare the simulated results with the data published by Woloszyn and Sheinberg (17). This consists of firing rates measured in putative excitatory neurons in inferior temporal cortex of macaque monkeys during presentation of familiar and novel images of objects. The experimental firing rates are processed in the same way as the modeled neural responses.

S.2.5. Associative memory tests in humans. In order to estimate how the strength of memory encoding changes across wakefulness and sleep, we utilize the behavioral data reported by Fenn and Hambrick (18, 19), and Ashton and Cairney (20). All three studies involve human subjects tasked with memorizing 40 semantically related word pairs, where recall performance is tested before and after a delay of roughly 12 h of wakefulness or sleep. Using signal detection theory (21), we model the memory traces in each subject as random variables that, during encoding, are drawn from a normal distribution $\mathcal{N}(\mu_{\text{enc}}, \sigma^2_{\text{enc}})$, where μ_{enc} represents the general encoding strength and σ_{enc} accounts for the encoding noise that is present during memorization (both parameters are subject-specific). At testing, memories are assumed to be recallable only if their traces surpass a subject-specific threshold μ_{recall}. Accordingly, the SNR of a subject's memory is given by the normalized distance between the encoding strength and the recall threshold, meaning $(\mu_{\text{enc}} - \mu_{\text{recall}})/\sigma_{\text{enc}}$. This is equivalent to the z-score of the recall ratio. We first define the recall ratio as

$$\text{RR} \coloneqq \frac{\text{Number of correctly recalled items}}{\text{Total number of items}} \tag{S75}$$

and then use this to compute the subject-specific SNR as

$$\text{SNR}_{\text{exp}} \coloneqq \Phi^{-1}(\text{RR} + \epsilon) \tag{S76}$$

where Φ is the normal cumulative distribution function and $\epsilon = (1 - 2\text{RR}) \cdot 10^{-16}$ is a small corrective term added to avoid divergence. We calculate the change in SNR over the course of the delay period as

$$\Delta\text{SNR}_{\text{exp}} = \text{SNR}^{(\text{after})}_{\text{exp}} - \text{SNR}^{(\text{before})}_{\text{exp}} \tag{S77}$$

and pool the three datasets. Data points that are further than four standard deviations from the mean are considered outliers and are removed. The data is then fit with the linear model

$$\Delta\text{SNR}_{\text{exp}} = \beta_0 + \beta_1 X_{\text{cond}} + \beta_2 \text{SNR}^{(\text{before})}_{\text{exp}} + \beta_3 X_{\text{cond}} \text{SNR}^{(\text{before})}_{\text{exp}} \tag{S78}$$

where the experimental condition is coded by the categorical variable

$$X_{\text{cond}} = \begin{cases} 0 & \text{if wake} \\ 1 & \text{if sleep} \end{cases} . \tag{S79}$$

We determine if the intercept and slope differ significantly between wake and sleep by conducting a one-sample t-test of β_1 and β_3 relative to zero.

S.2.6. Environmental enrichment. In order to analyze the effects of environmental enrichment on cortical connectivity, we reference the study by Jung and Herms (22). This dataset contains measurements of dendritic spine density in the somatosensory cortex of mice that are kept in either stimulus-enriched or stimulus-impoverished environments from birth to adulthood. We reproduce the density of spines that are classified as "persistent". These are older than 3 weeks and are therefore part of connections that, presumably, have undergone maturation and stabilization.

S.2.7. Sparseness throughout development. To observe how neural activation sparseness changes over long periods of time, we use the experimental data reported by Berkes et al. (23). This consists of spike-time measurements in the visual cortex of awake ferrets that are shown a movie clip at different stages in development, ranging from the period of eye-opening to adulthood. We calculate firing rates by binning the spike data in 10 ms bins. The sparseness is then obtained using Eq. S74.

S.2.8. Synaptic noise scaling. To study the scaling of synaptic noise, we use 20 different datasets of synaptic measurements, acquired in 9 previously published studies (13, 24–31). In general, each datapoint consists of a measurement of a synaptic strength proxy, denoted $\hat{w}$, and the observed change $\Delta\hat{w}$ following a sampling interval Δt. We first separate the data into potentiation ($\Delta\hat{w} > 0$) and depression ($\Delta\hat{w} < 0$) and then calculate the average absolute change $\langle|\Delta\hat{w}|\rangle$ as a function of initial strength by filtering all datapoints in $(\Delta\hat{w}, \hat{w})$-space with a moving average, using window size $n/20$, where n is the sample size.

We obtain an estimate of the scaling exponent as the slope of $\langle|\Delta\hat{w}|\rangle$ in logarithmic space, using linear regression. The mean (M) and standard error (SE) of the exponent is estimated by repeating the averaging and line-fitting with bootstrapping. All datasets are bootstrapped 1000 times, except the datasets in references 24–26, which are bootstrapped 100 times due to their exceptionally large sample size.

To summarize the estimates across datasets, we assign each estimate i a weight β_i according to its inverse variance (squared standard error), as in

$$\beta_i = \text{SE}_i^{-2} \tag{S80}$$

and we use this to calculate the weighted mean (wM) and weighted standard error (wSE) according to

$$\text{wM} = \frac{\sum_i^{n_{\text{sets}}} \beta_i \text{M}_i}{\sum_i^{n_{\text{sets}}} \beta_i} \tag{S81}$$

$$\text{wSE} = \frac{1}{\sqrt{\sum_i^{n_{\text{sets}}} \beta_i}} . \tag{S82}$$

The 99% confidence interval is finally estimated as $[\text{wM} \pm 2.58 \cdot \text{wSE}]$.

S.2.9. CV of synapse norms. We use the artificial synaptic data that is obtained by simulating the dynamical system in Eq. S61, and we analyze only weights that survive until the end of the simulation (i.e., $w_j(T_{\mathrm{sim}}) > 0$). At each sampling time t, we calculate the q-norm of the weights with

$$\|w(t)\|_q = \left(\sum_j w_j(t)^q \right)^{1/q} . \qquad \text{[S83]}$$

We then compute the CV of the q-norm across samples, according to

$$\mathrm{CV}_q = \frac{\sqrt{\mathbb{V}_t \left[\|w(t)\|_q \right]}}{\mathbb{E}_t \left[\|w(t)\|_q \right]} . \qquad \text{[S84]}$$

After repeating this process for a range of q-values, we compute the CV-rank by re-scaling all CV_q-values to lie in the range $[0, 1]$ (from smallest to largest) and we obtain the norm with smallest CV as

$$q_{\min} = \arg\min_q \ \mathrm{CV}_q . \qquad \text{[S85]}$$

We estimate the mean and standard error of the CV-rank and $q_{\min}$ by bootstrapping this procedure 1000 times. In each run, we generate the bootstrapped data by separately re-sampling weights at each time t.

We compare simulated results with experimental data by utilizing the dendritic spine measurements reported by Kaufman et al. (25). The experimental CV-rank and $q_{\min}$ are computed in exactly the same way as for the artificial data (see Suppl. Fig. S12).

S.3. Extended model analysis

In this section, we explain the mathematical foundation of our consolidation algorithm and clarify its relation to other learning algorithms in the literature. As in the main text, we consider a recurrent neural network of N binary neurons with inhibition $I_{\mathrm{inh},i}$ and connection weights $w_{ij} \geq 0$, where $i, j = 1, \ldots, N$. For the sake of brevity, we introduce vector notation and represent all input weights to neuron i with the column vector $\boldsymbol{w}_i = (w_{i1}, \ldots, w_{iN})^\top$ and each pattern μ as $\boldsymbol{\xi}^\mu = (\xi_1^\mu, \ldots, \xi_N^\mu)^\top$. For added simplicity, we omit subscript i. The definition of memory robustness provided in the main text can now be expressed as

$$\mathrm{SNR}(q) \propto \min_\mu \frac{(2\xi^\mu - 1)(\boldsymbol{w}^\top \boldsymbol{\xi}^\mu - I_{\mathrm{inh}})}{\|\boldsymbol{w}\|_q^{q/2}} \qquad \text{[S86]}$$

where the exponent $q > 0$ is chosen depending on the type of noise that is considered. Likewise, the aim of consolidation, as stated in the main text, can be written as the neuron-specific optimization

$$\arg\max_{\boldsymbol{w}, I_{\mathrm{inh}}} \ \min_\mu \ (2\xi^\mu - 1)(\boldsymbol{w}^\top \boldsymbol{\xi}^\mu - I_{\mathrm{inh}}) \quad \text{s. t.} \quad \|\boldsymbol{w}\|_q = \text{const.} \qquad \text{[S87]}$$

This maximizes $\mathrm{SNR}(q)$ subject to a homeostatic constraint placed on $\|\boldsymbol{w}\|_q$. Note, however, that without such a weight constraint, the SNR has no upper limit (for $q < 2$) and can be scaled up indefinitely, at a rate $c^{1-q/2}$, simply by scaling the weights with a constant $c > 1$.

Any solution to Eq. S87 can, in theory, also be found with the optimization

$$\arg\min_{\boldsymbol{w}, I_{\mathrm{inh}}} \|\boldsymbol{w}\|_q \quad \text{s. t.} \quad \min_\mu \ (2\xi^\mu - 1)(\boldsymbol{w}^\top \boldsymbol{\xi}^\mu - I_{\mathrm{inh}}) = \text{const.} \qquad \text{[S88]}$$

but this process would, in practice, be incompatible with a homeostatic process that keeps the weight norm fixed.

In machine learning terms, each neuron can be viewed as a linear classifier that discriminates M random input patterns $\boldsymbol{\xi}^\mu$ according to the output labels ξ^μ. In this context, solving Eq. S87 (or S88) is equivalent to maximizing the classification margin with respect to the L_q-norm, that is

$$K(q) = \min_\mu \frac{(2\xi^\mu - 1)(\boldsymbol{w}^\top \boldsymbol{\xi}^\mu - I_{\mathrm{inh}})}{\|\boldsymbol{w}\|_q} . \qquad \text{[S89]}$$

Given a fixed load $\alpha = M/N$ and pattern sparseness f, the maximum margin K^* that a linear classifier can achieve is determined by a function $K^*(\alpha, f, q)$. This defines the state of *optimal storage*, independently of the scaling of the weight vector $\boldsymbol{w}$. Historically, however, it is more common to rearrange the max-margin function so that the state of optimality instead is defined as the maximum load $\alpha^*(K, f, q)$ that can be attained with a fixed margin K. In this context, the largest possible storage load, at any margin, is referred to as the *critical capacity* α_c, which is given by

$$\alpha_c(f) = \alpha^*(0, f, q) = \max_K \ \alpha^*(K, f, q) . \qquad \text{[S90]}$$

The reason this is independent of q is that a max-margin classifier at saturation ($\alpha^* \to \alpha_c$) has a vanishing margin ($K \to 0$) and therefore no degrees of freedom to move. Hence, only a single solution exists at α_c, regardless of which norm that is used to measure the margin.

As a notational rule, we use an asterisk (*) to denote any variable or function that is at optimal storage. Based on the two formulations of optimality, it is now possible to define the notion of *optimal learning* in two different ways:

S.3.1. Robustness maximization. By considering the state of optimal storage to be determined by $K^*(\alpha, f, q)$, we can define optimal learning as the process of finding the network configuration

$$\underset{\boldsymbol{w}, I_{\text{inh}}}{\arg\max}\ K \quad \text{with} \quad \alpha, f, q = \text{const.} \tag{S91}$$

This is known as the *max-margin classifier* or *support vector machine* (32), and it is equivalent to our definition of consolidation in Eq. S87 (see Suppl. Fig. S14, dark arrows). The advantages of this approach are two-fold: First, it allows the network to flexibly attain maximal robustness and to operate optimally, without risk of catastrophic forgetting, at every storage load that is below critical capacity (i.e., $\alpha < \alpha_c$). Second, it allows for this process to be carried out by an iterative learning rule that includes a homeostatic constraint on the weights.

S.3.2. Storage maximization. If one considers optimal storage to be defined by $\alpha^*(K, f, q)$, it is natural to formulate optimal learning as the process of finding

$$\underset{\boldsymbol{w}, I_{\text{inh}}}{\arg\max}\ \alpha \quad \text{with} \quad K, f, q = \text{const.} \tag{S92}$$

We refer to this as the *storage problem*. The main advantage of this approach is that it, in certain cases, is analytically tractable and allows for the optimal state of the network to be described with closed-form solutions in the mean-field limit $N \to \infty$. Here, we focus on three specific cases:

$\boxed{q = 2}$ This solution is provided by Gardner (4) and is obtained under the weight scaling $w \sim \mathcal{O}(1/\sqrt{N})$ (see Suppl. Fig. S14a, light arrow). We use this to compute the maximum SNR with respect to neural noise. For technical details, see section S.4.1.

$\boxed{q = 1}$ This solution can be found in references 7, 9, 33 and is obtained by keeping I_{inh} fixed and scaling the weights as $w \sim \mathcal{O}(1/N)$ (see Suppl. Fig. S14b, light arrow). We use this solution to compute the maximum SNR with respect to synaptic noise in networks with two-factor synapses. For technical details, consult section S.4.2 *Maximal synaptic noise robustness*.

$\boxed{q \to 0}$ This solution is derived by Bouten et al. (5) by optimally diluting Gardner's solution for $q = 2$. It describes the maximum amount of pruning that can be supported by a network. For technical details, see section S.4.3 *Maximal pruning*.

As a model of memory consolidation, however, the mean-field formulation has a number of disadvantages. Mainly, it is unclear how to translate it to a biologically realistic iterative learning rule, given that the margin K is a constant that has to be fine-tuned, *a priori*, to the particular load α that the network needs to store. One way to avoid this issue is to assume that K always stays fixed and is hard-coded into the learning rule. This is the approach taken in references 1, 9, 10 and in our control model. However, as suggested by our control model simulations (see SI section S.1.11 *Control model*), this type of learning can only achieve optimal storage once the network has accumulated enough patterns to match the fixed margin. Until this point in time, the network operates at suboptimal storage. Moreover, after optimal storage load has been reached, the network must maintain a steady-state of stored patterns, in order to avoid catastrophic forgetting (12).

Finally, we argue that the basic assumption that neural circuits have a fixed robustness and learn to maximize the amount of memories is problematic from an ethological perspective. It implies that the brain does not adapt to environmental cognitive pressures, but instead passively incorporates information as it is encountered, without allowing for further improvement in the encoding.

S.3.3. Geometric interpretation. Instead of solving Eq. S87 directly in terms of $\boldsymbol{w}$, we derive our consolidation algorithm by maximizing the SNR in the space of sub-synaptic u-variables, by solving

$$\underset{\substack{\boldsymbol{u}_1,\ldots,\boldsymbol{u}_z \\ I_{\text{inh}}}}{\arg\max}\ \min_{\mu}\,(2\xi^\mu - 1)(\boldsymbol{w}^\top \boldsymbol{\xi}^\mu - I_{\text{inh}}) \quad \text{s.t.} \quad \textstyle\sum_k \|\boldsymbol{u}_k\|_2^2 = \text{const.} \tag{S93}$$

where the weight vector is composed of the Hadamard product $\boldsymbol{w} = \boldsymbol{u}_1 \odot \ldots \odot \boldsymbol{u}_z$. At optimality, all sub-synaptic vectors align, so that $\boldsymbol{u}_1 = \ldots = \boldsymbol{u}_z = \boldsymbol{u}$. We prove this in the following theorem.

Theorem 1. *Let $\mathcal{Q}$ be homogeneous objective function that obeys $\mathcal{Q}(cw) = c\mathcal{Q}(w)\ \forall c > 0$, where $w \geq 0$, and consider the optimization problem*

$$\underset{u_1,\ldots,u_z}{\arg\max}\ \mathcal{Q}\big(w(u_1,\ldots,u_z)\big) \quad \text{s.t.} \quad \begin{aligned} &\textstyle\sum_k u_k^2 = \bar{u}\,, \\ &u_k \geq 0\,,\ \forall k\,, \end{aligned} \tag{S94}$$

where $\bar{u}$ is a constant and w is parameterized as

$$w(u_1,\ldots,u_z) = \prod_k^z u_k\,. \tag{S95}$$

Then, any local maximum $w^*(u_1^*, \dots, u_z^*)$ must satisfy

$$u_1^* = u_2^* = \dots = u_z^* \,. \qquad \text{[S96]}$$

Proof. Consider a z-dimensional space spanned by the all the u-variables. In the positive orthant, the local maximum $(u_1^*, \dots, u_z^*)$ forms a rectangle together with the coordinate axes. This rectangle has volume w^* and a diagonal of length $\sqrt{\bar{u}}$. Recall that a rectangle with fixed volume minimizes the length of its diagonal only when all sides have equal length (equivalently, a rectangle with fixed diagonal length achieves maximal volume only when all sides are equal). In our case, this implies that for any candidate solution w^* with unequal u^*-variables, a better solution can always be found with the following two steps:

1. Equalize all u^*-variables and generate a new solution v^* with the same volume

$$w^*(v^*, \dots, v^*) = w^*(u_1^*, \dots, u_z^*) \qquad \text{[S97]}$$

   but with a shorter diagonal length

$$\textstyle\sum_k {v^*}^2 = \bar{v} < \sum_k {u_k^*}^2 = \bar{u} \,. \qquad \text{[S98]}$$

2. Rescale v^* with the factor $\bar{c} = \sqrt{\bar{u}/\bar{v}} > 1$ so that $\sum_k (\bar{c}v^*)^2 = \bar{u}$, which satisfies the optimization constraint. The objective function now assumes the value

$$\mathcal{Q}(\bar{c}v^* \cdots \bar{c}v^*) = \mathcal{Q}(\bar{c}^z w^*) = \bar{c}^z \mathcal{Q}(w^*) > \mathcal{Q}(w^*) \,. \qquad \text{[S99]}$$

Thus, the new solution $(\bar{c}v^*, \dots, \bar{c}v^*)$ is superior. This proves that the candidate $(u_1^*, \dots, u_z^*)$ can never be an optimum, and that Eq. S96 therefore is a necessary condition. This argument can be generalized to N dimensions, where w is replaced by the vector $\boldsymbol{w} = (w_1, \dots, w_N)$. In this case, the two-step procedure is applied to each element of the vector separately. ■

This result allows us to rewrite Eq. S93 as

$$\underset{\boldsymbol{u}, I_{\text{inh}}}{\arg\max} \; \min_{\mu} \; (2\xi^\mu - 1)(\boldsymbol{u}^{\odot z^\top} \boldsymbol{\xi}^\mu - I_{\text{inh}}) \quad \text{s.t.} \quad \|\boldsymbol{u}\|_2^2 = \text{const.} \qquad \text{[S100]}$$

which, following a variable change $\boldsymbol{u} = \boldsymbol{w}^{1/z}$, is equivalent to

$$\underset{\boldsymbol{w}, I_{\text{inh}}}{\arg\max} \; \min_{\mu} \; (2\xi^\mu - 1)(\boldsymbol{w}^\top \boldsymbol{\xi}^\mu - I_{\text{inh}}) \quad \text{s.t.} \quad \|\boldsymbol{w}\|_{2/z}^{2/z} = \text{const.} \qquad \text{[S101]}$$

In other words, optimizing the SNR in u-space, using z components per weight, results in a weight vector that solves the original problem in Eq. S87 with exponent $q = 2/z$. In general, this type of regularized optimization yields progressively sparser solutions as z increases (i.e., q decreases). We provide an intuitive explanation for this phenomenon by analyzing the geometry of Eq. S87 from two perspectives: the *neural state space* and the *loss landscape*.

Neural state space We consider a network of three neurons, and we study the two specific cases $z = 1$ and $z = 2$, which are equivalent to solving Eq. S87 with $q = 2$ and $q = 1$, respectively.

$\boxed{q = 2}$ The solution to Eq. S87 is equivalent to a sign-constrained linear classifier at maximum margin $K^*(q = 2)$. In Supplementary Figure S15a, we illustrate this solution in the two-dimensional state space of the afferent neural activity. Here, the optimal weight vector $\boldsymbol{w}^*$ and inhibition I_{inh}^* together define a classification boundary that correctly separates all patterns $\boldsymbol{\xi}^\mu$ and maximizes the Euclidean distance to the nearest items. The boundary is not biased towards any direction, so few entries in the normal vector are pushed to zero, which means that $\boldsymbol{w}^*$ is dense.

$\boxed{q = 1}$ The solution to Eq. S87 is now equivalent to a sign-constrained linear classifier at maximum margin $K^*(q = 1)$. We illustrate the state space representation of this solution in Supplementary Figure S15b. A theorem by Mangasarian (34) tells us that any classifier that maximizes the L_q-margin, where $q \geq 1$, corresponds, in geometric terms, to a boundary that maximizes the $L_{\frac{q}{q-1}}$-distance to the nearest points. Consequently, at $K^*(q = 1)$, the solution is a boundary that maximizes the L_∞-distance to all patterns $\boldsymbol{\xi}^\mu$. This forces the boundary to align with some of the coordinate axes, which zeros the corresponding weights and makes $\boldsymbol{w}^*$ sparse.

Loss landscape We consider, as in the previous section, a neuron with two-dimensional input, and we define, as a simple example, the optimization problem

$$\underset{w_1, w_2 \geq 0}{\arg\max} \; Q(w_1, w_2) \quad \text{s.t.} \quad \|\boldsymbol{w}\|_q = 1 \qquad \text{[S102]}$$

where Q is an objective function given by the paraboloid

$$Q := -1.5(w_1 - 0.55)^2 - (w_2 - 1.4)^2 \,. \qquad \text{[S103]}$$

We present the Q-landscape, together with the constraint $\|\boldsymbol{w}\|_q = 1$, for different q-values, in Supplementary Figure S16a. As q is lowered, the shape of the constraint curve becomes more convex and moves the optimum closer to a sparse solution of type $\boldsymbol{w}^* = (0, w_2^*)$.

We can numerically search for the optimum by performing projected gradient ascent (Suppl. Fig. S16b) according to the iterative algorithm

$$\boldsymbol{w} \leftarrow \mathrm{proj}_{\mathcal{H}}\big(\boldsymbol{w} + \eta \nabla Q\big) \tag{S104}$$

where η is the learning rate and the projection operator is defined as

$$\mathrm{proj}_{\mathcal{H}}(\boldsymbol{w}) := \arg\min_{\boldsymbol{w}' \in \mathcal{H}} \|\boldsymbol{w}' - \boldsymbol{w}\|_2 \tag{S105}$$

where the feasible set is given by $\mathcal{H} := \{\boldsymbol{w}' \geq 0 : \|\boldsymbol{w}'\|_q = 1\}$. We analyze three specific cases of this process:

$\boxed{q=2}$ The projection operator is reduced to a multiplicative scaling, where

$$\mathrm{proj}_{\mathcal{H}}(\boldsymbol{w}) = \boldsymbol{w}/\|\boldsymbol{w}\|_2 \tag{S106}$$

similarly to Oja's rule (35). This is compatible with the kind of homeostatic synaptic plasticity that has been observed experimentally (36), but is irreconcilable with the high degree of sparsity seen in cortical circuits, given that solutions generally are dense (37).

$\boxed{q=1}$ The projection operator is reduced to a subtractive adjustment, applied elementwise according to

$$\mathrm{proj}_{\mathcal{H}}(\boldsymbol{w}) = [\boldsymbol{w} - \theta]_+ \tag{S107}$$

where $[\,\cdot\,]_+$ is the rectified linear function with a threshold θ that must be computed at every iteration, depending on $\boldsymbol{w}$, to satisfy $\|\boldsymbol{w}\|_1 = 1$. The resulting learning rule is now incompatible with biological homeostatic plasticity, but produces solutions with a sparsity comparable to cortical connectivity. Similar methods are used in references 7, 38–40.

$\boxed{0<q<1}$ A closed-form expression for the projection operator is not available, as the shape of the constraint curve requires an anisotropic projection that, in general, adjusts weights by different amounts depending on $\boldsymbol{w}$. This poses a problem both from a modeling perspective and in terms of biological plausibility.

$\boxed{q=0}$ In this case, the projection operator is reduced to the the hard thresholding operation

$$\mathrm{proj}_{\mathcal{H}}(\boldsymbol{w}) = \boldsymbol{w} \odot \Theta(\boldsymbol{w} - \theta) \tag{S108}$$

where Θ is the Heaviside function with a threshold θ that must be computed at every iteration, depending on $\boldsymbol{w}$, to satisfy $\|\boldsymbol{w}\|_0 = 1$. For example, in the two-dimensional case, one chooses $\theta = \min(w_1, w_2)$. This type of projection does not impose any form of homeostatic plasticity, and only prunes weights in order to produce solutions with a pre-defined level of sparsity. A similar method is used in reference 38.

We reconcile the need for multiplicative scaling with sparse solutions by expressing the weights as

$$(w_1, w_2) = (u_1^z, u_2^z) \tag{S109}$$

and solving

$$\arg\max_{u_1, u_2 \geq 0} \; \mathcal{Q}(u_1^z, u_2^z) \quad \text{s.t.} \quad \|\boldsymbol{u}\|_2 = 1\,. \tag{S110}$$

In Supplementary Figure S16c, we plot the reparameterized Q-landscape using, as an example, $z = 3$, together with the constraint curve $\|\boldsymbol{w}\|_{2/3} = 1$. The variable change deforms the landscape in such a way that the constraint curve can be reached with a multiplicative projection, even though the optimal solution remains sparse. The effect is the same for any pair of z and $q = 2/z$.

S.3.4. The gating function. Our derivation of the gating function g originates from the gradient calculation

$$\frac{\partial\,\mathrm{Signal}}{\partial u_{jk}} = \textstyle\sum_\mu \mathbb{1}_{\{\mu=\mu^*\}} \cdot \mathrm{sgn}(I^\mu) \xi_j^\mu \frac{w_j}{u_{jk}} \tag{S111}$$

where we omit index i and use $\mu^* = \arg\min_\mu |I^\mu|$. By replacing the indicator function with the Softmin, as in

$$\textstyle\sum_\mu \mathbb{1}_{\{\mu=\mu^*\}} \cdot \mathrm{sgn}(I^\mu) \approx \frac{e^{-\beta|I^\mu|} \cdot \mathrm{sgn}(I^\mu)}{\sum_\mu e^{-\beta|I^\mu|}} \tag{S112}$$

and then introducing

$$g(I^\mu) = \mathrm{sgn}(I^\mu) e^{-\beta|I^\mu|} \tag{S113}$$

we arrive at the gradient approximation

$$\frac{\partial\,\mathrm{Signal}}{\partial u_{jk}} \approx \frac{\sum_\mu g(I^\mu)\xi_j^\mu \frac{w_j}{u_{jk}}}{\sum_\mu |g(I^\mu)|}\,. \tag{S114}$$

Note that the gating function takes on the shape of a *surrogate gradient* (41). However, in contrast to the typical use-case of surrogate gradients, the performance of our model improves with higher β, as this reduces the discrepancy between the approximate and true gradient (Suppl. Fig. S17). In order to guarantee that the approximate gradient converges to the true gradient in the limit $\beta \to \infty$, the tails of g must decay to zero at a rate that is, at least, faster than a polynomial. We state this in the following theorem.

Theorem 2. *Consider a general Softmin function*

$$\text{Softmin}(x_i) = \frac{g(\beta x_i)}{\sum_i^n g(\beta x_i)} \tag{S115}$$

where $x_i > 0 \; \forall i$, $\beta > 0$ is an inverse temperature, and $g(x)$ is a finite and strictly positive function that decays monotonically to zero as $x \to \infty$. Then,

$$\text{Softmin}(x_i) \to \mathbb{1}_{\{x_i = \min_i x_i\}} \quad \textit{in the limit} \quad \beta \to \infty \tag{S116}$$

iff g decays faster than a polynomial, that is, $g(x) \sim o(x^{-c})$, with $0 < c < \infty$.

Proof. Let x_1 and x_2 denote the smallest and second smallest x_i. The convergence in Eq. S116 is equivalent to

$$\lim_{\beta \to \infty} \log\left(\frac{\text{Softmin}(x_1)}{\text{Softmin}(x_2)}\right) = \log\left(\frac{\mathbb{1}_{\{x_1 = \min_i x_i\}}}{\mathbb{1}_{\{x_2 = \min_i x_i\}}}\right) = \infty \; . \tag{S117}$$

At the same time, we have

$$\log\left(\frac{\text{Softmin}(x_1)}{\text{Softmin}(x_2)}\right) = \log\left(\frac{g(\beta x_1)}{g(\beta x_2)}\right) = \log g(\beta x_1) - \log g(\beta x_2) \; . \tag{S118}$$

We combine Eq. S117 with S118 and obtain

$$\lim_{\beta \to \infty} \frac{\log g(\beta x_1) - \log g(\beta x_2)}{\log(\beta x_1) - \log(\beta x_2)} = \lim_{\beta \to \infty} \frac{\log g(\beta x_1) - \log g(\beta x_2)}{\log(x_1) - \log(x_2)} = -\infty \tag{S119}$$

where the minus sign on the right-hand side is due to $x_1 < x_2$. This condition must hold for any pair of x_1 and x_2, no matter how close they are to each other. In the limit $x_2 \to x_1$, Eq. S119 is equivalent to

$$\lim_{\beta \to \infty} \frac{\mathrm{d} \log(g(\beta x))}{\mathrm{d} \log(\beta x)} = -\infty \tag{S120}$$

which states that the slope of g, in logarithmic space, cannot be bounded, but must tend to $-\infty$. In other words, the tail of g must decay faster than a line in logarithmic space, and, thus, faster than a polynomial in linear space, which means $g(x) \sim o(x^{-c})$. ■

S.3.5. The homeostatic function. As shown in the Methods, the consolidation model with two-factor synapses can be expressed in continuous time with the differential equation

$$\frac{\mathrm{d}w_j}{\mathrm{d}t} \propto \left[h\big(\|\boldsymbol{w}\|_1 \, ; \, \bar{w}\big) + G \sum_\mu g(I^\mu)\xi_j^\mu\right] \cdot w_j \tag{S121}$$

where we omit index i. The dynamics of the homeostatic term is determined by the function h, which is defined as $h = -\frac{\mathrm{d}}{\mathrm{d}x} H(x; \bar{x})$, where H represents a homeostatic penalty function that is zero at $x = \bar{x}$ and increases monotonically everywhere else. This can be viewed as a generalized formulation of homeostatic plasticity, which, depending on the exact shape of H, can be reduced to specific instances of plasticity models that have been proposed in previous work. Consider the following three cases:

Case 1 If we choose the penalty function to be

$$H = \tfrac{1}{2}\big(\bar{w} - \|\boldsymbol{w}\|_1\big)^2 \tag{S122}$$

we obtain the homeostatic function

$$h = \bar{w} - \|\boldsymbol{w}\|_1 \tag{S123}$$

which is identical to the homeostatic scaling rule introduced by Renart et al. (42), albeit expressed in terms of the summed weights instead of input firing rates.

Case 2 If we instead define the penalty as

$$H = \tfrac{1}{2}\left(1 - \frac{\|\boldsymbol{w}\|_1}{\bar{w}}\right)^2 \quad \text{[S124]}$$

we retrieve the homeostatic function

$$h = 1 - \frac{\|\boldsymbol{w}\|_1}{\bar{w}} \quad \text{[S125]}$$

which is the homeostatic rule introduced in by Toyoizumi et al. (43), but expressed in terms of summed weights instead of the input currents.

Case 3 A third alternative for the penalty function is

$$H = x\log(x) - x\ , \quad x = \frac{\|\boldsymbol{w}\|_1}{\bar{w}} \quad \text{[S126]}$$

which yields the homeostatic function

$$h = \log\left(\frac{\bar{w}}{\|\boldsymbol{w}\|_1}\right)\ . \quad \text{[S127]}$$

This type of homeostatic scaling has, to the best of our knowledge, not been proposed previously in the literature.

It is important to note that even though all homeostatic rules regulate the average synaptic weight, they do so by monitoring different quantities. In case 1, the rule depends on a raw deviation from the set-point, while, in case 2, it depends on the percentage of the deviation. In the third case, the homeostatic rule depends only on the ratio of $\|\boldsymbol{w}\|_1$ relative the set-point.

S.3.6. Related algorithms. In this section, we explain the link between the consolidation model and other iterative learning algorithms. The expression for Δu_{ijk} in Eq. S42 can be seen as a generalized weight update rule, which, depending on the value of β_i, can be reduced to three well-known algorithms from the machine learning literature:

$\beta_i = 0$ In this case, our update rule is reduced to the conventional gradient ascent procedure

$$\Delta u_{ijk} \propto \frac{\partial \mathcal{Q}}{\partial u_{ijk}} \quad \text{[S128]}$$

where the objective function $\mathcal{Q}$ is the average signal across all patterns, given by

$$\mathcal{Q} = \tfrac{1}{M}\sum_\mu |I_i^\mu|\ . \quad \text{[S129]}$$

$\beta_i = 1$ This case is equivalent to the *normalized* gradient ascent algorithm (2)

$$\Delta u_{ijk} \propto \frac{1}{\mathcal{Q}}\frac{\partial \mathcal{Q}}{\partial u_{ijk}} \quad \text{[S130]}$$

applied to the exponential objective function

$$\mathcal{Q} = -\sum_\mu e^{-|I_i^\mu|}\ . \quad \text{[S131]}$$

$\beta_i \to \infty$ In this limit, our update rule becomes identical to the *batch perceptron* algorithm (3)

$$\Delta u_{ijk} \propto \mathrm{sgn}(I_i^{\mu_i^*})\xi_i^{\mu_i^*} \quad \text{[S132]}$$

where $\mu_i^* = \arg\min_\mu |I_i^\mu|$.

Both the normalized gradient and the batch perceptron were originally introduced as margin-maximizing learning rules. Indeed, as we demonstrate in Supplementary Figure S17, the performance of our algorithm improves with increasing β_i. At very high β_i, it appears to converge to the batch perceptron, which consistently performs best.

S.4. Theoretical solutions

S.4.1. Maximal neural noise robustness. To calculate the theoretically highest possible SNR with respect to neural noise, we use the solution for the maximum margin $K^*(\alpha, f, q=2)$, which we obtain using the maximum load $\alpha^*(K, f, q=2)$ and solving for K. The maximum load α^* is the solution to Eq. S92 with $q=2$, and is provided by Gardner (4) in the form

$$\alpha^*(K,m) = \frac{1}{2}\left[\tfrac{1}{2}(1+m)\int_{\frac{vm-2K}{\sqrt{1-m^2}}}^{\infty} D(x)\left(\frac{2K-vm}{\sqrt{1-m^2}}+x\right)^2 \mathrm{d}x + \tfrac{1}{2}(1-m)\int_{\frac{-vm-2K}{\sqrt{1-m^2}}}^{\infty} D(x)\left(\frac{2K+vm}{\sqrt{1-m^2}}+x\right)^2 \mathrm{d}x\right]^{-1} \quad \text{[S133]}$$

where v is given by the solution to the equation

$$\tfrac{1}{2}(1+m)\int_{\frac{vm-2K}{\sqrt{1-m^2}}}^{\infty} D(x)\left(\frac{2K-vm}{\sqrt{1-m^2}}+x\right)\mathrm{d}x = \tfrac{1}{2}(1-m)\int_{\frac{-vm-2K}{\sqrt{1-m^2}}}^{\infty} D(x)\left(\frac{2K+vm}{\sqrt{1-m^2}}+x\right)\mathrm{d}x \quad \text{[S134]}$$

and D is the standard normal distribution

$$D(x) = \frac{\exp(-\frac{1}{2}x^2)}{\sqrt{2\pi}} \quad \text{[S135]}$$

and m is the pattern magnetization, which simply reflects the activity level f according to

$$m = 2f - 1\,. \quad \text{[S136]}$$

In the specific case of balanced patterns ($f=0.5$), Eq. S133 is evaluated at $m=0$ and reduced to

$$\alpha^*(K,0) = \frac{1}{2}\left[\int_{-2K}^{\infty} D(x)(2K+x)^2\,\mathrm{d}x\right]^{-1}. \quad \text{[S137]}$$

Note that both α^* and K have been adjusted with a factor $\frac{1}{2}$ relative the original solution by Gardner. This accounts for the fact that we allow only non-negative weights (44) and use pattern-values in $\{0,1\}$, while the original solution was derived for unconstrained weights and patterns in $\{\pm 1\}$. The SNR is now computed as

$$\mathrm{SNR}^* = \frac{\mathrm{Signal}^*}{\mathrm{Neural\ noise}^*} = \frac{K_2^*}{\sqrt{f_{\mathrm{noise}} + \frac{f_{\mathrm{noise}}^2}{4}\left(\frac{1-2f}{f(1-f)}\right)}} \quad \text{[S138]}$$

where K_2^* is shorthand for $K^*(\alpha, f, q=2)$.

S.4.2. Maximal synaptic noise robustness.

Single-factor synapses In the case of $z=1$, synaptic noise depends on the fraction of non-pruned weights f_w (see Methods). In order to compute the highest possible SNR with respect to synaptic noise, it is therefore necessary to derive the fraction of weights that a sign-constrained linear classifier exhibits at K_2^*. We denote this optimal fraction $f_w^*(\alpha, f, q=2)$. At activity level $f=0.5$, this is, in fact, known to be exactly 50%, regardless of storage load (6). Given a weight norm $\|\boldsymbol{w}\|_2$, the optimal signal can, according to Eq. S89, be written as $\mathrm{Signal}^* = K_2^*\|\boldsymbol{w}\|_2$, which gives us the maximal SNR

$$\mathrm{SNR}^*_{(z=1)} = \frac{\mathrm{Signal}^*}{\mathrm{Synaptic\ noise}^*} = \frac{K_2^*\|\boldsymbol{w}\|_2}{\sigma_{\mathrm{noise}}\sqrt{Nff_{w_i}}} = \frac{2K_2^*\|\boldsymbol{w}\|_2}{\sigma_{\mathrm{noise}}\sqrt{N}} \quad \text{[S139]}$$

where the last equality is obtained by inserting $f = f_w^* = 0.5$.

Two-factor synapses In the case of $z = 2$, we can compute the highest possible SNR with respect to synaptic noise using the solution for the maximum margin $K^*(\alpha, f, q = 1)$, which is obtained from the maximum load $\alpha^*(K, f, q = 1)$ after solving for K. The maximum load α^* is the solution to Eq. S92 with $q = 1$. This was first published in references 9, 33. Here, however, we use the solution reported by Zhang et al. (7), which is expressed as

$$\alpha^*(K, f) = \frac{2K^2 N}{\sigma^2(v_- + v_+)^2 f(1-f)} \frac{fF_3(v_-) + (1-f)F_3(v_+)}{(fF_1(v_-) + (1-f)F_1(v_+))^2} \tag{S140}$$

where the variables (x, v_-, v_+, σ) are given by the solution to the system of equations

$$\begin{cases} F_2(x) = \dfrac{\sqrt{2}}{\sigma} \\ F_3(x) = \dfrac{2K^2 N}{\sigma^2(v_- + v_+)^2 f(1-f)} \\ \dfrac{fF_1(v_-) + (1-f)F_1(v_+)}{fF_2(v_-) + (1-f)F_2(v_+)} = \dfrac{-K^2 N}{\sqrt{2}\sigma x(v_- + v_+)f(1-f)} \\ fF_2(v_-) - (1-f)F_2(v_+) = 0 \\ v_- + v_+ > 0 \\ \sigma > 0 \end{cases} \tag{S141}$$

where we use the auxiliary functions

$$\begin{cases} F_1(x) = \frac{1}{2}(1 + \operatorname{erf}(x)) \\ F_2(x) = \frac{1}{\sqrt{\pi}} e^{-x^2} + x(1 + \operatorname{erf}(x)) \\ F_3(x) = F_1(x) + xF_2(x) \ . \end{cases} \tag{S142}$$

This is also used to compute the optimal weight fraction

$$f_w^*(\alpha, f, q = 1) = F_1(x) \ . \tag{S143}$$

The maximal signal at a given weight norm $\|\boldsymbol{w}\|_1$ is now given by $\text{Signal}^* = K_1^* \|\boldsymbol{w}\|_1$ (see Eq. S89), which yields the maximal SNR

$$\text{SNR}^*_{(z=2)} = \frac{\text{Signal}^*}{\text{Synaptic noise}^*} = \frac{K_1^*}{\sigma_{\text{noise}}} \sqrt{\frac{\|\boldsymbol{w}\|_1}{f}} \tag{S144}$$

where K_1^* is shorthand for $K^*(\alpha, f, q = 1)$.

S.4.3. Maximal pruning. In the limit $z \to \infty$, our definition of consolidation is equivalent to a maximization of the L_0-margin, which, according to Eq. S88, can be formulated as a minimization of the number of observable weights $\|\boldsymbol{w}\|_0$ relative the signal. This, in other words, describes the maximum fraction of weights that can be pruned by a neuron, without losing any of the stored patterns. In order to compute this, we turn to the optimal storage definition in Eq. S92 and supplement it with an additional constraint that requires the optimum to have a desired weight fraction f_w. The result is a new storage optimization

$$\underset{\boldsymbol{w}, I_{\text{inh}}}{\arg\max}\ \alpha \quad \text{with} \quad K, f, q, f_w = \text{const.} \tag{S145}$$

whose solution now is described by the maximal storage load $\alpha^*(K, f, q, f_w)$. The value of α^* that is attained at the smallest possible margin, that is $K = 0$, is the critical capacity

$$\alpha_c^*(f, f_w) = \alpha^*(0, f, q, f_w) \ . \tag{S146}$$

Note, again, that this function is independent of q, as the zero-margin solution is the same for all q. The highest degree of pruning is now determined by the lowest possible f_w at a given α_c. We obtain this by solving for f_w in Eq. S146 and write it as the function

$$f_w^*(\alpha_c, f) \ . \tag{S147}$$

In the case of balanced patterns, $f = 0.5$, a derivation of $\alpha_c^*(f, f_w)$ can be found in the work by Bouten et al. (5). The result is

$$\alpha_c^*(f_w) = 2f_w + \frac{2}{\sqrt{\pi}} \operatorname{erfc}^{-1}(2f_w) \cdot \exp\left[-\operatorname{erfc}^{-1}(2f_w)^2 \right] \tag{S148}$$

where α_c^* has been adjusted with a factor $\frac{1}{2}$ relative to the original solution in order to account for the sign-constrained weights (44). We have also scaled f_w^* with a factor $\frac{1}{2}$ relative to the original solution. We motivate this with a symmetry argument: The original, unconstrained solution always has a weight distribution that is symmetric and centered at zero, with an equal number of positive and negative weights (5). Intuitively, it is therefore reasonable to expect that a sign-constraint causes precisely half of all weights to have the wrong sign and to be pruned to zero. This has, indeed, been proven to be true at $\max_{f_w} \alpha_c^*(f_w) = 1$ (6), where we have

$$\arg\max_{f_w} \; \alpha_c^*(f_w) = 0.5 \qquad \text{[S149]}$$

and we conjecture that the same applies for all α_c.

S.5. Derivation of sparseness

In the main text, we define sparseness as

$$\text{Sparseness} := \frac{\mathbb{V}[r]}{\mathbb{E}[r^2]} \,. \qquad \text{[S150]}$$

In practice, this metric is applied to a sample of neural stimulus responses, acquired either from simulations or biological experiments. In this case, we replace the variance and expectation with the *unbiased* sample estimates, so that

$$\begin{aligned} \text{Sparseness} &= \frac{n}{n-1} \cdot \frac{\frac{1}{n}\sum r^2 - \left(\frac{1}{n}\sum r\right)^2}{\frac{1}{n}\sum r^2} \\ &= \frac{n}{n-1}\left(1 - \frac{\left(\sum \frac{r}{n}\right)^2}{\sum \frac{r^2}{n}}\right) \\ &= \frac{1-A}{1-1/n} \end{aligned} \qquad \text{[S151]}$$

where n is the number of samples and

$$A = \frac{\left(\sum \frac{r}{n}\right)^2}{\sum \frac{r^2}{n}} \,. \qquad \text{[S152]}$$

This is the way sparseness is formulated in the literature (15, 16).

S.6. Extended synaptic noise analysis

S.6.1. Effect of longer sampling intervals. As observed in the main text, datasets with smaller sample sizes and longer sampling intervals have a scaling exponent that generally increases for depression and decreases for potentiation (i.e., it diverges). This is particularly evident in the case of the longest sampling intervals ($\Delta t \geq 48\,\text{h}$; Fig. 5c, third group of data) where the exponent is 0.38 ± 0.04 for potentiation, and higher than one (1.09 ± 0.03) for depression, consistent with previous analyses of this type (45).

To further verify these observations, we artificially decrease the sampling frequency in each dataset by sub-sampling measurements across time. More specifically, instead of extracting all weight changes over the original sampling interval Δt, we now select only weight changes between measurements separated by an interval $\Delta t_{\text{sub}} = n \cdot \Delta t$, where $n = 2, 3, \ldots, n_{\max}$, and $n_{\max} \cdot \Delta t$ is the total length of the experiment. We then re-compute the scaling exponent as a function of the new sampling interval Δt_{sub} (Suppl. Fig. S11). We find that results *within* datasets corroborate those *across* datasets: as the sampling interval increases, the exponent diverges from ~0.6, by going above one for synaptic depression and decaying close to zero for potentiation. These same trend is found in the simulated data. These findings underscore the importance of acquiring synaptic data with short sampling intervals if one is to accurately estimate scaling parameters for short-term intrinsic synaptic noise fluctuations.

S.6.2. Alternative synaptic noise models. Our model of intrinsic synaptic noise relies on two fundamental assumptions: noise perturbations are *additive* and they affect only *one* sub-synaptic component. The second statement, more specifically, starts from the premise that sub-synaptic constituents are governed by dynamics with substantially different time constants, such that one of the components (u_{ij1}) is much more volatile and fast-changing than the remaining $z-1$ components. This property can be exploited to perform a separation of time scales, whereby short-term synaptic noise (i.e., fluctuations occurring over the course of a few minutes) is modeled as a stochastic perturbation that affects only the fastest component. We motivate this perspective by referencing a series of experimental findings that show that the biochemical signaling cascades that cause structural synaptic plasticity occur in multiple stages, with timescales ranging over multiple orders of magnitude, from milliseconds to hours (46). These dynamics have also been described with the "tagging-and-capture" model (47), which refers to the observation that synapses express plasticity in two stages: first a quick but short-lived form of plasticity that only lasts minutes to hours, followed by a molecular consolidation process that ensures that the change is permanent. The idea of modeling synaptic internal dynamics as two or more components that interact over different time scales has been proposed previously in the computational literature (48–50), albeit not with a *multiplicative* composition.

However, even if one assumes that *all* sub-synaptic components are equally affected by noise, the multi-component synapse model still predicts that intrinsic noise fluctuations scale sublinearly with synaptic weight. To see this, assume that all z components in a synapse are perturbed by i.i.d. stochastic terms $\delta u_1, \ldots, \delta u_z$ drawn from a distribution with $\mathbb{E}_{\delta u}[\delta u] = 0$ and finite variance $\mathbb{V}_{\delta u}[\delta u] = \sigma^2_{\text{noise}}$. The root mean square of the weight fluctuation, computed over perturbation samples, is given by

$$\begin{aligned}
\sqrt{\mathbb{E}_{\delta u}[\delta w^2]} = \sqrt{\mathbb{E}_{\delta u}[(\hat{w} - w)^2]} &= \sqrt{\mathbb{E}_{\delta u}[(\hat{w} - \mathbb{E}_{\delta u}[\hat{w}])^2]} \\
&= \sqrt{\mathbb{V}_{\delta u}[\hat{w}]} = \sqrt{\mathbb{V}_{\delta u}[\hat{u}^z]} \\
&= \sqrt{\mathbb{V}_{\delta u}[(u + \delta u_1) \cdots (u + \delta u_z)]} \\
&= \sqrt{\mathbb{V}_{\delta u}[u^{z-1}(\delta u_1 + \delta u_2 + \ldots) + u^{z-2}(\delta u_1 \delta u_2 + \delta u_1 \delta u_3 + \ldots) + \ldots + (\delta u_1 \cdots \delta u_z)]} \\
&= \sqrt{\textstyle\sum_{k=1}^{z} \binom{z}{k} u^{2(z-k)} \sigma^{2k}_{\text{noise}}} \\
&= \sqrt{\textstyle\sum_{k=1}^{z} \binom{z}{k} w^{2(1-k/z)} \sigma^{2k}_{\text{noise}}} \\
&= w^{1-1/z} \cdot \sqrt{\textstyle\sum_{k=1}^{z} \binom{z}{k} w^{2(1-k)/z} \sigma^{2k}_{\text{noise}}} \; .
\end{aligned} \tag{S153}$$

The last expression is a product of two functions: first $w^{1-1/z}$, which is monotonically increasing, followed by the square root of a polynomial in w with only negative exponents and positive coefficients, which is monotonically decreasing. Denote the first function $f(w)$ and the second $\sqrt{g(w)}$ where g is the polynomial. Since g decreases monotonically, its slope is always negative. Thus, for any pair of weights $0 < w_1 < w_2$, the slope of the root mean square fluctuation in logarithmic space becomes bounded according to

$$\begin{aligned}
\frac{\log(f_2\sqrt{g_2}) - \log(f_1\sqrt{g_1})}{\log(w_2) - \log(w_1)} &= \frac{\log(f_2) - \log(f_1)}{\log(w_2) - \log(w_1)} + \frac{\log(\sqrt{g_2}) - \log(\sqrt{g_1})}{\log(w_2) - \log(w_1)} \\
&= 1 - 1/z + \frac{1}{2}\frac{\log(g_2) - \log(g_1)}{\log(w_2) - \log(w_1)} \\
&< 1 - 1/z \; .
\end{aligned} \tag{S154}$$

In other words, the slope remains sublinear for all $z < \infty$. This result holds true across all magnitudes of w and also implies

$$\sqrt{\mathbb{E}_{\delta u}[\delta w^2]} \sim \mathcal{O}(w^{1-1/z}) \; . \tag{S155}$$

The second fundamental assumption of our noise model is that all perturbations are *additive*. Consider, instead, a model where all z components are perturbed *multiplicatively* by i.i.d. stochastic factors $\delta u_1, \ldots, \delta u_z > 0$, drawn from a distribution with $\mathbb{E}_{\delta u}[\delta u] = 1$ and finite variance. The expected perturbation of the entire weight is now

$$\begin{aligned}
\mathbb{E}_{\delta u}[|\delta w|] = \mathbb{E}_{\delta u}[|\hat{w} - w|] &= \mathbb{E}_{\delta u}[|(u \cdot \delta u_1) \cdots (u \cdot \delta u_z) - u^z|] \\
&= \mathbb{E}_{\delta u}[|(\textstyle\prod_{k=1}^{z} \delta u_k) \cdot u^z - u^z|] \\
&= \mathbb{E}_{\delta u}[|(\textstyle\prod_{k=1}^{z} \delta u_k) - 1| \cdot |u^z|] \\
&= \mathbb{E}_{\delta u}[|(\textstyle\prod_{k=1}^{z} \delta u_k) - 1|] \cdot u^z \\
&= \mathbb{E}_{\delta u}[|(\textstyle\prod_{k=1}^{z} \delta u_k) - 1|] \cdot w \; .
\end{aligned} \tag{S156}$$

Similarly, the root mean square fluctuation becomes

$$\begin{aligned}
\sqrt{\mathbb{E}_{\delta u}[\delta w^2]} = \ldots &= \sqrt{\mathbb{V}_{\delta u}[\hat{u}^z]} \\
&= \sqrt{\mathbb{V}_{\delta u}[(u \cdot \delta u_1) \cdots (u \cdot \delta u_z)]} \\
&= \sqrt{u^{2z} \sigma^{2z}_{\text{noise}}} \\
&= w \cdot \sigma^{z}_{\text{noise}}
\end{aligned} \tag{S157}$$

where the omitted steps are identical to those in Eq. S153. Under both metrics, noise scaling is now purely linear, meaning $\mathcal{O}(w)$. This result does not agree well with experimental data, as one can see in Fig. 5 in the main text. Therefore, at least from an empirical point of view, the multiplicative noise assumption does not have a strong justification. Note, also, that the single-component synapse ($z = 1$) fails to reproduce the scaling of experimental data both when noise is additive and multiplicative.

S.7. Simulation parameters

Table S1. Simulation parameters for consolidation model in Figures 1 and 4.

Parameter	$z = 1$	$z = 2$	$z = 3$	$z = 4$
$\bar{g}$	10^{-4}	5×10^{-3}	7×10^{-3}	7×10^{-3}
$\bar{g}_{\text{inh}}$	10^{-3}	5×10^{-3}	7×10^{-3}	7×10^{-3}
$\bar{u}/z$	10	20	50	50*
$\bar{\beta}$	100	100	100	100

*This is for $f = 0.5$. In simulations with $f < 0.5$, we use $\bar{u}/z = 100$.

Table S2. Simulation parameters for Figure 3. Note that u_0 is the initialization value for all u_{ijk} and that, during sleep, the learning rate $\bar{g}$ increases exponentially with a time constant of 40 replay cycles, where t denotes the cycle.

Parameter	Value
$\bar{g}_{\text{wake}}$	0.017
$\bar{g}$	$[1 + 39 \cdot (1 - \exp(-t/40))] \times 10^{-2}$
u_0	0.6
$\bar{u}/z$	70
$\bar{\beta}$	20

Table S3. Simulation parameters for control model in Figure 4. Note that w_0 is the initialization value for all w_{ij}.

Parameter	$f = 0.5$	$f = 0.05$
$\bar{g}$	10^{-4}	10^{-4}
w_0	0.01	0.01
ρ_0	0.5	1.15

Table S4. Simulation parameters for Figures 5 and 6.

Parameter	Value
σ_{noise}	0.05
u_0	0.1
τ	30
$\mathrm{d}t$	0.005
T_{sample}	1
T_{sim}	1000

S.8. Metadata for synaptic imaging

The following three tables contain details about the experimental data used to produce Figures 5, 6, and S11.

Table S5. Description of synaptic data with large sample sizes and short sampling intervals.

Ref.	System	Δt	Measure	Condition	Datapoints[1]		Weight (%)	
24	Rat Ctx culture	1 h	PSD95 FI	silent	45 600	(43 890)	30.3	(30.5)
				ctrl	39 677	(43 016)	29.0	(35.4)
25	Rat Ctx culture	30 min	PSD95 FI	ctrl	25 847	(25 845)	27.8	(19.3)
26	Mouse Ctx culture	25 min	PSD95 FI	ctrl	9536	(10 347)	6.4	(8.9)
			Munc13 FI	ctrl	9545	(10 353)	6.5	(5.8)

Abbreviations: Ctx = cortex, ACtx = auditory cortex, BCtx = barrel cortex, MCtx = motor cortex, VCtx = visual cortex, PC = pyramidal cell, ad = apical dendrite, FI = fluorescence intensity, SH = spine head, ctrl = control, WT = wild-type, KO = knockout, EE = environmental enrichment.

[1] Total number of $(\Delta\hat{w}, \hat{w})$-pairs. This is determined by the number of imaged synapses and the number of imaging sessions. Left column for potentiation ($\Delta\hat{w} > 0$) and right for depression ($\Delta\hat{w} < 0$).

Table S6. Description of synaptic data with small sample sizes and short to medium sampling intervals. Notation as in Table S5.

Ref.	System	Δt	Measure	Condition	Datapoints		Weight (%)	
28	Mouse MCtx L2/3 PC in vivo	7 h	GluA1 FI	sleep	1039	(1270)	30.2	(28.2)
				wake	346	(405)	9.8	(7.6)
			SH FI	sleep	1107	(1202)	22.3	(19.5)
				wake	371	(380)	7.7	(4.9)
27	Mouse VCtx L5 PC-ad in vivo	10 min	SH FI	WT	238	(237)	3.8	(2.9)
				Fmr1-KO	714	(719)	16.0	(17.1)
29	Mouse VCtx L5 PC-ad in vivo	30 min	PSD95 area	EE	169	(280)	2.6	(8.6)
				ctrl	105	(228)	1.4	(6.5)
			SH area	EE	237	(215)	4.3	(2.4)
				ctrl	161	(169)	1.9	(2.4)

Table S7. Description of synaptic data with long sampling intervals. Notation as in Table S5.

Ref.	System	Δt	Measure	Condition	Datapoints		Weight (%)	
30[1]	Mouse BCtx L2/3/5 in vivo	96 h	Bouton FI	ctrl	12 829	(12 773)	72.0	(57.3)
13	Mouse ACtx L5 PC-ad in vivo	96 h	SH FI	ctrl	2459	(2552)	16.5	(31.3)
27	Mouse VCtx L5 PC-ad in vivo	48 h	SH FI	WT	350	(404)	4.7	(4.2)
				Fmr1-KO	417	(461)	6.0	(6.4)
31	Mouse MCtx L5 PC-ad in vivo	72-96 h	SH area	ctrl	168	(244)	0.8	(0.8)

[1] We included only measurements for which the bouton detection probability was $>90\%$.

S.9. Supplementary figures

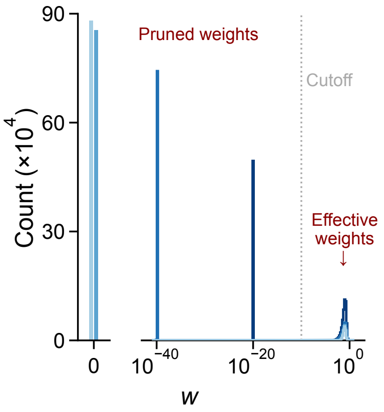

Fig. S1. Extended weight distributions. The same result as in Figure 2f but with an extended abscissa. While the effective weights (that survive consolidation) are in the order of magnitude 10^{-1}, the weights that are pruned end up at exactly zero or within machine precision of zero ($<10^{-20}$). The cutoff used during simulations to consider a weight as pruned is 10^{-10} (dashed gray line).

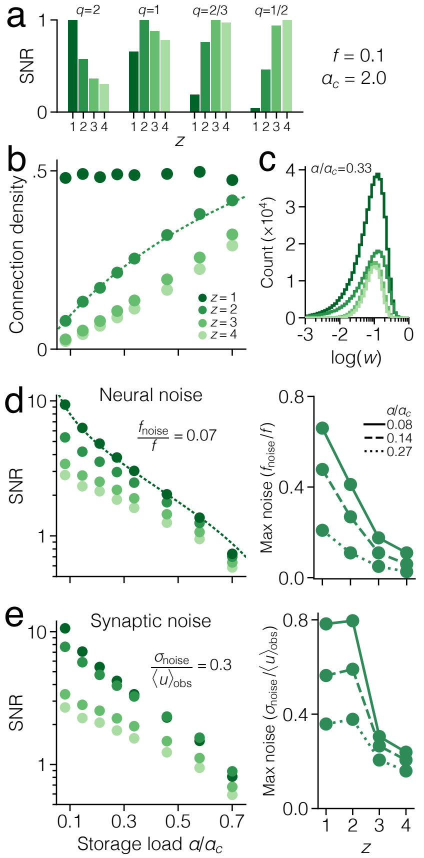

Fig. S2. Simulated consolidation with low-activity patterns. The same type of results as Figure 2 but with $f = 0.1$. **(a)** SNR with respect to noise scaling q, at $\alpha/\alpha_c = 0.08$ (mean over 10^3 neurons). Weights are normalized to $\sum_j w_{ij}^q = 1$ and the maximal SNR, for a given q, is scaled to one. **(b)** Connection density. Dashed line corresponds to theory for $z = 2$. **(c)** Distribution of weights (mean scaled to 10^{-1}). **(d)** SNR with respect to neural noise ($q = 2$; left) and highest level of tolerated neural noise in tests of pattern recall (right). Dashed line corresponds to theory for $z = 1$. **(e)** SNR with respect to synaptic noise ($q = 2 - 2/z$; left) and highest level of tolerated synaptic noise in tests of pattern recall (right).

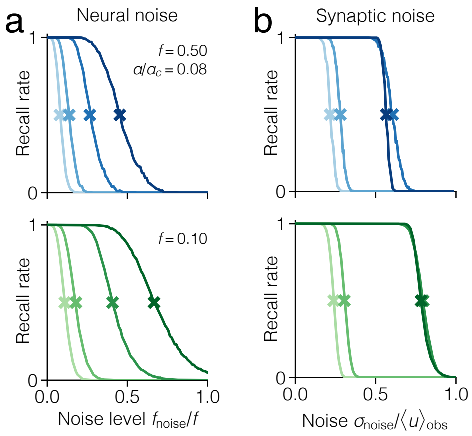

Fig. S3. Empirical robustness evaluation. The fraction of memories that can be successfully retrieved (i.e., recall rate) as a function of **(a)** neural noise and **(b)** synaptic noise, in networks with pattern activity levels $f = 0.5$ (blues) and $f = 0.1$ (greens). Crosses indicate where the recall rate falls below 50%. This defines the highest level of tolerated noise.

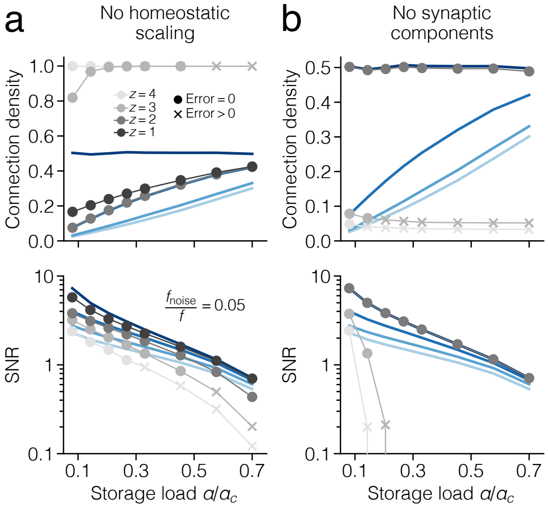

Fig. S4. Ablated consolidation model. Connection density (top) and SNR with respect to neural noise (bottom) after consolidation with ablated (gray) and intact (blue) consolidation model. All markers correspond to means over 10^3 neurons, but circles indicate cases where the network manages to find a solution with $E = 0$ in 2×10^6 replay cycles, while crosses indicate cases where the network fails to find such solutions. **(a)** Consolidation without homeostatic scaling. With the exception of $z = 2$, the network fails to converge to any meaningful results. Simulation parameters as in Figure 2. **(b)** Consolidation with homeostatic scaling but only single-factor synapses (i.e., $z = 1$). Due to the multiplicative projected gradient ascent, the solution either coincides with the intact $z = 1$ solution, or, once again, fails to converge to anything meaningful. Simulation parameters as in Figure 2, but with learning rates $\bar{g} = 10^{-4}$ and $\bar{g}_{\text{inh}} = 10^{-3}$.

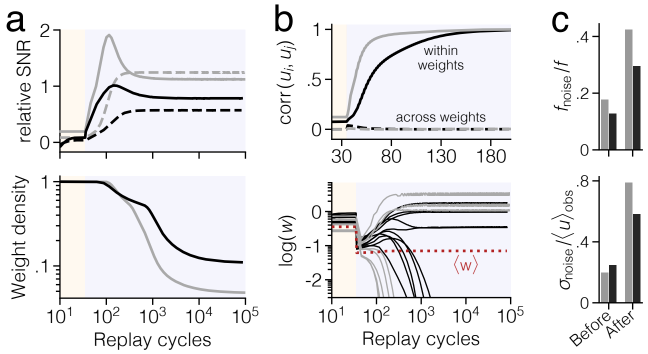

Fig. S5. Memory formation and stabilization in wakefulness and sleep. Simulation of wakefulness (yellow background) and sleep (violet background) with high load (black; $\alpha = 0.44$) and low load (gray; $\alpha = 0.2$). **(a)** Relative SNR (top) and weight density (bottom) over replay cycles. Solid curves represent neural noise ($q = 2$) and dashed curves synaptic noise ($q = 1$). Scaling of the SNR-axis is arbitrary. **(b)** Top panel shows the pairwise Pearson correlation between subsynaptic components u_{ijk} within the same weight (same j, different k) and across different weights (same k, different j). Bottom panel shows the weight trace for a subset of synapses. **(c)** Maximum tolerated neural and synaptic noise before and after sleep.

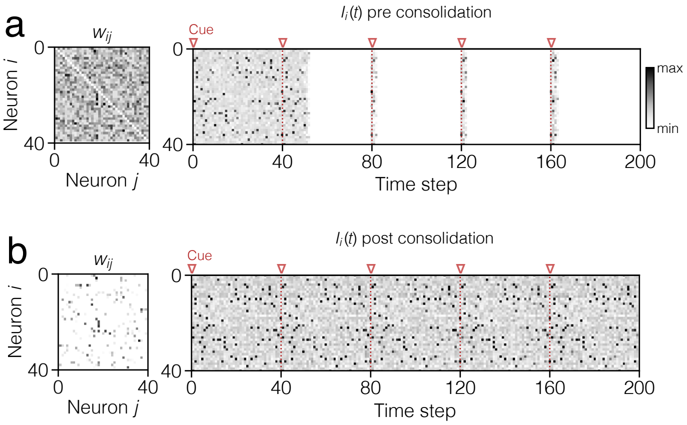

Fig. S6. Formation and consolidation of sequence memory. (a) Weight matrix (left) and input current (right) of 40 neurons during pattern recall, before consolidation ($f = 0.05, \alpha = 0.4$). The network has been trained to store a sequence of memory patterns using a slightly modified variant of the few-shot learning rule in Eq. S57, namely $\Delta u_{ij1} = \bar{g}_{\text{new}} \bar{g}_{\text{wake}} (\xi_i^{\mu+1} - f)(\xi_j^{\mu} - f) u_{ij2}$. The network receives the same cue every 40 steps and is then simulated for 40 steps. Synaptic noise starts after 50 steps. **(b)** Same as **a**, but after having applied our consolidation model (without any changes).

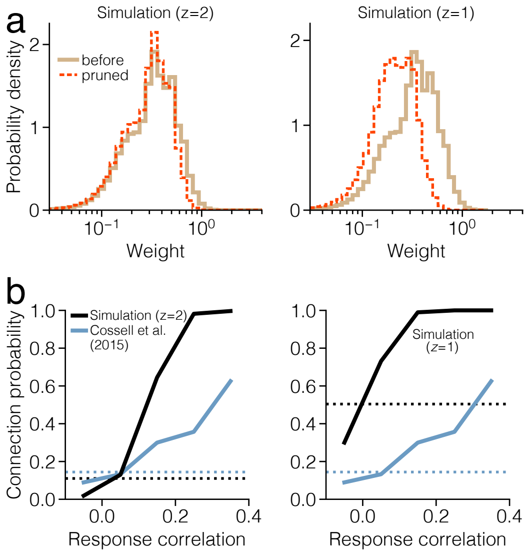

Fig. S7. Comparison of dense and sparse consolidation. Simulation of wakefulness (few-shot learning) and sleep (consolidation) in a network with $z = 2$ (left column) and $z = 1$ (right column), using low-activity patterns $f = 0.05$ at storage load $\alpha = 0.44$. **(a)** Distribution of pre-sleep and pruned weights. Networks with $z = 1$ always converge to a dense connectivity in which roughly 50% of weights have been pruned, while networks with $z = 2$ prune a much larger fraction of weights and therefore generate a sparse connectivity (roughly 10% in this case). In other words, the vast majority of pre-sleep weights are pruned when $z = 2$, which manifests itself as a high degree of overlap between the pre-sleep and pruned distributions. For $z = 1$, however, only about half of pre-sleep weights are pruned, which leads to a low degree of overlap between the distributions. **(b)** Connection probability as a function of response correlation. Networks with $z = 1$ (right, black) converge to dense solutions with roughly 50% connection probability and can therefore not reproduce the low level of connection probability observed in, for example, rodent visual cortex (blue) (14). Such sparse connectivity can, on the other hand, be produced in networks with $z = 2$ (left, black).

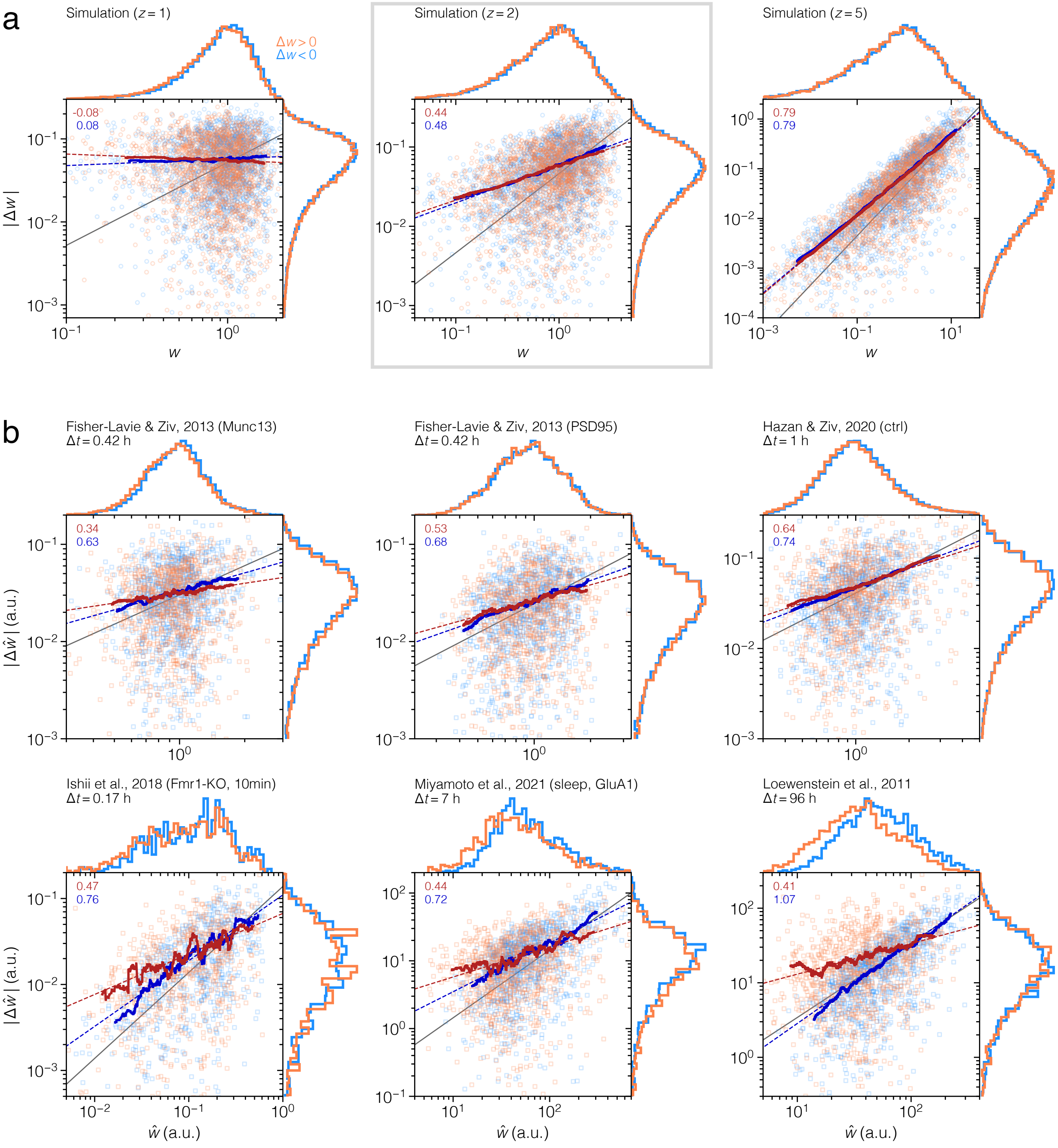

Fig. S8. Extended synaptic fluctuation data. (a) Absolute weight change as a function of initial weight in simulated data with $z = 1$ (left), $z = 2$ (middle), and $z = 5$ (right), for potentiation (orange) and depression (blue). Solid lines are moving averages, and dashed lines are linear fits to the solid lines (slope value shown in upper left corner). The straight solid lines suggest a power-law in the original data, and their slope (i.e., the power-law exponent) approximately obeys the scaling law $q = 1 - 1/z$. The identity line (gray) has slope 1, and is included for comparison. **(b)** The same type of plot as in **a**, but for experimental measurements of dendritic spine sizes in cortical neurons, across different datasets. The sampling time is denoted with Δt.

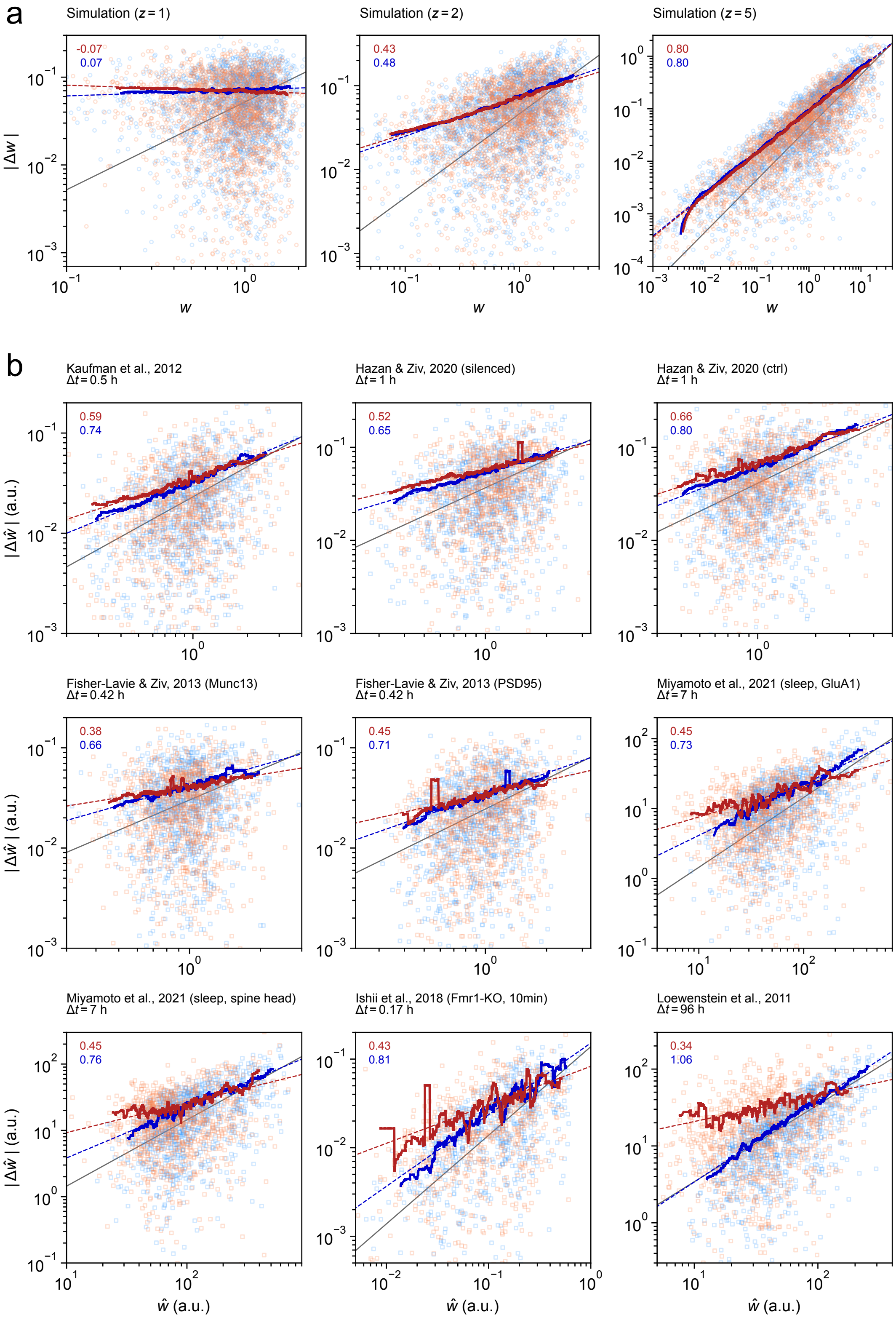

Fig. S9. Alternative synaptic fluctuation metrics. (a) Absolute weight change as a function of initial weight in simulated data with $z = 1$ (left), $z = 2$ (middle), and $z = 5$ (right), for potentiation (orange) and depression (blue). Solid lines correspond to the root mean square using a sliding window, and dashed lines are linear fits to the solid lines (slope value shown in upper left corner). The identity line (gray) has slope 1 and is included for comparison. **(b)** The same type of plot as in **a**, but for experimental measurements of dendritic spine sizes in cortical neurons, across different datasets. The sampling time is denoted with Δt.

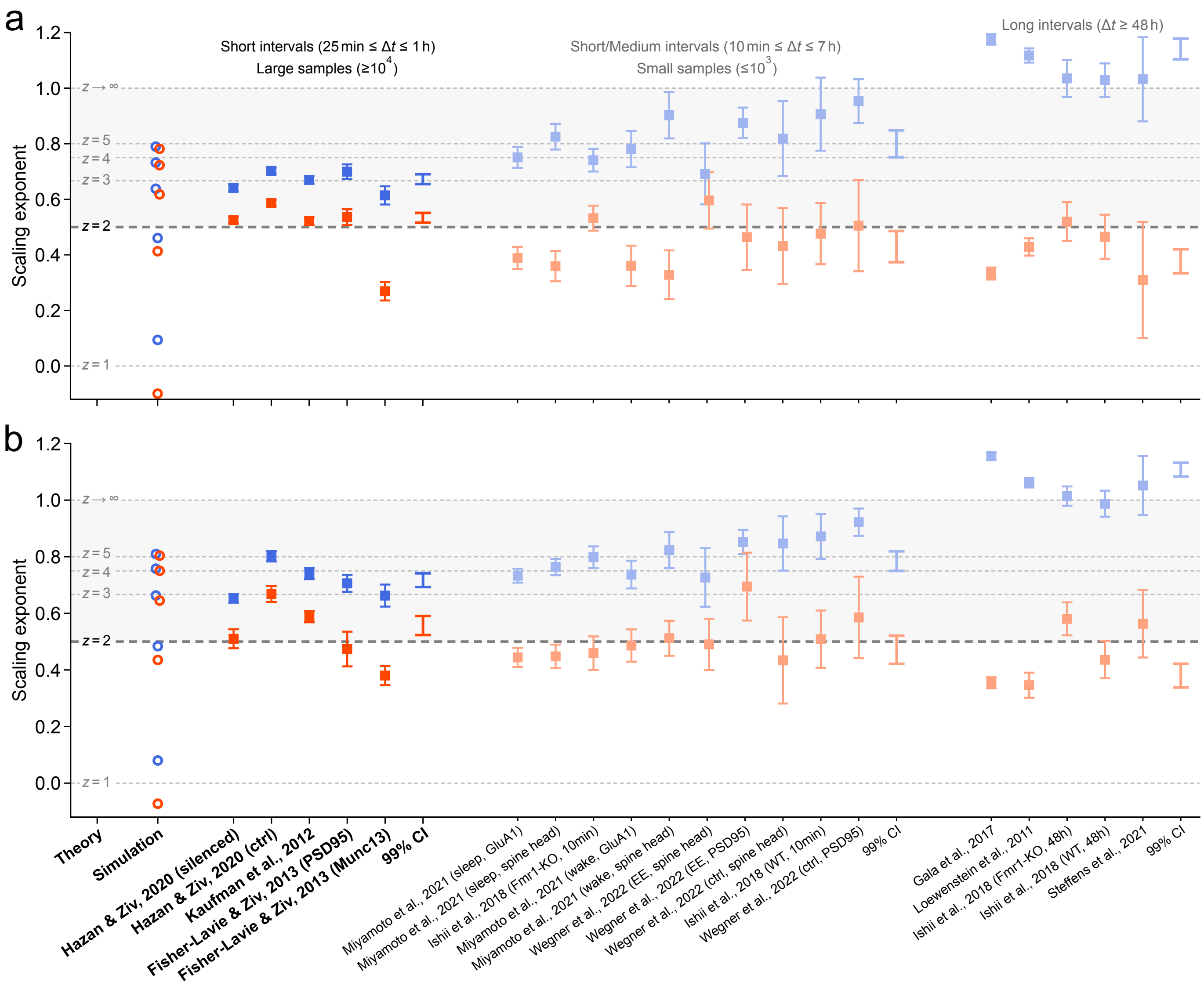

Fig. S10. Scaling under alternative fluctuation metrics. The scaling exponent of synaptic noise fluctuations in simulated (circles) and experimental data (squares; mean ± SE). The exponent corresponds to the slope of the data in logarithmic space. In contrast to the results presented in the main text, the slopes have in this case been computed with bootstrapped linear regression applied **(a)** directly to all data points without any averaging and **(b)** to the root mean square deviation. Bootstrapping was in both cases performed with 1000 repetitions in the empirical datasets and 100 repetitions in the synthetic datasets. Labels on the abscissa contain a publication reference and a brief methodological descriptor; complete details are provided in Tables S5, S6, and S7.

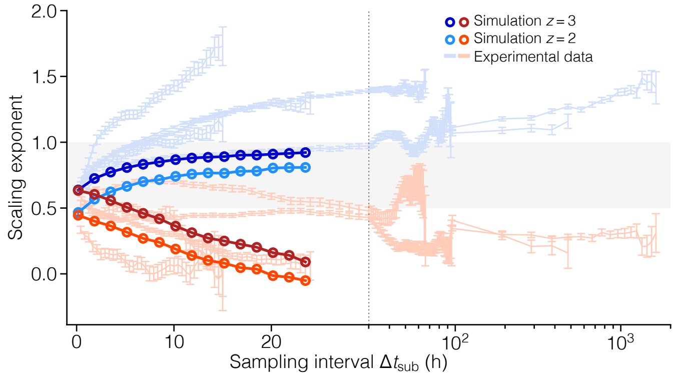

Fig. S11. Synaptic noise scaling in subsampled data. The scaling exponent of synaptic fluctuations as a function of the sampling interval Δt_{sub}, in simulated and experimental data (13, 24–26, 30) (mean $\pm$ SE, estimated as in Fig. 5). The sampling interval is artificially lengthened by sub-sampling datapoints across time. The scaling exponent generally diverges by increasing for depression (blue markers) and decreasing for potentiation (orange markers).

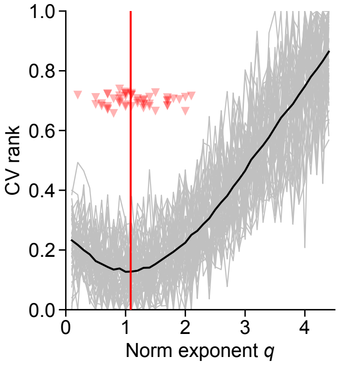

Fig. S12. Bootstrapping the CV of spine size norms. The CV of norms, ranked from zero to one, as a function of q, using dendritic spine sizes from pyramidal cells in rodent cortex (25), where gray curves show the result of 50 bootstrapped samples and red triangles show the location of the minimum of each curve (q_{min}). The black line is the average of 1000 bootstrapped CV-curves and the red line shows the location of the average of 1000 bootstrapped q_{min}.

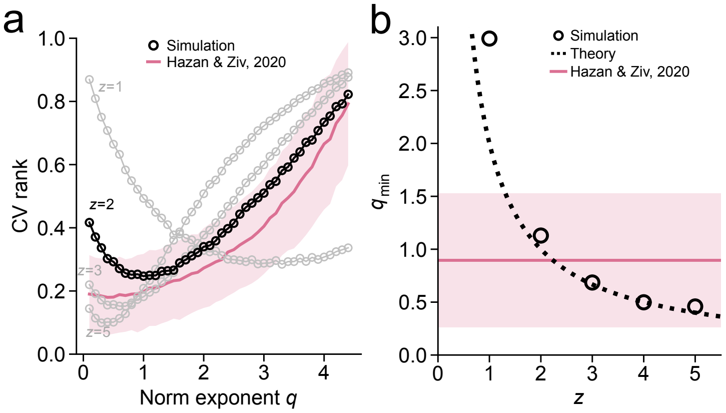

Fig. S13. CV of spine size norms in second dataset. (a) The CV of norms, ranked from zero to one, as a function of q, using simulated data (black, gray) and dendritic spine sizes (pink) from pyramidal cells in rodent cortex (mean $\pm$ SE, bootstrap of 1000 samples). The experimental data comes from Hazan and Ziv (24) and was measured under pharmacological blocking of glutamatergic signaling (i.e., silent condition in Table S5). Results from the experimental data are noisier and a clear kink in the pink curve is not visible, but it is best matched by the two-factor model ($R^2 = 0.44$ for $z = 2$, compared to second best $R^2 = 0.31$ for $z = 3$). **(d)** The q-value at which the CV is minimized ($q_{\min}$). The estimate for experimental data (0.90 ± 0.63) is, again, noisy, but its average is closest to the theoretical prediction for $z = 2$.

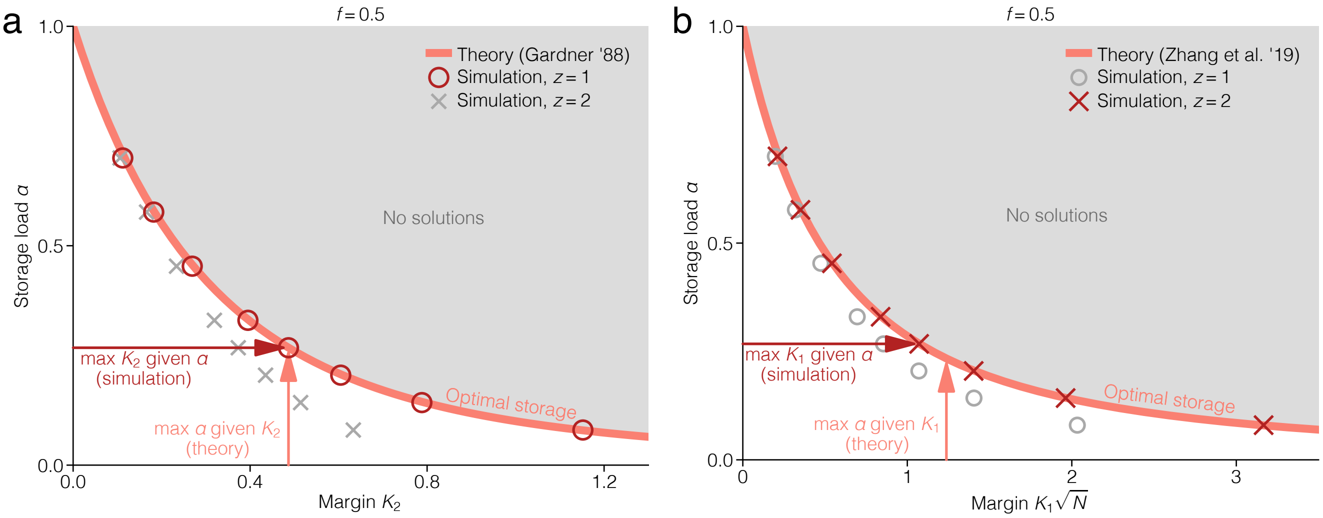

Fig. S14. Comparison of the max-margin and max-storage formalisms. (a) Storage load α as a function of margin K_2, shorthand for $K(q=2)$. The optimal storage curve is the function $\alpha^*(K, f, q)$ with $f = 0.5$ and $q = 2$, as reported by Gardner (4). This is obtained by solving the *storage problem* in Eq. S92, where α is maximized, given a fixed margin (pink arrow) in the mean-field limit. The same optimal storage configuration can also be found by solving the corresponding *max-margin problem* in Eq. S91, where K instead is maximized, given a fixed load (brown arrow). This is what our consolidation model is derived to do. Indeed, it retrieves the solution when $z = 1$, as this maximizes K_2, but not when $z = 2$, as this maximizes K_1. **(b)** Storage load α as a function of margin K_1, shorthand for $K(q=1)$. The optimal storage curve is the function $\alpha^*(K, f, q)$ with $f = 0.5$ and $q = 1$, as formulated by Zhang et al. (7). Using our consolidation model, we now find the optimal storage solution when $z = 2$, as this maximizes K_1, but not when $z = 1$, as this maximizes K_2. The simulation results are the same as in Figure 1.

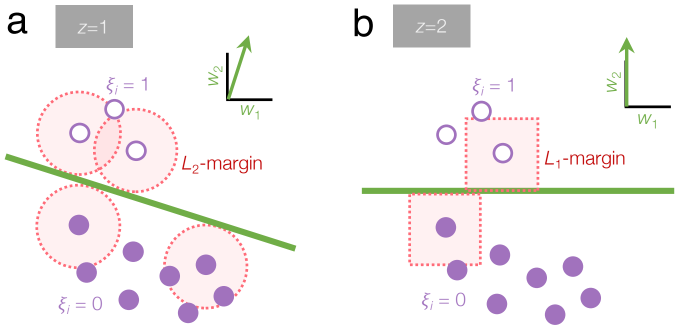

Fig. S15. Dense and sparse consolidation in neural state space. In a network of $N = 3$ neurons, we consider a single neuron i and observe the state space of its two neighboring neurons. The weight vector $\boldsymbol{w}_i = (w_1, w_2)$ and the inhibition $I_{\mathrm{inh},i}$ define a linear classification boundary (green) that separate all patterns (circles) according to the labels $\xi_i = 1$ (white) and $\xi_i = 0$ (purple). For the sake of simplifying the illustration, we use real-valued patterns, but the same argument holds for the binary case. **(a)** Consolidation with $z = 1$ is equivalent to a maximization of the L_2-margin, which means that the L_2-distance between the boundary and the nearest patterns is maximized (red circles). The solution is typically dense, which means that $w_1^*, w_2^* > 0$. **(b)** Consolidation with $z = 2$ is equivalent to a maximization of the L_1-margin, which means that the L_∞-distance between the boundary and the nearest patterns is maximized (34) (red squares). The boundary is now forced to align with the one of the coordinate axes, thus rendering the solution sparse, such that $w_1^* = 0$ and $w_2^* > 0$.

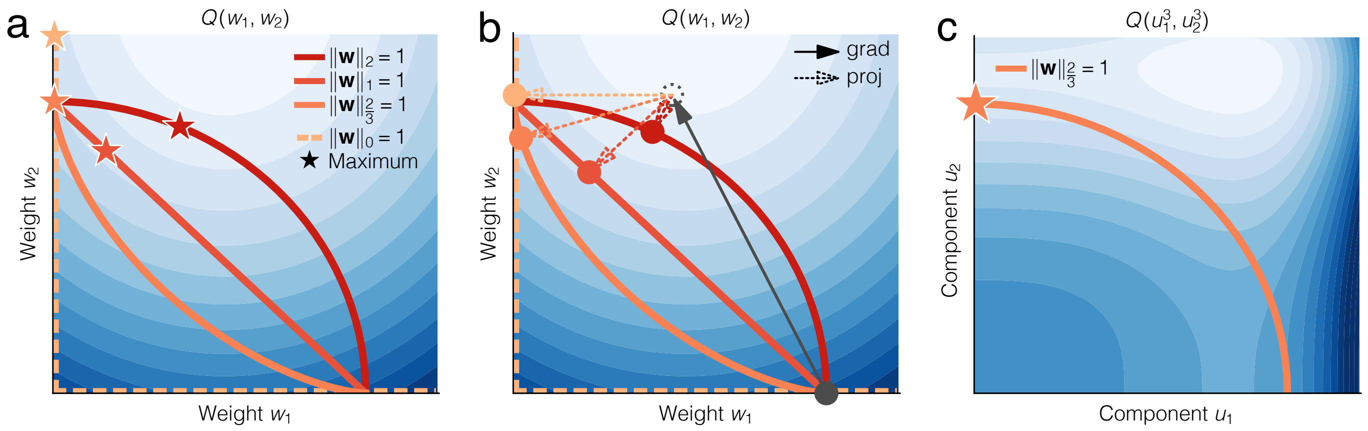

Fig. S16. Dense and sparse consolidation in the loss landscape. **(a)** The landscape of the objective function $\mathcal{Q}(w_1, w_2)$ (blue; lighter hues closer to max) together with the feasible set under constraints of type $\|\boldsymbol{w}\|_q = 1$ (orange curves). In general, a lower q pushes the optimal weight vector (star) closer to a sparse configuration, in which $w_1^* = 0$ and $w_2^* > 0$. Indeed, for $q < \frac{2}{3}$, the solution is sparse. **(b)** Projected gradient descent in the $\mathcal{Q}$-landscape involves first a gradient step (solid arrow), followed by a projection to the feasible set (dashed arrow). The projection can be multiplicative $(q = 2)$, additive $(q = 1)$, or a hard thresholding $(q = 0)$. However, for fractional norms $(0 < q < 1)$, the projection is generally anisotropic, which means that weights are adjusted by different amounts, depending on their relative size to each other. **(c)** We can make the projection to any fractional norm curve multiplicative, by performing the optimization in the re-parameterized landscape $\mathcal{Q}(u_1^z, u_2^z)$, if we choose the number of components $z = 2/q$. For example, projections to $\|\boldsymbol{w}\|_{\frac{2}{3}}$ (orange curve) become multiplicative with $z = 3$. The optimum remains sparse, with $u_1^* = 0$ and $u_2^* > 0$.

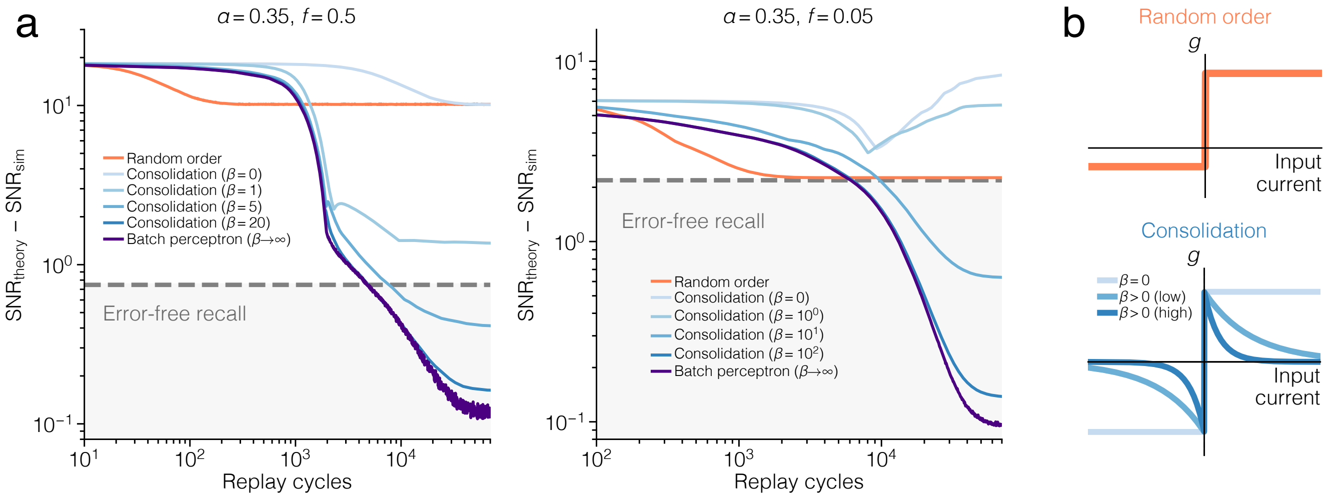

Fig. S17. Consolidation with slow and fast gating function decay. (a) Difference in neural noise SNR between the theoretical optimum and the solution found by consolidating with $z = 1$ and varying β-values, in a single neuron (lower is better). The orange curve represents "wakeful" learning, where patterns are presented in random order and weights are updated with $\Delta w_{ij} = \bar{g}(\xi_i^\mu - f)\xi_j^\mu$. Light blue curves represent our consolidation algorithm. The dark purple curve represents our consolidation in the limit $\beta \to \infty$, which is equivalent to the batch perceptron (3). The dashed line indicates where the simulation crosses $\mathrm{SNR}_{\mathrm{sim}} = 0$, which is where $E = 0$ is reached. Scaling of the ordinate is arbitrary. Simulation parameters: $\bar{g} = 10^{-4}$, $\bar{w} = 1$, and $I_{\mathrm{inh}} = 8.5$ for $f = 0.5$ ($I_{\mathrm{inh}} = 1.4$ for $f = 0.05$). **(b)** Qualitative comparison of the shape of the gating function g, for the different variants of consolidation.

References

1. R Rubin, LF Abbott, H Sompolinsky, Balanced excitation and inhibition are required for high-capacity, noise-robust neuronal selectivity. *Proc. Natl. Acad. Sci. USA* **114**, E9366–E9375 (2017).
2. MS Nacson, et al., Convergence of gradient descent on separable data in *Proceedings of the 22nd International Conference on Artificial Intelligence and Statistics*, Proceedings of Machine Learning Research. Vol. 89, pp. 3420–3428 (2019) ISSN: 2640-3498.
3. W Krauth, M Mezard, Learning algorithms with optimal stability in neural networks. *J. Phys. A: Math. Gen.* **20**, L745–L752 (1987).
4. E Gardner, The space of interactions in neural network models. *J. Phys. A: Math. Gen.* **21**, 257–270 (1988).
5. M Bouten, A Engel, A Komoda, R Serneels, Quenched versus annealed dilution in neural networks. *J. Phys. A: Math. Gen.* **23**, 4643 (1990).
6. HW Yau, PhD thesis (University of Edinburgh, Edinburgh, Scotland) (1992).
7. D Zhang, C Zhang, A Stepanyants, Robust associative learning is sufficient to explain the structural and dynamical properties of local cortical circuits. *J. Neurosci.* **39**, 6888–6904 (2019).
8. MV Tsodyks, MV Feigel'man, The enhanced storage capacity in neural networks with low activity level. *Eur. Lett.* **6**, 101–105 (1988).
9. N Brunel, V Hakim, P Isope, JP Nadal, B Barbour, Optimal information storage and the distribution of synaptic weights: perceptron versus Purkinje cell. *Neuron* **43**, 745–757 (2004).
10. A Alemi, C Baldassi, N Brunel, R Zecchina, A three-threshold learning rule approaches the maximal capacity of recurrent neural networks. *PLoS Comput. Biol.* **11**, e1004439 (2015).
11. N Brunel, Is cortical connectivity optimized for storing information? *Nat. Neurosci.* **19**, 749–755 (2016).
12. J Chapeton, T Fares, D LaSota, A Stepanyants, Efficient associative memory storage in cortical circuits of inhibitory and excitatory neurons. *Proc. Natl. Acad. Sci. USA* **109**, E3614–E3622 (2012).
13. Y Loewenstein, A Kuras, S Rumpel, Multiplicative dynamics underlie the emergence of the log-normal distribution of spine sizes in the neocortex in vivo. *J. Neurosci.* **31**, 9481–9488 (2011).
14. L Cossell, et al., Functional organization of excitatory synaptic strength in primary visual cortex. *Nature* **518**, 399–403 (2015).
15. ET Rolls, MJ Tovee, Sparseness of the neuronal representation of stimuli in the primate temporal visual cortex. *J. Neurophysiol.* **73**, 713–726 (1995).
16. WE Vinje, JL Gallant, Sparse coding and decorrelation in primary visual cortex during natural vision. *Science* **287**, 1273–1276 (2000).
17. L Woloszyn, DL Sheinberg, Effects of long-term visual experience on responses of distinct classes of single units in inferior temporal cortex. *Neuron* **74**, 193–205 (2012).
18. KM Fenn, DZ Hambrick, Individual differences in working memory capacity predict sleep-dependent memory consolidation. *J. Exp. Psychol. Gen.* **141**, 404 (2012).
19. KM Fenn, DZ Hambrick, General intelligence predicts memory change across sleep. *Psychon. Bull. Rev.* **22**, 791–799 (2015).
20. JE Ashton, SA Cairney, Future-relevant memories are not selectively strengthened during sleep. *PLoS ONE* **16**, e0258110 (2021).
21. TD Wickens, *Elementary signal detection theory.* (Oxford University Press), (2002).
22. CKE Jung, J Herms, Structural dynamics of dendritic spines are influenced by an environmental enrichment: an in vivo imaging study. *Cereb. Cortex* **24**, 377–384 (2014).
23. P Berkes, B White, J Fiser, No evidence for active sparsification in the visual cortex in *Advances in Neural Information Processing Systems 22.* (2009).
24. L Hazan, NE Ziv, Activity dependent and independent determinants of synaptic size diversity. *J. Neurosci.* **40**, 2828–2848 (2020).
25. M Kaufman, MA Corner, NE Ziv, Long-term relationships between cholinergic tone, synchronous bursting and synaptic remodeling. *PLoS ONE* **7**, e40980 (2012).
26. A Fisher-Lavie, NE Ziv, Matching dynamics of presynaptic and postsynaptic scaffolds. *J. Neurosci.* **33**, 13094–13100 (2013).
27. K Ishii, et al., In vivo volume dynamics of dendritic spines in the neocortex of wild-type and Fmr1 KO mice. *eNeuro* **5**, e0282–18.2018 (2018).
28. D Miyamoto, W Marshall, G Tononi, C Cirelli, Net decrease in spine-surface GluA1-containing AMPA receptors after post-learning sleep in the adult mouse cortex. *Nat. Commun.* **12**, 2881 (2021).
29. W Wegner, H Steffens, C Gregor, F Wolf, KI Willig, Environmental enrichment enhances patterning and remodeling of synaptic nanoarchitecture as revealed by STED nanoscopy. *eLife* **11**, e73603 (2022).
30. R Gala, et al., Computer assisted detection of axonal bouton structural plasticity in in vivo time-lapse images. *eLife* **6**, e29315 (2017).
31. H Steffens, et al., Stable but not rigid: chronic in vivo STED nanoscopy reveals extensive remodeling of spines, indicating multiple drivers of plasticity. *Sci. Adv.* **7**, eabf2806 (2021).
32. C Cortes, V Vapnik, Support-vector networks. *Mach. Learn.* **20**, 273–297 (1995).

33. HM Köhler, D Widmaier, Sign-constrained linear learning and diluting in neural networks. *J. Phys. A: Math. Gen.* **24**, L495–L502 (1991).
34. OL Mangasarian, Arbitrary-norm separating plane. *Oper. Res. Lett.* **24**, 15–23 (1999).
35. E Oja, Simplified neuron model as a principal component analyzer. *J. Math. Biol.* **15**, 267–273 (1982).
36. GG Turrigiano, KR Leslie, NS Desai, LC Rutherford, SB Nelson, Activity-dependent scaling of quantal amplitude in neocortical neurons. *Nature* **391**, 892–896 (1998).
37. KD Miller, DJC MacKay, The role of constraints in Hebbian learning. *Neural Comput.* **6**, 100–126 (1994).
38. G Chechik, I Meilijson, E Ruppin, Synaptic pruning in development: a computational account. *Neural Comput.* **10**, 1759–1777 (1998).
39. G Chechik, I Meilijson, E Ruppin, Neuronal regulation: a mechanism for synaptic pruning during brain maturation. *Neural Comput.* **11**, 2061–2080 (1999).
40. J Sacramento, A Wichert, MCW van Rossum, Energy efficient sparse connectivity from imbalanced synaptic plasticity rules. *PLoS Comput. Biol.* **11**, e1004265 (2015).
41. F Zenke, S Ganguli, SuperSpike: supervised learning in multilayer spiking neural networks. *Neural Comput.* **30**, 1514–1541 (2018).
42. A Renart, P Song, XJ Wang, Robust spatial working memory through homeostatic synaptic scaling in heterogeneous cortical networks. *Neuron* **38**, 473–485 (2003).
43. T Toyoizumi, M Kaneko, M Stryker, K Miller, Modeling the dynamic interaction of Hebbian and homeostatic plasticity. *Neuron* **84**, 497–510 (2014).
44. DJ Amit, C Campbell, KYM Wong, The interaction space of neural networks with sign-constrained synapses. *J. Phys. A: Math. Gen.* **22**, 4687–4693 (1989).
45. A Morrison, A Aertsen, M Diesmann, Spike-timing-dependent plasticity in balanced random networks. *Neural Comput.* **19**, 1437–1467 (2007).
46. J Nishiyama, R Yasuda, Biochemical computation for spine structural plasticity. *Neuron* **87**, 63–75 (2015).
47. RL Redondo, RGM Morris, Making memories last: the synaptic tagging and capture hypothesis. *Nat. Rev. Neurosci.* **12**, 17–30 (2011).
48. C Clopath, L Ziegler, E Vasilaki, L Büsing, W Gerstner, Tag-trigger-consolidation: a model of early and late long-term-potentiation and depression. *PLoS Comput. Biol.* **4**, e1000248 (2008).
49. MK Benna, S Fusi, Computational principles of synaptic memory consolidation. *Nat. Neurosci.* **19**, 1697–1706 (2016).
50. HL Li, MCW van Rossum, Energy efficient synaptic plasticity. *eLife* **9**, e50804 (2020).
